# Multi-Omics Analysis of a Spontaneous Type 2 Diabetes Model in *Myodes rufocanus* and Its Underlying Mechanisms

**DOI:** 10.3390/ijms27031539

**Published:** 2026-02-04

**Authors:** Ijaz Ullah, Haseena Mujeeb, Qindan Li, Xingxuan Zhou, Habib Alam, Mujeeb Ur Rahman, Yanan Zhao, Jiazheng Zhou, Qingying Wang, Sanpin Luo, Liang Wang, Jingyu Wang

**Affiliations:** 1 Laboratory Animal Centre, Dalian Medical University Dalian, Dalian 116044, China; mijazk9@gmail.com (I.U.);; 2 College of Basic Medical Sciences, Dalian Medical University, No. 9 West Section, South Lvshun Road, Dalian 116044, China; 3 Department of Biotechnology, College of Basic Medical Science, Dalian Medical University, Dalian 116044, China; mujeeb166@gmail.com; 4 Comparative Medicine Department of Research and Teaching, Dalian Medical University, Dalian 116044, China

**Keywords:** spontaneous diabetes, *Myodes rufocanus*, COX14, mitochondrial integrity, oxidative stress, insulin resistance, β-cell dysfunction

## Abstract

Diabetes mellitus (DM) is a chronic metabolic disorder characterized by persistent hyperglycemia, progressive β-cell dysfunction, and insulin resistance. While numerous chemically induced and transgenic rodent models exist, spontaneous models recapitulating natural type 2 diabetes mellitus (T2DM) progression remain scarce. Here, we characterize *Myodes rufocanus* as a novel spontaneous T2DM model through comprehensive assessments of 18-week-old male F6 voles, demonstrating hallmark diabetic features including weight gain, hyperphagia, polydipsia, hyperglycemia, insulin resistance, and dyslipidemia. Pancreatic transcriptomic profiling revealed pronounced COX14 (cytochrome c oxidase assembly factor 14) downregulation, as validated by qPCR and Western blotting in pancreatic tissue and MIN6 β-cells. MIN6 cells under chronic high-glucose conditions (30 mM) exhibited diminished mitochondrial membrane potential, impaired ATP biosynthesis, elevated reactive oxygen species, and attenuated glucose-stimulated insulin secretion, with consistent COX14 downregulation suggesting potential association with mitochondrial dysfunction. Additionally, suppressed Nrf2–HO-1 antioxidant signaling appeared to compound cellular injury, with intrinsic apoptotic pathway activation indicated by elevated Bax/Bcl-2 ratios and caspase-3 activity. These findings establish M. rufocanus as a valuable spontaneous T2DM model and implicate COX14 downregulation as a potential correlate of mitochondrial impairment and β-cell failure in diabetes pathogenesis.

## 1. Introduction

Diabetes has emerged as one of the most rapidly proliferating public health issues in the 21st era among metabolic disorders and is characterized by elevated blood glucose levels [1]. This severe, chronic, non-communicable disease is second only in prevalence in cardiocerebrovascular conditions. The consequences of diabetes significantly affect the quality of life of patients and impose a substantial burden on medical care systems with an increase in the mortality rate [2,3]. Complications of diabetes, including renal dysfunction, cardiac complications, foot lesions, peripheral nerve impairment, and ocular abnormalities, are more detrimental than the primary condition itself, affecting diverse organ systems throughout the body [3,4].

The incidence of diabetes worldwide has exhibited a substantial increase, from 4.6% in 2000 to 10.5% in 2021, with the affected population reaching 537 million and projected to rise by 2045 to 784 million [1]. Diabetes is categorized into four primary classifications: type 1 diabetes mellitus (T1D), type 2 diabetes mellitus (T2D), gestational diabetes mellitus, and particular types such as maturity-onset diabetes of the young [5]. T2D accounts for about 90% of diabetes cases worldwide, whereas type 1 diabetes (T1D) and other types comprise the remainder [6,7]. Regulation of glucose homeostasis is fundamental for optimal cellular function, relying on an intricate network of hormones and neuropeptides secreted by diverse organs and tissues, with the pancreas playing a central role. Perturbation of this equilibrium leads to hyperglycemia, a characteristic feature of diabetes mellitus [8,9], which results from compromised action or secretion of insulin, or both [10]. The underlying molecular mechanisms of diabetes include complex interrelated factors and pathways. Understanding these mechanisms is essential for developing targeted therapies and effectively managing diabetes and its complications [11]. However, the precise etiology and pathogenesis of diabetes remain obscure due to the intricacy of whole-body systems [3].

Animal models have played a crucial role in diabetes research, significantly enhancing our understanding of the molecular processes that drive the disease. Larger animals, such as non-human primates, present specific ethical and practical challenges related to housing, cost, and maintenance [12]. Murine, especially rats and mice, are the most frequently used specimens because of their genetic similarity to humans and ease of experimental manipulation. These models include drug-induced, high-fat diet-induced, genetic, genetically engineered, and spontaneous types [12,13,14]. Traditional rodent models, whether pharmacologically induced, genetically modified, or diet-induced, have been pivotal in advancing our understanding of the pathophysiology of diabetes. However, these models exhibit inherent limitations in accurately mirroring the chronic, progressive, and multifaceted nature of human type 2 diabetes mellitus (T2DM). Models induced by chemicals such as streptozotocin or alloxan, monogenic obesity models such as leptin-deficient ob/ob and leptin receptor-deficient db/db mice, and those induced by high-fat diets face significant translational challenges due to differences in disease etiology, β-cell regeneration capacity, and metabolic characteristics compared to human T2DM [15]. Although each model has its strengths and limitations, spontaneous diabetes rodent models, like NOD mice and BB rats, closely approximate human diabetes pathogenesis. By accurately replicating natural disease progression, these models provide researchers with the means to delve into the complex interplay between genetic, immune, and ecological factors in diabetes development. Their substantial contributions to the understanding of diabetes pathophysiology have established them as invaluable tools for evaluating novel therapeutic strategies and exploring the underlying biological mechanisms [16].

In this context, *Myodes rufocanus* (also known as *Craseomys rufocanus*), commonly referred to as the gray-sided vole or the brown-backed vole, inhabits northern regions of the Euro-Asiatic continent [17]. This species has attracted scientific interest because of its high incidence of spontaneous diabetes, with reported prevalence rates of up to 43% [18]. However, research on the molecular mechanisms underlying diabetes in this species remains scarce. Transcriptomics, a crucial component of multi-omics technologies, has significantly advanced our understanding of the molecular basis of diabetes mellitus (DM). Through transcriptomic analysis, researchers can identify genes with altered expression levels, elucidate relevant molecular pathways, and detect cell-specific changes associated with diabetes and their associated complications. This approach has proven instrumental in identifying promising therapeutic interventions and novel disease signatures [19,20].

This study aimed to characterize the pancreatic mRNA transcriptome of 18-week-old male F6 *Myodes rufocanus*, in which spontaneous type 2 diabetes was established and confirmed through physiological and biochemical assessments, including weight gain, hyperphagia, polydipsia, hyperglycemia, insulin resistance, and dyslipidemia. This was followed by transcriptomic profiling and functional validation in MIN6 β cells. Using the Illumina NovaSeq™ X Plus platform, we identified genes exhibiting differential expression levels and investigated their potential functions through enrichment analysis using GO and KEGG pathway analyses, along with the evaluation and validation of key hub genes and their associated pathways. This investigation aimed to deepen the current understanding of the molecular mechanisms underlying diabetes mellitus (DM) pathogenesis, potentially contributing to the advancement of new therapeutic approaches for DM management.

## 2. Results

The global incidence of diabetes is increasing at an unprecedented rate, reaching epidemic proportions and significantly affecting the morbidity and mortality rates. The initiation and development of diabetes and its associated consequences are influenced by various factors, including epigenetic changes, inflammatory processes, oxidative stress, and mitochondrial dysfunction. In this study, RNA sequencing and de novo transcriptome assembly of pancreatic samples from *Myodes rufocanus*, a species that naturally develops diabetes, were performed to explore the molecular mechanisms underlying diabetes progression. This study aimed to develop targeted therapeutic strategies to mitigate the impact of DM-related diseases and fatality in individuals with DM.

### 2.1. Pedigree Analysis: Hereditary Transmission of Diabetes in Myodes rufocanus

**Figure 1:**
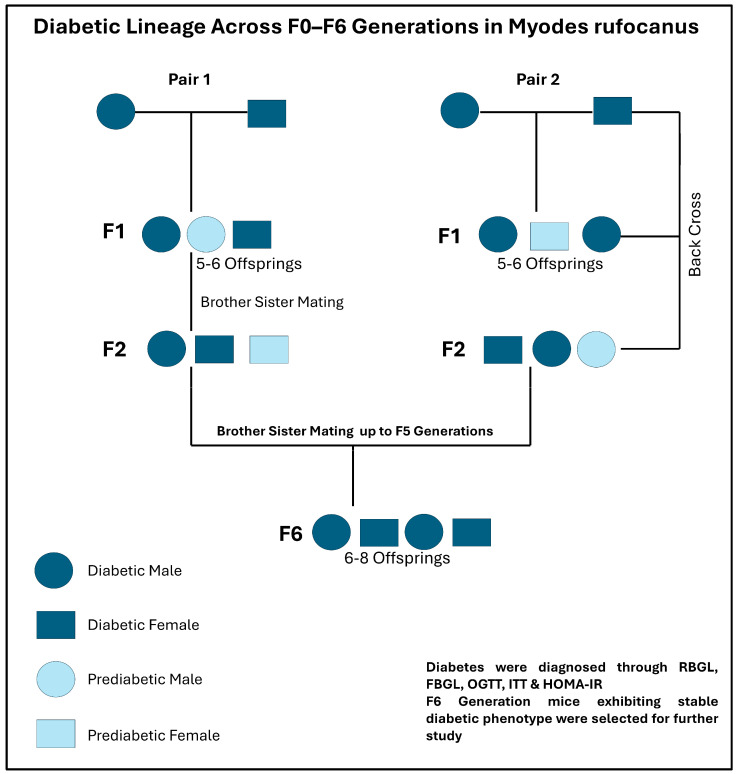
Stable diabetic phenotype established in *Myodes rufocanus* through selective breeding from F0–F6. Diabetes was assessed via RBGL, FBGL, OGTTs, ITTs, and HOMA–IR. Diabetic (dark blue, glucose level exceeding 11.1 mmol/L) and prediabetic (light blue, glucose level exceeding 7 mmol/L) males and females are shown. Brother–sister mating was conducted from F1–F5, and F6 voles with a stable diabetic phenotype were used for subsequent experiments.

To investigate the inheritance and stabilization of spontaneous diabetes, a cohort of 237 *Myodes rufocanus* voles (10–30 weeks of age, both sexes) was initially screened for hyperglycemia using an ACCU-CHEK glucometer. Voles with blood glucose levels above 11.1 mmol/L, as determined by random and fasting measurements, were classified as diabetic, and five pairs were selected for a two-week observation period. The vole underwent oral glucose tolerance tests (OGTTs) and insulin tolerance tests (ITTs) to assess glucose regulation and insulin sensitivity. Based on these data, a pedigree spanning six generations (F0–F6) was established to trace the inheritance of the diabetic phenotype (Figure 1). Brother–sister mating from the F1 to F5 generations was employed to enhance trait stability. By the F6 generation, the male vole consistently exhibited a stable diabetic phenotype, and 18-week-old F6 males were selected for experimental analyses, providing a robust in vivo model of spontaneous type 2 diabetes. Age-matched normoglycemic males from the same colony served as controls (*n* = 6 per group). Eligible 18-week-old male F6 voles that met the predefined diagnostic criteria for diabetes or non-diabetic status were randomly selected from the breeding colony for experimental inclusion. No animals were selected based on disease severity. Investigators were blinded to group allocation during phenotypic assessments, biochemical analyses, and downstream molecular experiments. No animals or samples were excluded after group assignment. The sample size (*n* = 6 per group) was chosen based on previous studies using spontaneous diabetic rodent models and was considered sufficient to detect biologically relevant differences while minimizing animal use in accordance with the principles of the 3Rs.

### 2.2. Diabetes Induces Significant Physiological Alterations

**Figure 2:**
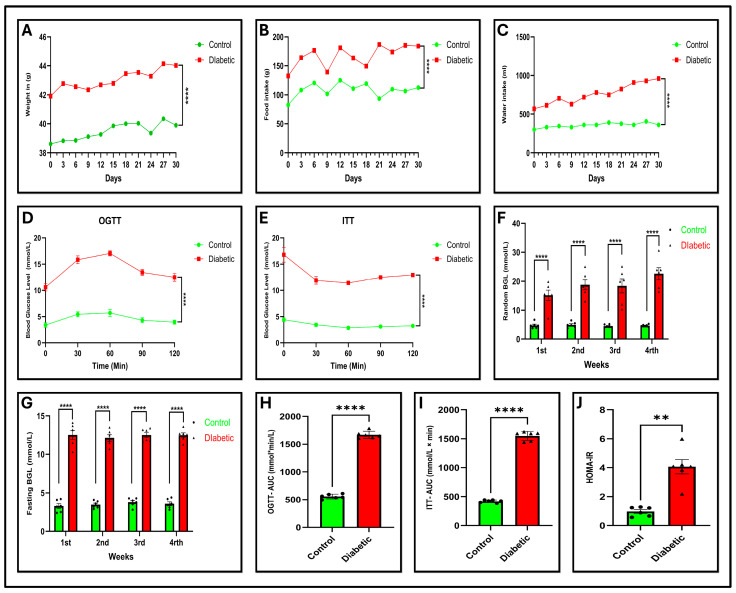
Diabetic voles, as a spontaneous T2DM model, exhibited elevated body weights, food intake, water consumption, random and fasting glucose concentrations, and impaired glucose and insulin tolerance compared with controls. (**A**) Body weight. (**B**) Food intake. (**C**) Water consumption. (**D**) Oral glucose tolerance test (OGTT): Glucose levels peaked at 30–60 min post-administration. (**E**) Insulin tolerance test (ITT): Sustained hyperglycemia observed at 60 min post-injection. (**F**) Random blood glucose levels. (**G**) Fasting blood glucose exceeding 11.1 mmol/L. (**H**) OGTT area under the curve (AUC). (**I**) ITT AUC. (**J**) Homeostatic model assessment for insulin resistance (HOMA-IR). Data are presented as the mean ± SD for panels (**D**,**E**,**H**,**I**) and the mean ± SEM for panels (**A**–**C**,**F**,**G**,**J**) (*n* = 6 animals per group for all panels). Asterisks denote statistical significance (** *p* < 0.01 and **** *p* < 0.0001), as determined by a two-tailed unpaired *t*-test with Welch’s correction (**A**–**E**,**H**–**J**) and ordinary two-way ANOVA (**F**,**G**).

Control and diabetic voles were fed a conventional chow diet. Body mass, as well as water and food consumption, were systematically recorded every three days over a span of four weeks. As the diabetic vole represents a spontaneous T2DM model, baseline differences in these parameters were expected. Compared to the control group, diabetic voles demonstrated a statistically marked elevation (*p* < 0.0001) in body weight, water consumption, and food intake, as shown in Figure 2A–C respectively.

### 2.3. Diabetes Leads to Elevation in Blood Glucose Level

The random and FBGL levels of the voles in each experimental group were evaluated weekly for four weeks. The diabetic group showed significantly (*p* < 0.01) increased blood glucose levels (>11.1 mmol/L) compared to the control group, with a progressive increase over time, as illustrated in Figure 2F. After an eight-hour fasting period, the vole of the diabetic group demonstrated significantly (*p* < 0.01) higher blood glucose levels (>11.1 mmol/L) in comparison with the control group, as depicted in Figure 2G.

### 2.4. Diabetes Contributes to Impaired Glucose Tolerance and Insulin Resistance

OGTTs and ITTs are extensively employed and are recommended techniques for diagnosing diabetes and evaluating glucose regulation, offering vital insights into insulin sensitivity [21,22]. The OGTT findings indicated that in all groups, blood glucose levels reached their highest points 30 and 60 min after glucose administration, after which they began to decrease. As revealed in Figure 2D,H, there were substantial variations in the OGTT (*p* < 0.0001) and area under the curve (AUC) (*p* < 0.0001) between the control and diabetic voles. Upon insulin administration, both experimental groups demonstrated a substantial decline in glycemic levels during the initial 30 min. Subsequently, the control group showed a decline in blood sugar levels, with a slight increase as the experiment neared completion. Conversely, the diabetic cohort exhibited a gradual increase in blood glucose levels, which began 60 min after insulin injection. The ITT (*p* < 0.0001) and its AUC (*p* < 0.0001) highlighted significant deviations in insulin tolerance between the control and diabetic voles, as displayed in Figure 2E,I. The elevated glucose and insulin levels observed in diabetic voles during the OGTT and ITT suggested impaired glucose metabolism and reduced insulin sensitivity, respectively. The HOMA-IR data, as depicted in Figure 2J, demonstrated a marked increase in insulin resistance (*p* < 0.01) within the diabetic cohort compared with the control group.

### 2.5. Dysregulated Serum Insulin, Leptin, and FFAs in Diabetic Voles

**Figure 3:**
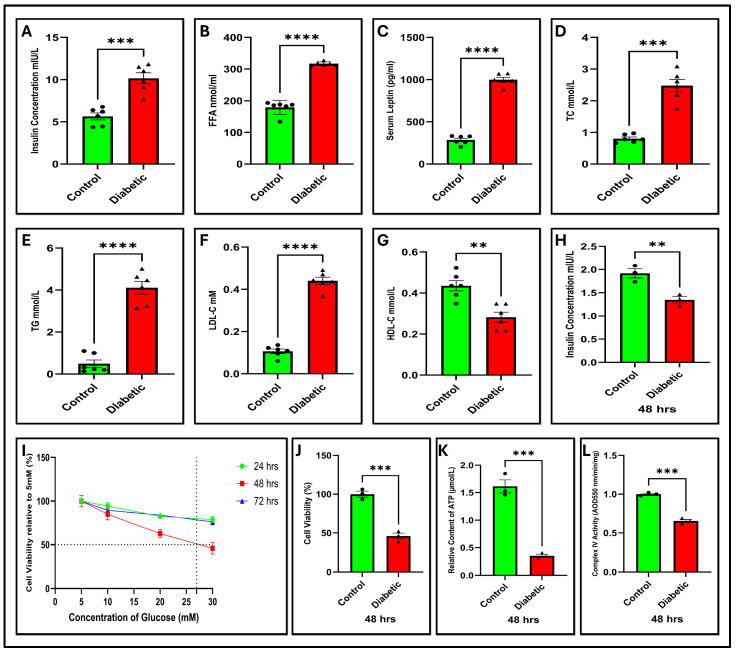
Diabetic voles exhibit elevated serum insulin, leptin, and free fatty acids, accompanied by dyslipidemia with increased TC, TG, and LDL-C and decreased HDL-C compared to controls. (**A**) Serum insulin. (**B**) Serum leptin. (**C**) Serum FFA. (**D**) Total cholesterol. (**E**) Triglycerides. (**F**) LDL-C. (**G**) HDL-C. (**H**) GSIS: reduced insulin secretion in MIN6 cells. (**I**,**J**) MIN6 cell viability decreased with 30 mM glucose at 24–72 h. (**K**,**L**) ATP content and Complex IV activity decreased in diabetic MIN6 cells. For in vivo serum analyses (**A**–**G**), *n* = 6 animals per group, with each sample measured in technical triplicate and values averaged. For in vitro experiments (**H**–**L**), data represent three independent experiments (*n* = 3), each performed in technical triplicate. Data are presented as the mean ± SEM. Asterisks denote statistical significance (** *p* < 0.01, *** *p* < 0.001, and **** *p* < 0.0001) determined by a two-tailed unpaired *t*-test with Welch’s correction (**A**–**H**,**J**–**L**) and two-way ANOVA with post hoc comparisons (**I**).

In this study, ELISA was applied to assess the serum insulin, leptin, and FFA levels in the two groups. Statistical analysis indicated significant differences in the levels of insulin (*p* < 0.001), FFAs (*p* < 0.0001), and leptin (*p* < 0.0001) between the diabetic vole and the control group, as shown in Figure 3A–C. These findings imply a reduction in insulin resistance, disruption of metabolic processes, and dysregulation of inflammatory responses, particularly those affecting insulin action and glucose homeostasis, which are characteristic features of type 2 diabetes.

### 2.6. Diabetic Voles Exhibit Altered Serum Lipid Profiles

The analysis of lipid profiles in serum samples has become an essential method for diagnosing and assessing the diabetes risk. In this investigation, we observed a significant elevation in the serum levels of TC (*p* < 0.001), TG (*p* < 0.0001), LDL-C (*p* = 0.0001), and HDL-C (*p* < 0.01) in diabetic voles compared to controls, as depicted in Figure 3D–G. Increased blood lipid levels in diabetic voles indicate metabolic imbalances that are often linked to reduced insulin sensitivity, higher levels of blood glucose, and a greater likelihood of acquiring diabetes-related disorders in humans.

### 2.7. Transcriptomic Profiling Reveals Gene Expression Changes in Diabetic Pancreas

**Figure 4:**
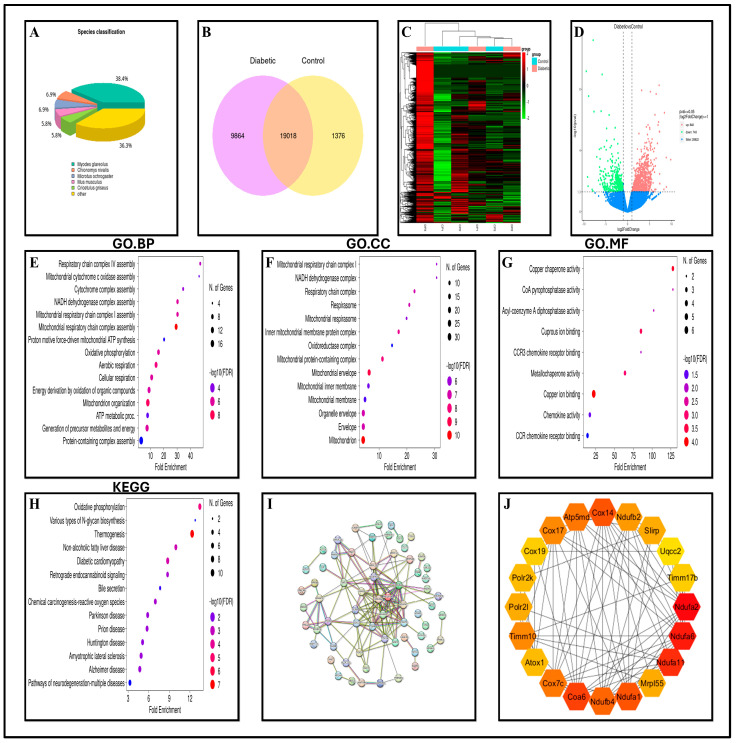
Transcriptomic analysis of diabetic versus control voles reveals downregulated pathways and identifies COX14 and related mitochondrial genes as potential regulators of diabetes pathogenesis. (**A**) Species classification showing 38.4% similarity with *Myodes glareolus*. (**B**) Venn diagram of gene expression: 19,018 shared, 9864 diabetic-specific, and 1376 control–specific. (**C**) Heatmap of gene expression patterns. (**D**) Volcano plot showing 1591 DEGs (848 upregulated and 743 downregulated). (**E**) Top 15 downregulated biological processes. (**F**) Top 15 downregulated cellular components. (**G**) Top 10 downregulated molecular functions. (**H**) Top 10 downregulated KEGG pathways. (**I**) PPI network of downregulated genes. (**J**) Hub genes linked to mitochondrial dysfunction, β–cell impairment, and diabetes pathogenesis.

RNA-seq reads for each sample were sequenced to an average depth of approximately 22 million paired-end reads per sample. The raw reads underwent quality assessment using FastQC and were processed with Fastp, and transcript abundance was quantified using RSEM. Differential expression analysis was performed using DESeq2 while employing median-of-ratios normalization. Significance was determined by a |log_2_ fold change| of at least one and an adjusted *p*-value of 0.05 or less using the Benjamini–Hochberg correction procedure. FPKM values were used solely for visualization and clustering. Clean reads obtained from the raw sequencing data were processed using FASTQC and FASTP to enhance the precision of de novo assembly. The assembly was constructed using the Trinity and Unigene software (Trinity v2.8.5). The analysis of Trinity results revealed that most transcripts were either in the range of 301–500 base pairs or exceeded 2000 bp. In the species classification analysis, homologous sequences were predominantly associated with *Myodes glareolus* (38.4%) and *Chionomys nivalis* (36.3%), with smaller proportions linked to *Microtus ochrogaster* (6.9%), *Mus musculus* (5.8%), and *Cricetulus griseus* (5.8%). The remaining sequences were classified as belonging to other species, as shown in Figure 4A.

#### 2.7.1. Differential Gene Expression Highlights Dysregulated Genes in Diabetic Pancreas

A Venn diagram provides a visual representation of the overlap and distinctiveness of expressed genes: A total of 19,018 genes were common to both groups, while 9864 genes were specific to the diabetic samples, and 1376 genes were unique to the control group. The distribution is shown in Figure 4B (identification of potential targets for differential expression analysis). Gene expression in diabetic samples demonstrated significant alterations compared with control samples. Several genes that were upregulated (red) in the diabetic samples were downregulated (green) in the controls, and vice versa. Hierarchical clustering using heat maps effectively distinguished the diabetic samples from the controls, highlighting distinct gene expression profiles. This differentiation is illustrated in Figure 4C, thereby emphasizing the systematic influence of diabetes on gene regulation.

#### 2.7.2. Identification of Differentially Expressed Genes (DEGs)

DGE analysis was conducted using DESeq2 to compare diabetic subjects with control groups. This analysis identified 1591 DEGs, including 848 upregulated and 743 downregulated genes. The most upregulated genes were IGH, IL21R, ITGA4, TOP2, RSKL, WDFY4, CD4, BCL11B, RUBCNL, and TRAF1, whereas the most downregulated genes were REG3, REG1, CFAP57, CLDN, CA, TTLL9, KRAB, GCGR, TEX15, and H2B (Table S1). These genes indicate significant molecular pathways that distinguish patients with diabetes from healthy controls. The volcano plot is shown in Figure 4D (visual representation of distinct clusters of upregulated (red) and downregulated (green) genes, with nonsignificant genes (blue) appearing near the zero line). This analysis underscores the common transcriptional shifts in diabetic samples compared with controls, providing a foundation for more detailed pairwise analyses in the future.

#### 2.7.3. Functional Enrichment Reveals Altered Pathways in Diabetic Pancreas

The GO analysis explored the biological processes (BPs), cellular components (CCs), and molecular functions (MFs) affected by genes that were downregulated in diabetic samples relative to control samples. As depicted in Figure 4E, enriched BP revealed a substantial link between these genes and the regulation of cellular bioenergetic homeostasis. These processes encompass the assembly of respiratory chain complexes including mitochondrial cytochrome c oxidase and NADH dehydrogenase. Other enriched processes included mitochondrial ATP synthesis associated with proton transport, oxidative phosphorylation, aerobic and cellular respiration, energy production through the oxidation of organic compounds, mitochondrial organization, and ATP metabolic processes. The enriched CCs are shown in Figure 4F, highlighting the essential role of mitochondrial structural integrity and compartmentalization in the preservation of cellular homeostasis. These components include respiratory chain complexes, respirasomes, mitochondrial protein-containing complexes, and various mitochondrial and organelle envelope structures. The key enriched MFs are illustrated in Figure 4G, underscoring the involvement of DEGs in the pathways linked to oxidative stress, mitochondrial dysfunction, lipid metabolism disruption, and chronic inflammation. These functions include ion binding, chaperone activity, enzymatic processes, and chemokine-related functions.

KEGG pathway enrichment analysis identified significantly enriched pathways associated with downregulated DEGs in diabetic samples compared with control samples. These pathways are shown in Figure 4H and include metabolic processes, such as Oxidative Phosphorylation and N-Glycan Biosynthesis, along with disease-related pathways, including Non-Alcoholic Fatty Liver Disease, Diabetic Cardiomyopathy, and various neurodegenerative disorders.

#### 2.7.4. Protein–Protein Interaction and Hub Gene Identification

The analysis of downregulated genes was conducted using STRING to identify protein–protein interactions, as shown in Figure 4I. This analysis revealed extensive networks in both the categories, as shown in the figures. The STRING results were further examined using CytoHubba, a Cytoscape (v 3.10.4) plugin, to determine the top 20 hub genes: NDUFA2, NDUFA6, NDUFA11, COA6, COX14, NDUFA1, NDUFB4, ATP5MD, COX7C, TIMM10, COX17, NDUFB2, MRPL55, POLR2L, SLIRP, ATOX1, POLR2K, COX19, TIMM17B, and MRPS36. Their interaction scores were 20, 19, 15, 14, 13, 13, 12, 11, 11, 10, 10, 9, 8, 8, 8, 7, 7, 6, 6, and 5, respectively, as illustrated in Figure 4J. Detailed information on the ranks and scores of the downregulated hub genes is provided in Table S2.

#### 2.7.5. Key Hub Genes Identified for Downstream Analysis

Mitochondrial dysfunction and impaired oxidative phosphorylation in pancreatic beta cells lead to reduced ATP production and insulin secretion, both of which are crucial for the pathogenesis of diabetes [23,24]. A comprehensive literature review identified several genes linked to mitochondrial malfunction and defective oxidative phosphorylation that were downregulated. The novel hub gene COX14 was selected for this study because of its significant role in oxidative phosphorylation and ATP production to investigate its involvement in the pathogenesis of diabetes. Table 1 presents the expression level of this gene, along with its respective rank and score in the diabetic group versus controls.

Because RNA sequencing was performed on whole pancreatic tissue, which comprises both endocrine and exocrine compartments as well as resident and infiltrating immune cells, the observed differential gene expression may reflect changes in cellular composition in addition to transcriptional regulation within specific cell types. Therefore, the identified differentially expressed genes are interpreted as being associated with the diabetic pancreatic state rather than representing definitive β-cell–intrinsic regulatory mechanisms.

### 2.8. Validation of COX14 Expression via RT-qPCR and Western Blots

**Figure 5:**
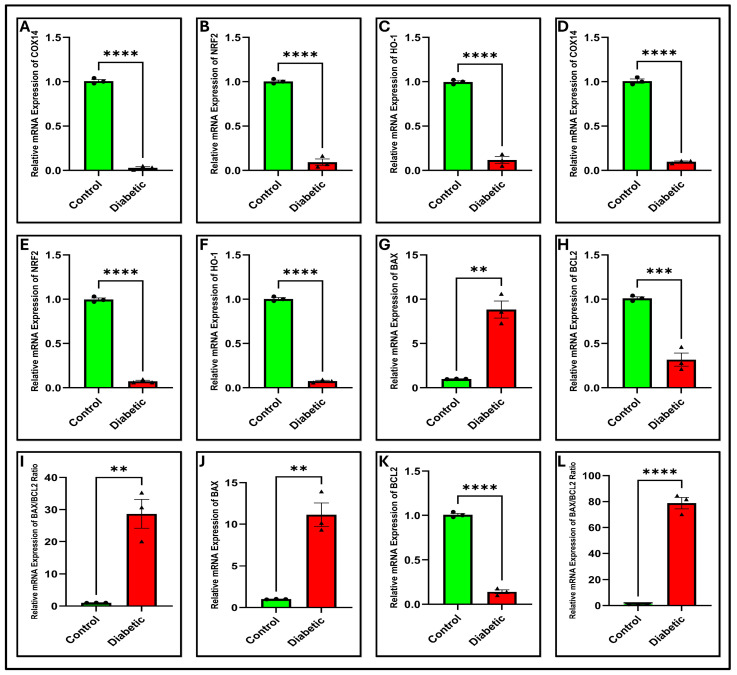
Altered mRNA expression of COX14, NRF2, HO-1, BAX, and BCL2 in pancreatic tissue and MIN6 cells from diabetic and control voles. (**A**–**C**) mRNA levels of COX14, NRF2, and HO-1 in pancreatic tissue. (**D**–**F**) mRNA levels of COX14, NRF2, and HO-1 in MIN6 cells. (**G**–**I**) mRNA expression of BAX and BCL2 and the BAX/BCL2 ratio in pancreatic tissue. (**J**–**L**) mRNA expression of BAX and BCL2 and the BAX/BCL2 ratio in MIN6 cells. RT-qPCR was performed using β-actin as the housekeeping gene, with mRNA levels normalized to controls, consistent with RNA-seq results. For pancreatic tissue analyses (**A**–**C**,**G**–**I**), *n* = 3 animals per group, with each sample measured in technical triplicate and values averaged. For in vitro MIN6 cell experiments (**D**–**F**,**J**–**L**), data represent three independent experiments (*n* = 3), each performed in technical triplicate. Data are presented as the mean ± SEM. Asterisks denote statistical significance (** *p* < 0.01, *** *p* < 0.001, and **** *p* < 0.0001), as determined by a two-tailed unpaired *t*-test with Welch’s correction.

RNA-seq analysis identified COX14 as a hub gene that was significantly downregulated in diabetic pancreatic tissues. To validate this finding, we assessed COX14 expression by qPCR and Western blotting in both pancreatic tissues and MIN6 cells. These findings demonstrated a substantial decrease in COX14 mRNA and protein levels in diabetic samples compared to those in controls. Quantitative reverse transcription PCR (RT-qPCR) analysis demonstrated a considerable decrease in COX14 mRNA abundance in the diabetic group compared with that in the control group (Figure 5A; *p* < 0.0001 and (D) *p* < 0.0001) in pancreatic tissue and MIN6 cells, respectively.

**Figure 6:**
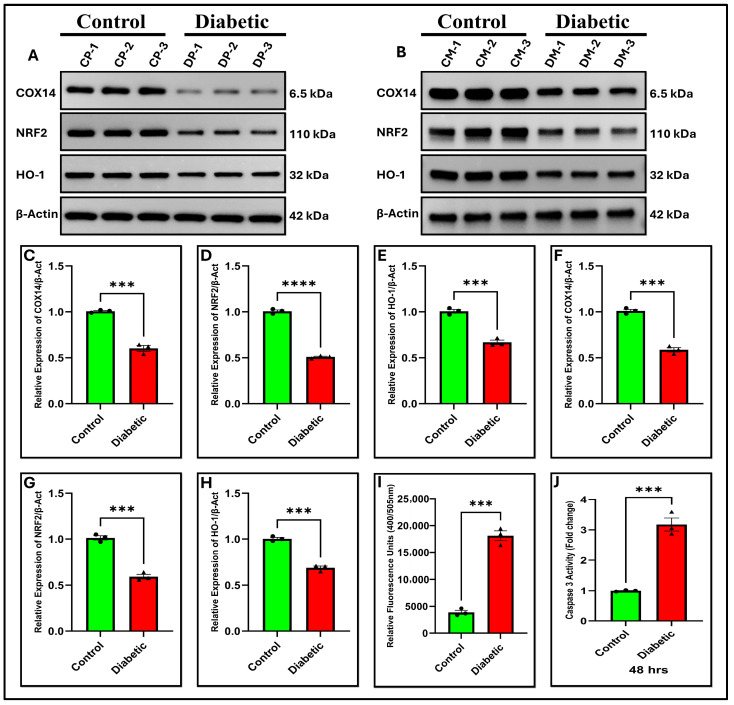
Western blot analysis of COX14, NRF2, HO-1, and caspase-3 activity in pancreatic tissue and MIN6 cells from the diabetic and control groups. (**A**) Representative Western blot of COX14, NRF2, and HO-1 in pancreatic tissue (CP: control pancreas; DP: diabetic pancreas). (**B**) Representative Western blot of COX14, NRF2, and HO-1 in MIN6 cells (CM: control MIN6; DM: diabetic MIN6). (**C**–**E**) Quantification of COX14, NRF2, and HO-1 protein expression in pancreatic tissue. (**F**–**H**) Quantification of COX14, NRF2, and HO-1 protein expression in MIN6 cells. (**I**) Relative caspase-3 activity in pancreatic tissue after a 48 h treatment period. (**J**) Relative caspase-3 activity in MIN6 cells after 48 h of treatment. Protein expression and caspase-3 activity were normalized to control values. For pancreatic tissue analyses (**A**,**C**–**E**,**I**), *n* = 3 animals per group, with each sample measured in technical triplicate and values averaged. For in vitro MIN6 cell experiments (**B**,**F**–**H**,**J**), data represent three independent experiments (*n* = 3), each performed in technical triplicate. Data are presented as the mean ± SEM. Asterisks denote statistical significance (*** *p* < 0.001 and **** *p* < 0.0001), as determined by a two-tailed unpaired *t*-test with Welch’s correction.

This transcriptional change was reflected in protein abundance. Western blotting revealed a substantial decrease in COX14 protein expression (Figure 6A,B) and a quantitative change (Figure 6C, *p* < 0.001 and Figure 6F, *p* < 0.001) in the pancreatic tissues and MIN6 cells of the diabetic group, respectively. The present results substantiate the RNA sequencing data and confirm that COX14 is differentially expressed in our model.

### 2.9. Diabetes Impairs Mitochondrial Function and Bioenergetics

**Figure 7:**
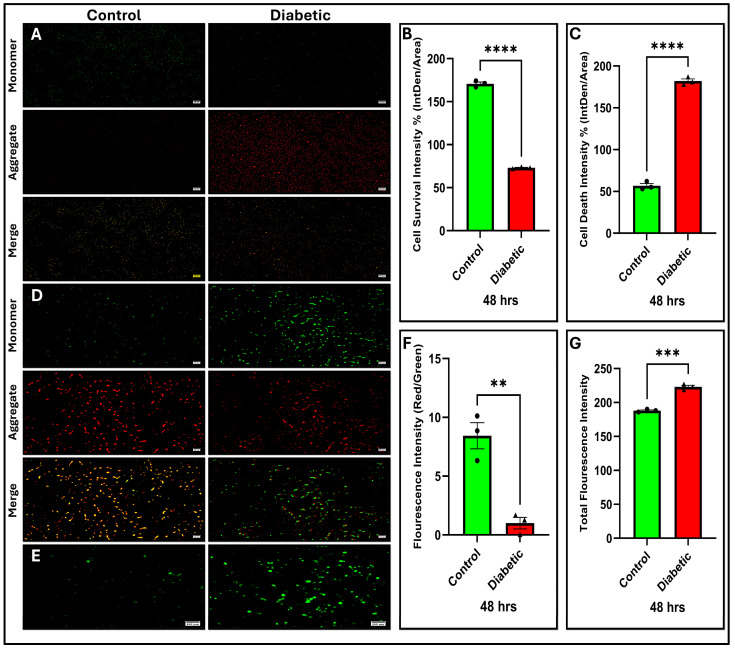
Diabetes induces functional impairments in MIN6 cells, including reduced viability, mitochondrial dysfunction, and increased ROS in diabetic versus control MIN6 cells over a 48 h period. (**A**) Calcein-AM/PI assay images were used to assess (**B**) cell viability and (**C**) cell death intensity, with significant differences observed in the diabetic group (**** *p* < 0.0001) compared with the control. (**D**) JC-1 images demonstrate variations in the MMP, as represented by (**F**) fluorescence intensity (** *p* < 0.01) in both groups. (**E**) A DCFH-DA fluorescent probe was utilized to quantify ROS production, which is depicted as (**G**) total fluorescence intensity (*** *p* < 0.001) in the diabetic group relative to the control. All measurements were obtained from three independent experiments (*n* = 3), with each assay performed in triplicate and values normalized to controls. Results are expressed as the mean ± SEM. Microscopic images are shown with scale bars of 50 µm and 100 µm. Statistical significance between groups was assessed using a two-tailed unpaired *t*-test with Welch’s correction.

COX14 plays a critical role in cytochrome c oxidase assembly (Complex IV), and its downregulation may be associated with a reduction in the enzymatic activity of Complex IV, as shown in (Figure 3L; *p* < 0.001), thereby impairing electron transport efficiency. This impairment may be associated with a marked decline in cellular ATP levels (Figure 3K; *p* < 0.001). Consistent with these observations, JC-1 staining revealed a substantial collapse in the mitochondrial membrane potential (ΔΨm), as demonstrated by the JC-1 assay in Figure 7D and the corresponding fluorescence ratios (Figure 7F, *p* < 0.01), indicating mitochondrial depolarization. Collectively, these results suggest that reduced COX14 expression may likely disrupt oxidative phosphorylation and contribute to energy deficiency in β-cells under diabetic conditions.

### 2.10. Diabetes Induces ROS Accumulation and Suppresses the NRF2 Pathway

In alignment with respiratory chain dysfunction, ROS levels, as assessed by DCFH-DA, were significantly elevated in diabetic MIN6 cells, as illustrated in (Figure 7E,G; *p* < 0.001). Considering the established role of the NRF2 pathway in cellular stress responses and its potential association with mitochondrial regulators, we postulated that the downregulation of COX14 might influence NRF2 signaling. Notably, both quantitative PCR (qPCR) and Western blot analyses demonstrated a decrease in the levels of NRF2 and HO-1 in the pancreas and in MIN6 cells. The qPCR results demonstrated a substantial decrease in the levels of NRF2 and HO-1 transcripts in the diabetic group compared to the control group (Figure 5B,C; *p* < 0.0001) and (Figure 5E,F; *p* < 0.0001) in pancreatic tissue and MIN6 cells, respectively. Similarly, Western blotting confirmed that the protein abundances of NRF2 and HO-1 were markedly diminished, as depicted in Figure 6A,B and subsequent quantifications (Figure 6D,E; *p* < 0.0001; *p* < 0.001), (Figure 6G,H; *p* < 0.001) in pancreatic tissue and MIN6 cells, respectively. These findings indicate that reduced COX14 expression may be associated with enhanced oxidative stress and impairment of the antioxidant defense system.

### 2.11. Diabetes Modulates Apoptotic Regulators and Activates Caspase-3

Since NRF2 suppression can sensitize cells to apoptosis, we next investigated the crucial markers of the intrinsic apoptotic pathway. We assessed the mRNA expression of Bax, a pro-apoptotic protein, and Bcl-2, an anti-apoptotic protein. The ratio of *Bax*/Bcl-2, a critical indicator of apoptotic commitment, significantly increased in the pancreatic tissue and MIN6 cells (*p* < 0.01 and *p* < 0.0001, respectively, Figure 5I,L) of the diabetic group. This shift was driven by a significant surge in the mRNA levels of *Bax* (*p* < 0.01 and *p* < 0.01, Figure 5G,J) and a significant decrease in the mRNA levels of Bcl-2 (*p* < 0.001 and *p* < 0.0001, Figure 5H,K) in pancreatic tissues and MIN6 cells, respectively.

As BAX/BCL2 imbalance can trigger the caspase cascade, we tested caspase-3 activity. Caspase-3 activity was evaluated using fluorometric analysis to assess caspase activation in the downstream executioner. In the diabetic cohort, caspase-3 activity in the pancreatic tissue and MIN6 cells were markedly elevated relative to that in controls (*p* < 0.001 and *p* < 0.001, respectively; Figure 6I,J), confirming enhanced apoptotic signaling. These findings suggest that COX14 downregulation may be associated with mitochondrial dysfunction, oxidative stress, and apoptosis in diabetes.

### 2.12. Diabetes Impairs β-Cell Viability and Insulin Secretion

Functional viability assays further supported these findings. Diabetic MIN6 cells demonstrated a decline in viability, as assessed by the CCK-8 assay (Figure 3I,J; *p* < 0.001), and the Calcein-AM/PI assay (Figure 7A) showed a notable decrease in cell survival and a rise in cell death among diabetic cells, as indicated by the substantial decrease in viable cells (Figure 7B) and elevated PI intensity (Figure 7C; *p* < 0.0001).

Moreover, GSIS was significantly reduced (Figure 3H; *p* < 0.001) in MIN6 cells after 48 h. These findings suggest that mitochondrial dysfunction and oxidative stress not only reduce β-cell viability but also contribute to the gradual deterioration of β-cells, eventually impairing insulin secretion.

The integration of in vivo and in vitro data suggests a potential mechanistic pathway wherein the downregulation of COX14 is associated with impaired Complex IV activity and disrupted mitochondrial energy dynamics. This alteration may contribute to excessive ROS generation and a diminished NRF2/HO-1 antioxidant response. The ensuing oxidative stress could activate the intrinsic apoptotic pathway, as reflected by changes in the BAX/BCL2 ratio and increased caspase-3 activity, ultimately compromising β-cell viability and insulin secretion. Collectively, these findings indicate that COX14 may be associated with mitochondrial integrity and β-cell stability in the diabetic state; however, its precise mechanistic contribution remains to be fully elucidated.

## 3. Discussion

Diabetes mellitus (DM) is a chronic metabolic condition marked by high blood glucose levels and is experiencing an increase in prevalence worldwide. This condition is associated with increased morbidity and mortality rates owing to complications. It encompasses major vascular issues, such as heart disease, kidney failure, stroke, and microvascular complications affecting the eyes, kidneys, and peripheral nerves. The escalating incidence of diabetes and its health implications pose a significant challenge to healthcare systems worldwide and profoundly impact patient outcomes [25,26]. The intricate nature of diabetes mellitus (DM) involves numerous factors and molecular pathways. Researchers have developed various animal models for diabetes research, with a primary focus on rodents, including transgenic and non-obese diabetic voles; biobreeding diabetes-prone rats; and models induced by chemicals like streptozotocin or high-fat diets [27,28]. Spontaneous diabetes models such as non-obese diabetic (NOD) mice closely replicate human diabetes pathology, rendering them crucial for investigating disease mechanisms and potential therapies [29]. In this context, *Myodes rufocanus* voles have attracted scientific interest owing to their high incidence of spontaneous diabetes, with rates reported to reach up to 43% [18]. Notably, diabetes in *Myodes rufocanus* occurs naturally and is perpetuated through selective breeding, without the use of genetic modification or chemical induction, setting it apart from traditional rodent models. This distinct characteristic makes *Myodes rufocanus* especially valuable for investigating naturally occurring metabolic imbalances and mitochondrial dysfunction related to type 2 diabetes. However, despite this benefit, the molecular mechanisms driving diabetes development in this species are still largely unexamined.

This species exhibits a high prevalence of spontaneous diabetes under controlled laboratory conditions, making it a valuable non-induced model for studying diabetes pathogenesis and mitochondrial dysfunction. Animals were maintained under standardized husbandry conditions appropriate for vole species. This study provides compelling evidence that *Myodes rufocanus* is a rodent species that naturally develops type 2 diabetes, as confirmed through comprehensive metabolic and molecular analyses. We identified the mitochondrial assembly factor COX14 as a potentially important component for maintaining β-cell mitochondrial integrity, antioxidant capacity, and insulin secretion. Our findings suggest a putative pathophysiological sequence in which reduced COX14 expression is associated with impaired oxidative phosphorylation, redox imbalance, apoptotic β-cell loss, and insulin secretion failure. This study not only establishes a novel model but also offers valuable insights into the pathogenesis of type 2 diabetes.

In this study, *Myodes rufocanus* displayed the typical manifestations of type 2 diabetes, such as progressive weight gain, continuous hyperphagia, and polydipsia. These primary symptoms, including increased body weight and elevated intake of food and water, were similarly noted in C57BL/6J mice that were maintained on a high-fat diet for a duration of four weeks, as documented by Thuy Nguyen-Phuong et al., 2023 [30]. Moreover, *Myodes rufocanus* exhibited elevated fasting and random blood glucose levels. Similarly, C57BL/6 mice maintained on a high-fat diet for 21 weeks demonstrated significant elevations in fasting and postprandial blood glucose concentrations [31]. These metabolic disturbances were accompanied by a notable decline in glucose tolerance and significant insulin resistance, as demonstrated by oral glucose and insulin tolerance tests and increased HOMA-IR in our diabetic model. Comparable impairments in glucose tolerance and insulin responsiveness were likewise detected in male C57BL/6NCr mice (NCI Frederick) and mice exposed to high-fat diets and STZ injections, along with a marked increase in HOMA-IR in HFD rats compared to CD rats [32,33]. HOMA-IR is a reliable marker of insulin resistance in the clinical progression of type 2 diabetes [34].

Studies using diabetic rodent models have revealed notable disruptions in lipid metabolism linked to T2DM. In ApoA-II transgenic mice, there is a pronounced increase in free fatty acids, adipose tissue, and plasma leptin and insulin levels, all of which correlate with insulin resistance [35]. Moreover, the characteristic dyslipidemia of type 2 diabetes, marked by elevated total cholesterol and triglycerides, either increased or normal LDL, and decreased HDL, has been effectively reproduced in both ApoA-II transgenic and knockout mice. These models not only mirror the insulin resistance and lipid irregularities observed in human patients but also underscore the significance of ApoA-II in lipid metabolism regulation, providing mechanistic insights into lipid metabolism disruptions linked to diabetes [36,37]. Similarly, *Myodes rufocanus* exhibited altered lipid metabolism, with heightened levels of serum insulin, free fatty acids, leptin, triglycerides, total cholesterol, LDL-C, and HDL-C, all occurring in conjunction with reduced insulin secretion capacity. Notably, these features developed without any dietary or pharmacological intervention, establishing *Myodes rufocanus* as a unique, naturally occurring model of T2DM that encapsulates the intricate interplay between weight gain, insulin resistance, lipotoxicity, and dysfunction of β-cells. Circulating levels of soluble receptors for advanced glycation end products (sRAGEs) exhibit significant variability across diseases and their respective stages. In the spontaneous diabetes model of *Myodes rufocanus*, alterations in sRAGE levels are more likely to reflect the ongoing activation of the advanced glycation end-product (AGE)–RAGE axis and sustained metabolic stress rather than a transient compensatory response.

In the current study, the investigation of hub genes was confined to those genes that were markedly downregulated, as oxidative phosphorylation was identified as the most significantly suppressed pathway in the diabetic pancreas. The CytoHubba DEGREE metric was applied to identify highly interconnected nodes within this network, with a specific focus on mitochondrial genes that may be integral to metabolic dysfunction. Hub genes that were upregulated, many of which are acknowledged as principal inflammatory regulators in diabetes, were not further prioritized to underscore novel mitochondrial gene candidates. Consequently, the identified hub genes are candidates that focus on distinct oxidative phosphorylation pathways. In this study, transcriptomic analysis of pancreatic tissues identified COX14 as a significantly downregulated hub gene. This finding was substantiated by validation experiments, which demonstrated a marked reduction in COX14 mRNA and protein expression within diabetic pancreatic tissues and MIN6 β-cells. Known as C12orf62 in humans, COX14 is a mitochondrial transmembrane protein within the MITRAC complex and is essential for coordinating the coupling of COX1 translation to the early stages of Complex IV assembly [38,39]. Mitochondrial gene expression is critical for OXPHOS biogenesis, with COX14 playing a pivotal role in the synthesis of COX1, the central mitochondria-encoded subunit of Complex IV. In a CRISPR/Cas9-engineered COX14^M19I mouse model, the absence of COX14 resulted in Complex IV deficiency and impaired oxidative phosphorylation, underscoring its vital role in maintaining mitochondrial respiratory chain integrity [38]. Defective oxidative phosphorylation and mitochondrial dysfunction in pancreatic β-cells are central to the development of diabetes [40]. Similarly, transcriptional downregulation of mitochondrial Complex IV genes has been noted in T2DM, with restoration following rosiglitazone treatment [41]. Similarly, in individuals with type 2 diabetes, pancreatic islets displayed reduced expression of oxidative phosphorylation-related genes, including COX11, both in microarray data and at the mRNA level [42]. Mechanistic studies in yeast further demonstrated that deletion of COA3 or COX14 disrupts the coordination of COX1 synthesis with Complex IV assembly, negatively affecting COX1 protein expression and stability [43], highlighting the conserved role of COX14 in mitochondrial respiratory complex biogenesis. Loss of the COX4 subunit results in cytochrome c oxidase (Complex IV) deficiency due to impaired mitochondrial proteosynthesis, leading to impaired oxidative phosphorylation and mitochondrial dysfunction [44]. In obesity and diabetes, a high-fat diet has been associated with decreased expression of key mitochondrial oxidative phosphorylation genes, such as COX4I1 and COX5B, potentially resulting in mitochondrial dysfunction [45]. In human skeletal muscle, age-related changes in COX7A1 methylation and expression may promote insulin resistance and diabetes [46]. One study highlighted the conflicting expressions of COX7C predictive indices in diabetes-related sepsis. COX17 delivers copper to the COX2 subunit of Complex IV, which is essential for its catalytic activity [47]. COX17 deficiency impairs electron transport, increases oxidative stress, and has been linked to altered mitochondrial ultrastructure, reduced copper content, and defective cytochrome c oxidase biogenesis in HEK293 cells [48,49]. Collectively, these findings indicated that COX14 is one of the crucial regulators of Complex IV biogenesis. Its sustained downregulation, as demonstrated in our study at both the transcript and protein levels, is likely to disrupt the coupling of COX1 translation with complex assembly, impair electron transport, and exacerbate mitochondrial ROS production, thereby compromising β-cell function. Considering the coordinated dysregulation of multiple COX family members observed in diabetes and related metabolic disorders, COX14 may represent not only a key contributor to mitochondrial dysfunction in the diabetic state but also a mechanistically relevant candidate biomarker and a potential therapeutic target.

The downregulation of COX14 expression is associated with a marked reduction in Complex IV activity, compromised mitochondrial membrane potential, decreased ATP synthesis, and excessive accumulation of reactive oxygen species (ROS). These abnormalities underscore the indispensable putative role of COX14 in preserving mitochondrial respiratory chain function and maintaining bioenergetic balance in β-cells. Cytochrome c oxidase is essential for the regulation of mitochondrial membrane potential, and its malfunction can lead to potential disruption [50]. A significant decline in Complex IV enzyme activities has been documented in lymphocytes from individuals with T2DM, along with mitochondrial membrane depolarization and heightened oxidative damage [51]. Oxidative damage to mitochondrial membrane phospholipids, which undermines membrane integrity and function, contributes to the dysfunction of pancreatic β-cells in T2DM. This association appears to be modulated by obesity, as reductions in mitochondrial membrane potential and respiratory complex activities have been observed in obese diabetic patients but not in non-obese individuals with T2DM [52]. Mitochondrial dysfunction, characterized by impaired membrane potential, contributes to the development of insulin resistance in T2DM, thereby disrupting glucose homeostasis in liver and skeletal muscle tissues [53]. The current study observed a comparable loss of mitochondrial membrane potential in MIN6 cells after 48 h of 30 mM glucose treatment. Oxidative damage to mitochondrial membrane phospholipids, leading to compromised membrane integrity and function, contributes to failure of β-cells in T2DM [54].

Mitochondria play a vital role in bioenergetics and ATP production in numerous tissues, and any intrinsic disorder can significantly disrupt these processes [55]. In type 2 diabetes, mitochondrial dysfunction, particularly when involving Complex IV, results in diminished ATP production through several mechanisms. In diabetic patients, Complex IV activity is consistently lower in the skeletal muscle than in non-diabetic individuals [56]. Direct evidence indicates that mitochondria isolated from individuals with T2DM exhibit impaired ATP synthesis, with a decreased ATP synthesis ratio during hexokinase stimulation relative to baseline [57]. Magnetic resonance spectroscopy studies demonstrated a 30% reduction in mitochondrial ATP production in the insulin-resistant muscle of the offspring of T2DM individuals [58]. Impairment of ATP synthesis caused by mitochondrial dysfunction is correlated with the emergence of T2DM and the development of aging [55]. Notably, our current findings observed a similar trend of reduced ATP production in MIN6 cells after 48 h of treatment with 30 mM glucose compared to the controls. Defective oxidative phosphorylation, elevated expression of uncoupling protein 2 causing proton leakage, and diminished ATP synthesis in pancreatic β-cells lead to reduced insulin secretion and insulin resistance in the target tissues [59].

ROS is integral to the development of T2DM, primarily because of mitochondrial dysfunction and failure of β-cells. Persistent hyperglycemia under diabetic conditions elevates ROS production, disrupts β-cell function, and aggravates insulin resistance, contributing to the progression of T2DM [60]. Complex IV (cytochrome c oxidase) serves as the regulatory center for mitochondrial oxidative phosphorylation, and its dysfunction can lead to increased ROS production [61,62]. A deficiency in COX14 can cause Complex IV dysfunction and increase the ROS levels [38]. Consistent with this, the present research observed that COX14 downregulation was associated with excessive ROS accumulation due to mitochondrial dysfunction, as demonstrated in MIN6 cells subjected to 30 mM glucose for 48 h compared to the controls. ROS can damage electron transport complexes, including cytochrome c oxidase (Complex IV), creating a detrimental cycle in which oxidative damage disrupts electron transport, leading to increased electron leakage and further ROS generation [63]. This relationship is evident in cells lacking the Rieske iron–sulfur protein, where ROS-related reductions in Complex IV levels are observed, while exposure to ROS-generating compounds adversely affects Complex IV assembly and stability [64]. Overproduction of ROS induced by hyperglycemia in the electron transport chain contributes to oxidative stress and the worsening and sequelae of T2DM [65].

Nrf2 has been widely recognized as a central modulator of the antioxidant response, coordinating the production of proteins involved in both anti-inflammatory processes and ROS-scavenging enzymes [66,67,68]. However, it is crucial to recognize that while ROS production under normal physiological conditions typically triggers the Nrf2-mediated antioxidant response, diabetes is frequently associated with a weakened antioxidant defense system. This deficiency is often linked to mitochondrial dysfunction, which intensifies oxidative damage and leads to the gradual deterioration of β-cell function observed in diabetes [69,70]. For example, growing experimental evidence indicates that the activity of essential antioxidant response enzymes (such as HO-1, SOD, and GPx) is reduced in the vascular endothelial cells, plasma, retinas, and livers of individuals with diabetes [66]. Oxidative stress arises when ROS generation surpasses the neutralizing capability of the antioxidant system. Under hyperglycemic conditions, NF-κB and TGF-β are activated, producing self-sustaining pro-inflammatory and pro-oxidant signals that may surpass the antioxidant response. Furthermore, hyperglycemia causes glycation and functional loss of antioxidant enzymes, including SOD, which will be examined in connection with COX14 downregulation in future research [71,72]. Pretreatment with NFκB inhibitors partially mitigated oxidative stress induced by hyperglycemia, whereas AMPK inhibition further elevated ROS accumulation, underscoring the complementary roles of NFκB and AMPK in modulating antioxidant defense [73]. We know that the NRF2/HO-1 antioxidant pathway is critical for protecting against type 2 diabetes by mitigating cellular damage caused by oxidative stress. Empirical evidence suggests that polysaccharides exert antidiabetic effects by activating this pathway. For instance, okra polysaccharides alleviated diabetic symptoms in mice by initiating PI3K/AKT signaling, facilitating NRF2 translocation, and enhancing the expression of HO-1 and SOD2 [74]. Collectively, these findings suggest that inhibiting the NRF2/HO-1 pathway could exacerbate oxidative stress and lead to mitochondrial and β-cell dysfunction. Accumulating evidence indicates that mitochondrial dysfunction constitutes a critical pathogenic mechanism underlying oxidative stress in type 2 diabetes mellitus. The downregulation of COX14, a vital assembly factor for cytochrome c oxidase (Complex IV), is expected to diminish the efficiency of electron transport, potentially resulting in increased production of mitochondrial reactive oxygen species (ROS). Although acute elevations in ROS typically activate NRF2-dependent antioxidant signaling, chronic oxidative stress under prolonged diabetic conditions has been demonstrated to inhibit NRF2 activity and heme oxygenase-1 (HO-1) expression. Consequently, in this spontaneous diabetes model, the observed reduction in NRF2/HO-1 expression may reflect a compromised antioxidant defense system rather than the absence of an oxidative challenge. This observation is consistent with previous studies describing a self-sustaining cycle involving mitochondrial dysfunction, oxidative stress, and impaired redox signaling in chronic diabetes. Similarly, in our study, both the pancreatic tissue of diabetic voles and MIN6 cells exposed to hyperglycemic conditions exhibited significantly heightened oxidative stress, accompanied by downregulation of the Nrf2/HO-1 antioxidant defense pathway. Given the central role of the Nrf2/HO-1 axis in maintaining cellular redox homeostasis, its suppression likely results in excessive reactive oxygen species accumulation, thereby contributing to cellular dysfunction in diabetes [75,76].

In hyperglycemic conditions, mitochondria are damaged, alternative glucose pathways are activated, and non-enzymatic glucose reactions occur, leading to ROS accumulation and oxidative stress. This oxidative stress triggers the AGE–RAGE pathway, which intensifies mitochondrial dysfunction, supports alternative glucose metabolism, and contributes to insulin resistance, perpetuating elevated blood glucose levels. The intricate interactions among these pathways complicate the determination of the precise contributions of each pathway to overall oxidative stress. Furthermore, the specific effects on tissue-specific damage remain a significant yet unresolved issue [66]. The combination of elevated ROS levels and a diminished antioxidant response create a highly pro-oxidative environment within the cells, which is particularly detrimental to β-cells because of their inherently low antioxidant capacity.

Apoptosis is a highly regulated form of programmed cell death that can be triggered by both internal and external signals, and it is controlled by numerous genes [77]. Evidence suggests that reactive oxygen species (ROS) can activate mitochondria through intrinsic signaling pathways, thereby initiating the mitochondrial apoptotic pathway [78,79]. Proteins of the Bcl-2 family, including the pro-apoptotic protein Bax and the anti-apoptotic protein Bcl-2, play a crucial role in regulating this process [80,81]. Under physiological conditions, Bax interacts with Bcl-2 and related proteins to maintain steady protein levels [82]. When the mitochondrial apoptotic pathway is activated, Bax changes its conformation and moves from the cytoplasm to the outer mitochondrial membrane [83]. This movement triggers the opening of the mitochondrial permeability transition pore (MPTP), resulting in mitochondrial membrane depolarization and increased membrane permeability [84,85]. Subsequently, cytochrome c (Cyt-c), which is normally retained within the mitochondria, is released into the cytoplasm, where it binds apoptotic protease-activating factor-1 (Apaf-1), leading to the activation of caspase-9. This, in turn, activates the effector enzyme caspase-3, driving the cell toward apoptosis [86,87]. In the present study, high-glucose treatment significantly decreased MIN6 cell viability, as assessed by CCK-8 and Calcein-AM/PI assays. Moreover, the evaluation of key apoptotic markers showed an increased Bax/Bcl-2 ratio and elevated caspase-3 activity in both pancreatic tissue and MIN6 cells under high-glucose conditions. These results indicate that COX14 downregulation is associated with mitochondrial apoptotic activation, highlighting its potential involvement in β-cell dysfunction and apoptosis in diabetes.

Defective GSIS functions as a key indicator of β-cell dysfunction and is closely correlated with mitochondrial dysfunction, which is a fundamental cause of secretory defects observed in type 2 diabetes [88]. Several factors result in the mitochondrial malfunction of β-cells, collectively impeding insulin secretion [89]. In diabetic mouse models, mitochondrial abnormalities such as impaired membrane potential hyperpolarization, reduced calcium uptake, altered morphology, and diminished oxidative phosphorylation emerge before the onset of overt diabetes and are linked to impaired GSIS [90]. A central aspect of pancreatic β-cell dysfunction is the increase in apoptosis of β-cells, coupled with a decrease in the mass of β-cells. Depletion of β-cells via programmed cell death disrupts insulin signaling, exacerbates cellular dysfunction, and leads to a decline in insulin secretory capacity [91]. Studies have demonstrated a decline in GSIS in hPSC-derived β cells, contributing to disease onset [92]. Similarly, in this study, high-glucose conditions in MIN6 cells resulted in a significant impairment of GSIS, highlighting a direct connection between mitochondrial dysfunction and β-cell secretory defects characteristic of type 2 diabetes.

Ultimately, impaired oxidative phosphorylation and mitochondrial dysfunction in pancreatic β-cells play crucial roles in diabetes development. Furthermore, studies have shown that oxidative stress caused by glucolipotoxicity in pancreatic β-cells leads to mitochondrial malfunction, resulting in decreased insulin production and cell death during progression [93]. Mitochondrial anomalies also contribute to insulin resistance, whereas persistent hyperglycemia and hyperlipidemia exacerbate mitochondrial impairment by boosting the production of ROS [93,94]. Consequently, oxidative stress and ROS accumulation are key factors in mitochondrial impairment, leading to reduced ATP synthesis, compromised insulin secretion, and eventual β-cell failure, eventually leading to DM development and progression [95].

This study highlights the following three significant aspects: First, we introduced *Myodes rufocanus* as a natural rodent model for type 2 diabetes, eliminating the need for artificial induction and accurately reflecting the inherent development of the disease. Second, we revealed a crucial yet previously overlooked association of COX14 with β-cell mitochondrial function and survival. Third, our findings indicate that the concurrent decline of mitochondrial oxidative phosphorylation and Nrf2/HO-1 antioxidant defense marks a pivotal stage in β-cell deterioration under metabolic stress. From a therapeutic perspective, these observations imply that maintaining COX14 expression could support Complex IV integrity and mitochondrial stability, while pharmacological activation of Nrf2/HO-1 might mitigate oxidative damage and preserve β-cell mass and function. Notably, assessing COX14 expression in human diabetic islets could clarify its clinical relevance and identify potential biomarkers or therapeutic targets.

In summary, in contrast to chemically induced or monogenic rodent models, which rely on acute β-cell injury or predefined genetic alterations, the *Myodes rufocanus* model develops type 2 diabetes spontaneously and progressively without external intervention (Table S4). This characteristic closely reflects the chronic multifactorial nature of T2DM in humans. Within this context, mitochondrial dysfunction and oxidative stress appear to be intrinsic features of disease progression rather than secondary consequences of experimental manipulation. The observed reduction in COX14 expression, together with altered redox homeostasis, is associated with the naturally evolving diabetic phenotype in this model and is consistent with the mitochondrial impairment observed in chronic metabolic disease. These findings support the utility of the *Myodes rufocanus* model for investigating endogenous metabolic dysregulation and mitochondrial alterations relevant to type 2 diabetes, but they do not imply a direct causal role of COX14. Our study identified *Myodes rufocanus* as a natural model for T2DM and highlighted COX14 as a potential putative modulator of mitochondrial activity in β-cells. We found that the reduction in COX14 expression was associated with altered Complex IV assembly, decreased mitochondrial membrane potential (MMP), diminished ATP production, and increased oxidative stress, which may contribute to the activation of the intrinsic apoptotic pathway and impaired glucose-stimulated insulin secretion. Furthermore, oxidative stress appeared to coincide with suppression of the Nrf2/HO-1 antioxidant defense system, aggravating mitochondrial damage and β-cell dysfunction. These findings outline a potential mechanistic sequence linking mitochondrial impairment to β-cell dysfunction, a key feature of diabetes progression. Beyond establishing a novel model system, our study suggests that COX14 may serve as a candidate biomarker and mechanistic indicator for exploring mitochondrial health and β-cell viability in diabetes. Although there is a consistent association between COX14 downregulation and both mitochondrial dysfunction and diminished insulin secretion, the present findings do not establish a direct causal relationship. It is plausible that COX14 represents a downstream consequence of glucotoxicity or oxidative stress, rather than serving as a primary cause of β-cell dysfunction. Consequently, COX14 should be regarded as a potential candidate biomarker and a foundation for hypothesis generation, pending further validation through targeted knockdown and overexpression studies.

We acknowledge that pancreatic tissue is composed of both endocrine and exocrine cells, which may result in varied gene expression within β cells. To address this, we validated COX14 specifically in the MIN6 β-cell line to ensure the relevance of our findings to β-cells. In the interpretation of transcriptomic data, it is important to consider that RNA sequencing was conducted on whole pancreas tissue. Variations in gene expression may be influenced by alterations in the proportions of endocrine and exocrine cells or by immune cell infiltration, rather than representing intrinsic transcriptional changes within β-cells. The presence of immune-related genes among the upregulated differentially expressed genes is consistent with inflammatory or immune influences on the diabetic pancreatic environment. Consequently, these transcriptomic findings should be regarded as associative and hypothesis-generating. To elucidate cell-type-specific regulatory mechanisms, future research should employ isolated islets or single-cell transcriptomic methodologies. Future studies using isolated islets or single-cell RNA sequencing could further clarify β-cell-specific expression and the function of COX14. Additionally, functional manipulation of COX14, including overexpression or knockdown experiments, is essential to directly establish its causal role in β-cell mitochondrial function and insulin secretion.

## 4. Materials and Methods

Spontaneous type 2 diabetes in *Myodes rufocanus* was characterized by comprehensive metabolic, physiological, and biochemical analyses. Pancreatic RNA sequencing identified COX14 downregulation as a hub gene potentially associated with β-cell dysfunction, which was validated in vivo and in vitro. In MIN6 β-cells under glucotoxic conditions, COX14 downregulation is linked to impaired mitochondrial function, increased oxidative stress, activation of apoptotic pathways, and reduced insulin secretion, highlighting its potential as a biomarker of β-cell mitochondrial health in spontaneous type 2 diabetes.

### 4.1. Reagents and Chemicals

In the current study, a comprehensive assortment of materials and chemicals was used in the experiments. The ACCU-CHEK Guide glucometer (model 929; Mannheim, Germany) and its corresponding test strips were obtained from a local supplier. Guangzhou Ruixin Biotechnology Co., Ltd. (Guangzhou, China) supplied the ELISA kits. A substantial portion of the chemicals and reagents, including primers, D-glucose, DMEM, FBS, penicillin-streptomycin, HEPES, and L-glutamine, was supplied by ServiceBio Technology Co., Ltd., Wuhan, China. The Total RNA Isolation Kit, FastPure Complex Tissue/Cell (RC-113), the HiScript III cDNA synthesis kit (R-323), and SYBR qPCR Universal Master Mix (Q711) for qRT-PCR were acquired from Nanjing Vazyme Biotech Co., Ltd., Nanjing, China. Antibodies specific to COX14 (Youpin, YP-mAb-09782 (Rabbit)), beta-actin (Service bio, GB15003 (Rabbit)), Nrf2 (Service bio, GB113808 (Rabbit)), and HO-1 (Service bio, GB115713 (Rabbit)) were purchased from Henan Zhisheng Youpin Biotechnology Co., Ltd., Zhengzhou, China and ServiceBio Technology Co., Ltd., Wuhan, China. The additional reagents employed in this research were of analytical quality and sourced from authorized suppliers.

### 4.2. Animals, Breeding and Early Diagnosis of Spontaneous Diabetes

The *Myodes rufocanus* voles used in this study were obtained from the Laboratory Animal Care Center (SPF Unit) of Dalian Medical University. A total of 237 *Myodes rufocanus* voles aged 10–30 weeks were screened for spontaneous diabetes using an ACCU-CHEK glucometer (Model 929; Mannheim, Germany). Voles with blood glucose levels > 11.1 mmol/L were classified as diabetic. Five pairs with the best diabetic phenotype were monitored for two weeks and underwent oral glucose tolerance tests (OGTTs) and insulin tolerance tests (ITTs) to assess glucose homeostasis and insulin sensitivity [96]. To stabilize the diabetic phenotype, brother–sister mating was performed from the F1 to F5 generations. By the F6 generation, male voles consistently exhibited a stable diabetic phenotype. Eighteen-week-old F6 males were subsequently selected for experimental studies, providing a reliable spontaneous model of type 2 diabetes. Age-matched control voles were derived from the same breeding colony, screened using identical criteria, and confirmed to be non-diabetic. The study included a total of 12 voles, with six assigned to the diabetes mellitus group and six to the control group (*n* = 6 per group).

Animals were maintained under standardized husbandry conditions appropriate for vole species. Briefly, all animals were housed under specific pathogen-free conditions (22 ± 2 °C, 47 ± 2% humidity, and 12 h light/dark cycles) with unrestricted access to standard chow (LabDiet 5001) and water. Body weight, blood glucose, and serum lipid profiles, including triglycerides, total cholesterol, LDL-C, HDL-C, and free fatty acids, were monitored over a one-month period. Glucose tolerance and insulin resistance were evaluated using OGTTs and the homeostatic model assessment of insulin resistance (HOMA-IR) index, respectively. No animals were selected based on disease severity. Investigators were blinded to group allocation during phenotypic assessments, biochemical analyses, and downstream molecular experiments. No animals or samples were excluded after group assignment. At the end of the study, voles were anesthetized with isoflurane (3–5% for induction; 1–3% for maintenance) and euthanized. Blood samples were collected through enucleation and cardiac puncture. Pancreatic and other tissue samples were immediately excised, frozen in liquid nitrogen, and stored at −80 °C. Serum was separated by centrifugation at 3500 rpm for 15 min. All experimental procedures complied with the Guide for the Care and Use of Laboratory Animals and were approved by the Institutional Animal Care and Use Committee of Dalian Medical University (Approval No. AEE20046).

### 4.3. Diagnosis of Spontaneous Diabetes in Voles

#### 4.3.1. Assessment of Body Weight and Nutritional Intake

After a seven-day acclimation phase, voles from each experimental group were systematically observed over a four-week period. Body weight was measured every three days with a digital scale, while food and water consumption was alternately recorded every third day for a duration of one month.

#### 4.3.2. Blood Glucose Monitoring

Random blood glucose levels were examined on a weekly basis for four weeks at 08:00 AM in the morning. Blood samples were collected through a tail nick procedure and subsequently analyzed using an ACCU-CHEK Guide glucometer (model 929; Mannheim, Germany). Voles with glucose concentrations exceeding 7 mmol/L were identified as susceptible to diabetes, whereas those with levels exceeding 11.1 mmol/L were categorized as diabetic. Fasting blood glucose was measured following an 8 h fast to evaluate metabolic status in accordance with established protocols [97]. Animals having fasting glucose levels >11.1 mmol/L were identified as diabetic. These cutoff-based criteria were applied consistently across all analyses.

#### 4.3.3. Oral Glucose Tolerance Test (OGTT) and Insulin Tolerance Test (ITT)

After a 12 h fast overnight, voles were subjected to an OGTT. At 08:00, baseline blood glucose levels were recorded; subsequently, glucose was administered orally at a dosage of 2 g/kg body weight through gavage. Blood samples were collected from tail nicks at 0, 30, 60, and 120 min following glucose administration to evaluate glucose concentrations. Voles with blood glucose levels > 11.1 mmol/L were identified as diabetic.

For the ITT, voles underwent a 6 h fast before the initial glucose measurement. At 08:00, insulin was injected intraperitoneally at a dosage of 4 U/kg, and the levels of blood glucose were systematically evaluated at 0, 15, 30, 60, and 120 min. Voles with glucose levels > 11.1 mmol/L were considered insulin-resistant.

Insulin resistance was further assessed using the Homeostasis Model Assessment of Insulin Resistance (HOMA-IR) and was calculated using the following equation: HOMA-IR = FBG (mmol/L) × FINS (μU/mL)/22.5. FBG stands for fasting blood glucose and FINS for fasting insulin levels, respectively [98,99].

#### 4.3.4. Insulin, Leptin and Free Fatty Acid (FFA) ELISA

Serum insulin, leptin, and FFA levels were assessed using ELISAs. Blood samples from each experimental group were procured and spun at 3500 rpm at 4 °C for 5 min to obtain the serum. The RUIXIN Mouse Insulin ELISA Kit was used for insulin measurement. The FineTest Mouse Leptin ELISA Kit (EM0129) was used for leptin, and the FineTest Mouse FFA ELISA Kit was used for FFAs. Briefly, samples and standards were introduced into 96-well plates pre-coated with antibodies and incubated to promote antigen–antibody binding. After washing to remove any unbound material, antibodies were added, followed by incubation. Further washing was performed before applying the chromogenic substrate, and the enzymatic reaction was stopped using a stop solution. Absorbance was recorded at the specified spectral region for each kit, and the concentrations were determined using standard curves. Each sample was evaluated twice to guarantee precision.

#### 4.3.5. Lipid Profiling

To examine the metabolic alterations associated with diabetes, serum lipid profiles were thoroughly analyzed, focusing on total cholesterol (TC), triglyceride (TG), low-density lipoprotein cholesterol (LDL-C), and high-density lipoprotein cholesterol (HDL-C). Blood samples were obtained from all experimental groups, left to clot at ambient temperature, and subsequently centrifuged to obtain serum. Lipid concentrations were quantified using commercial assay kits (detailed given in the Chemicals and Reagents Section, in strict accordance with the manufacturer’s guidelines. Briefly, serum samples and standards were combined with enzyme-specific reagents to initiate reactions, producing colorimetric products that were proportional to the lipid content. Following the completion of the reactions, absorbance measurements were taken at 450 nm using a microplate reader, and the lipid concentrations were subsequently determined from standard curves. Each sample was examined twice to confirm precision and reliability.

### 4.4. RNA Sequencing

Pancreatic tissue samples were promptly frozen in liquid nitrogen and stored at −80 °C to maintain their integrity. For RNA sequencing, three samples from each group were selected. Total RNA was extracted from these samples to construct cDNA libraries, which were subsequently sequenced using the Illumina NovaSeq X Plus platform, achieving an average depth of approximately 22 million paired-end reads per sample. The raw sequence reads underwent preprocessing to enhance the accuracy of subsequent analyses, addressing issues such as poor read quality, adapter contamination, and residual primers. FastQC was employed to evaluate the raw reads, examining aspects such as base-specific sequence quality, GC content, N content, sequence length distribution, and duplication levels [100]. Fastp was then utilized to filter and clean the reads prior to alignment, with parameters set for processing speed (−w), input reads (−i), and output location (−o) [101]. Transcript abundance was quantified using RSEM, and raw read count matrices were generated for differential expression analysis. The raw counts were normalized using the median-of-ratios method in DESeq2 to account for variations in library size and sequencing depth. DESeq2 was employed to identify differentially expressed genes between the control and diabetic groups, with significance defined as an absolute log_2_ fold change ≥ 1 and an adjusted *p*-value ≤ 0.05 following Benjamini–Hochberg false discovery rate correction. FPKM values were calculated and utilized solely for expression visualization, correlation analysis, and clustering. All transcriptomic data generated in this study were submitted to the NCBI Bio Project database under accession number PRJNA1236505 and SRP Accession Number SRP570670.

#### 4.4.1. De Novo Transcriptome Assembly

Trinity, a renowned tool for de novo assembly of RNA-seq data, was used to perform de novo transcriptome assembly. Assembly quality was evaluated using Benchmarking Universal Single-Copy Orthologs, which is a widely accepted method for evaluating transcriptome completeness. This analysis involved three datasets: Trinity.fasta, cluster all.fasta, and Unigene. fasta. The Trinity-spliced transcriptome was used as the reference sequence (Ref) to align clean reads from individual samples. Sequences with a comparison quality score below 10 were omitted, and sequences from non-contrast pairs were compared across different genomic regions. The RSEM software (v1.3.1) facilitated the alignment process, utilizing bowtie2 with a mismatch parameter set to 0, whereas all other bowtie2 parameters remained at their default settings. Transcript abundance was estimated using RSEM, and raw read count matrices were used as input for differential expression analysis with DESeq2. FPKM values were calculated and applied exclusively for expression visualization, correlation analysis, and clustering. Unigene annotation was performed using homology-based searches against public databases, with closest matches frequently corresponding to phylogenetically related rodent species. Mitochondrial and OXPHOS-related genes were identified based on conserved orthology and functional annotation.

#### 4.4.2. Differential Gene Correlation, Overlapping and Clustering

The RSEM software, in conjunction with Bowtie2, was used to examine the FPKM distribution patterns across various experimental conditions, ensuring an R2 threshold of 0.8 between biological replicates. Correlation coefficients derived from the FPKM values were used to assess intergroup differences and intragroup consistency. A heatmap was used to depict group differences and replicate similarities, whereas a Venn diagram was used to represent the genes commonly expressed in both the diabetic and control groups. Hierarchical clustering was conducted on the differentially expressed genes from all comparison groups based on their FPKM values. The expression data were subjected to row-wise z-score normalization. Given the substantial number of differentially expressed genes identified, 25,000 genes were selected for representation in a clustering heatmap.

#### 4.4.3. Differential Gene Expression (DGE) Analysis

A comprehensive analysis of DGE was conducted employing DESeq2 between the control and diabetic groups. The investigation identified genes exhibiting significant statistical and biological differences using stringent criteria: an absolute log2 fold change of at least 1 and an adjusted *p*-value not exceeding 0.05. This approach classifies genes that are either upregulated or downregulated. Volcano plots were used to visually depict the most significant genes exhibiting differential expressions, thereby illustrating the expression variations between the groups.

#### 4.4.4. Enrichment Analysis of Gene Functions and Pathways

Differentially expressed genes were analyzed for functional significance using Gene Ontology (GO) and Kyoto Encyclopedia of Genes and Genomes (KEGG) databases through the clusterProfiler package in R. GO analysis categorized enriched terms into the biological process (BP), cellular component (CC), and molecular function (MF) groups. KEGG pathway enrichment analysis was performed to identify the key signaling and metabolic pathways associated with the identified genes. These analyses provided insights into the main biological processes and molecular mechanisms affected by changes in gene expression.

#### 4.4.5. Analysis of Protein Interaction Networks and Hub Gene Screening

Protein interaction network analysis was conducted using STRING, an extensive database that integrates PPI data from various repositories, scholarly articles, and empirical findings. To identify novel mitochondrial candidates, the focus was not placed on the upregulated hub genes, many of which are acknowledged as significant inflammatory regulators in diabetes. Consequently, the downregulated hub genes identified are considered pathway-specific candidates rather than representing a comprehensive network hierarchy. Genes exhibiting downregulation, characterized by a log2 fold change below −1 and an adjusted *p*-value of 0.05 or lower, were inputted into the STRING platform, with all other configurations set to their default values. The resulting PPI network was visualized and analyzed using Cytoscape (https://cytoscape.org/ accessed on 10 April 2025), a prominent tool for investigating biological interactions. We used the CytoHubba plugin in Cytoscape to identify key nodes within these networks. This plugin employs a degree algorithm to evaluate node connectivity within the PPI network of downregulated genes, facilitating the identification of the top 20 hub genes. The significance of each protein in the network was determined by examining its interactions with other proteins, which led to the selection of the 20 most significantly downregulated hub genes. Among these, COX14 emerged as a novel OXPHOS-related candidate owing to its potential role in mitochondrial dysfunction and β-cell impairment in diabetes.

### 4.5. In Vivo Validation in Pancreatic Tissue

To verify the RNA sequencing results, the expression of selected hub genes was examined in pancreatic tissues collected from diabetic and control voles. Total RNA was extracted using an RNA isolation kit (Vazyme RC-113). Complementary DNA (cDNA) was synthesized using HiScript III (Vazyme, R-323). Quantitative real-time PCR was performed using SYBR qPCR Master Mix (Vazyme, Q711) to measure the expression levels of COX14, NRF2, and HO-1, with β-actin as the internal control. The primers were obtained from ServiceBio (Wuhan, China) and are listed in Table 2. Each reaction was performed in triplicate, and relative gene expression was determined using the StepOne Real-Time PCR System software (v2.3).

Western blotting was used to assess the levels of COX14, NRF2, and HO-1 proteins using specific primary antibodies (β-actin: Service Bio, GB15003 (Rabbit); COX14: Youpin, YP-mAb-09782 (Rabbit); Nrf2: Service Bio, GB113808 (Rabbit); and HO-1: Service Bio, GB115713 (Rabbit)), as detailed in Table S3. Proteins were isolated using the RIPA lysis buffer and subsequently separated on 8–12% gradient gels via SDS-PAGE. After electrophoresis, the separated proteins were transferred onto a PVDF membrane for immunoblotting. To prevent nonspecific binding, the membrane was blocked with 5% skim milk for approximately two hours and rinsed several times with TBST. The samples were then incubated overnight at 4 °C with the primary antibody. Following this, the membrane was washed repeatedly to remove any unbound antibody and then exposed to a suitable secondary antibody for one hour at room temperature. The excess secondary antibody was removed by additional washing. Protein bands were detected using an enhanced chemiluminescence (ECL) reagent, and the signals were recorded using a gel documentation system. Each experiment was performed in triplicates to ensure reproducibility.

Apoptotic signaling was evaluated by determining the BAX/BCL2 ratio at the mRNA level using qRT-PCR. The Caspase-3 Assay Kit (Abcam, ab39383) was employed to assess caspase-3 activity according to the company’s guidelines. Each sample was analyzed independently three times.

### 4.6. MIN6 Cell Culturing and In Vitro Validations of Target Genes

MIN6 mouse insulinoma cells were procured from ServiceBio, Wuhan, China, and cultured in DMEM containing glucose (5 mM), 10% fetal bovine serum (FBS), 1% HEPES, penicillin–streptomycin, and L-glutamine (ServiceBio, Wuhan, China) under standard conditions of 37 °C and 5% CO_2_. Following recovery, the cells were grouped into two experimental categories: the control group was maintained at 5 mM glucose, and the high-glucose group was exposed to 30 mM glucose to simulate diabetic stress over a 48 h period. All in vitro experiments were performed using MIN6 cells within a defined passage range (passages 18–30) that is widely accepted to preserve stable insulin secretion and mitochondrial function [102,103]. Validation in MIN6 cells was performed using RT-qPCR and Western blot techniques to evaluate COX14, NRF2, and HO-1 expression, whereas apoptotic markers were evaluated by analyzing the BAX/BCL2 ratio at the mRNA level and caspase-3 activity under glucotoxic conditions, employing the same kits and antibodies as previously described in the in vivo study. All samples were subjected to triplicate analysis, and the StepOne System software (v2.3) was used to quantify the relative levels of gene expression. Protein bands were detected using an enhanced chemiluminescence (ECL) substrate, and images were acquired using a gel documentation instrument.

### 4.7. Functional Assays in MIN6 Cells

#### 4.7.1. Cell Viability

To determine the optimal glucose concentration and exposure duration for MIN6 cells, the cells were incubated with varying concentrations of glucose (5, 10, 15, 20, 25, and 30 mM) over time intervals ranging from 24 to 72 h. Cell Counting Kit-8 (ServiceBio, Wuhan, China) was used to assess cell viability. A microplate reader (BioTek, Winooski, VT, USA) was used to measure the absorbance at 450 nm in strict accordance with the manufacturer’s guidelines. A marked decline in the viability of cells was noted at 30 mM glucose after 48 h, which was established as the standard condition for future experimental setups. Further validation was conducted using the Calcein-AM/PI Assay Kit (ServiceBio, Wuhan, China). After a 48 h treatment period, viable cells were labeled with Calcein-AM, which fluoresces green, whereas non-viable cells were labeled with propidium iodide (PI), which fluoresces red, and images were captured using a fluorescence microscope. Quantification was performed using the ImageJ software (v1.53t).

#### 4.7.2. Assessment of Mitochondrial Membrane Potential (MMP)

The MMP JC-1 Assay Kit (ServiceBio, Wuhan, China) was used to evaluate MMP while strictly adhering to the manufacturer’s protocol. In brief, after a 48 h treatment period, MIN6 cells were exposed to the JC-1 dye, which preferentially gathers within the mitochondria, contingent upon their membrane potential. Mitochondria with intact membrane potentials form red fluorescent J aggregate, whereas depolarized or compromised mitochondria exhibit green, fluorescent monomers. Fluorescence was captured using a fluorescence microscope, and the ratio of red-to-green fluorescence intensity was computed to quantitatively assess the changes in MMP.

#### 4.7.3. Reactive Oxygen Species (ROS) Production

After a 48 h treatment period, the levels of ROS in MIN6 cells were assessed by employing the DCFH-DA probe (ROS Assay Kit; ServiceBio, Wuhan, China) following the manufacturer’s guidelines. Once inside the cell, DCFH-DA undergoes deacetylation by cellular esterases, resulting in its conversion to DCFH (non-fluorescent). This compound subsequently undergoes oxidation by ROS, resulting in the formation of a fluorescent DCF. Fluorescence intensity, which indicates the concentration of intracellular ROS, was captured using fluorescence microscopy and quantified using the ImageJ software.

#### 4.7.4. ATP Content

ATP concentration was measured using an ATP Content Detection Kit (G4309, ServiceBio, Wuhan, China), which relies on a luciferase-based luminescence reaction, in strict accordance with the manufacturer’s guidelines. Concisely, MIN6 cells were subjected to lysis after a 48 h exposure period to 30 mM glucose, and the ATP in the lysates was reacted with luciferase to emit light proportional to the ATP concentration. Luminescence was recorded in relative light units (RLU) using a microplate reader in luminescence detection mode (BioTek, Winooski, VT, USA), and the ATP concentration in the diabetic cohort was adjusted to match that in the control cohort.

#### 4.7.5. Complex IV Activity

The activity of Complex IV in MIN6 cells was evaluated after a 48 h treatment period using the Abcam Complex IV Rodent Activity Assay Kit (ab109911) in strict accordance with the manufacturer’s guidelines. Briefly, following 48 h of exposure to 30 mM glucose, the cell lysates were directly incubated with reduced cytochrome c. Enzymatic oxidation of cytochrome c was monitored spectrophotometrically at 550 nm, providing a quantitative assessment of Complex IV activity.

#### 4.7.6. β-Cell Secretory Capacity

Insulin secretion was measured using glucose-stimulated insulin secretion (GSIS) after a 48 h treatment period. MIN6 cells (passages 18–30) were sequentially incubated in Krebs–Ringer bicarbonate buffer (G0430, ServiceBio, Wuhan, China), initially with a low glucose concentration of 5 mM, followed by a high glucose concentration of 30 mM for 1 h each. Insulin levels were analyzed in the collected supernatants using an Insulin ELISA Kit (Ruixin Biotech, Guangzhou, China). The insulin secretion index was determined by comparing insulin release at high glucose levels with that at low glucose levels.

### 4.8. Statistical Analysis

Data are presented as the mean ± SEM or the mean ± SD, as specified in figure legends, with biological replicates (n) and technical replicates clearly indicated. For in vivo experiments, n represents individual animals per group; for in vitro experiments, n represents independent experimental replicates. Technical triplicates were performed for biochemical assays (serum analyses, RT-qPCR, Western blots, and enzyme activities) and averaged for analysis. For comparisons between two groups, unpaired two-tailed *t*-tests with Welch’s correction were used, whereas two-way ANOVA with post hoc tests was applied for experiments involving multiple groups or time points. Assumptions of normality and variance homogeneity were confirmed, and adjustments for multiple comparisons were implemented when analyzing related endpoints (ATP, Complex IV, the JC-1 ratio, ROS, and GSIS). Statistical analyses were performed using GraphPad Prism (version 10). A *p*-value of less than 0.05 was considered statistically significant (* *p* < 0.05, ** *p* < 0.01, *** *p* < 0.001, and **** *p* < 0.0001).

## 5. Conclusions

Using RNA sequencing, computational analysis, and functional validation, we identified *Myodes rufocanus* as a natural model for T2DM and found that COX14 represents a hypothesis-generating candidate associated with β-cell mitochondrial integrity, redox homeostasis, and insulin secretion. The downregulation of COX14, as observed in RNA-seq data, correlates with alterations in Complex IV assembly, loss of mitochondrial membrane potential, reduced ATP production, and increased ROS levels. Concurrently, suppression of the Nrf2–HO-1 antioxidant pathway may exacerbate oxidative damage, potentially contributing to β-cell apoptosis and impaired insulin secretion in response to glucose. These findings suggest a mechanistic association in which mitochondrial dysfunction and weakened antioxidant defenses converge to affect β-cell viability, although direct functional manipulation of COX14 is required to establish causality. In addition to introducing a novel spontaneous T2DM model, COX14 emerges as a candidate biomarker worthy of further investigation for its potential role in preserving mitochondrial resilience and β-cell function in diabetes.

## 6. Limitations of the Study

Although *Myodes rufocanus* represents a promising natural model of type 2 diabetes, several limitations should be noted. RNA sequencing was conducted on whole pancreas tissue, which contains multiple cell types; thus, the observed transcriptional changes, including COX14 downregulation, may reflect shifts in cellular composition rather than β-cell-intrinsic regulation. Accordingly, the transcriptomic data should be considered associative. In addition, while COX14 downregulation was validated at the mRNA and protein levels and supported by functional mitochondrial assays, the lack of overexpression studies precludes definitive conclusions regarding its causal role in β-cell mitochondrial dysfunction. Further cell-type-specific and gain-of-function studies are required to clarify the mechanistic role of COX14 in this model.

## Figures and Tables

**Table 1:** The expression level of COX14 in diabetes vs. control voles along with the respective ranks and scores.

Hub Genes	Rank	Score	Expression (logFC)
COX14	5	13	−2.2865

**Table 2:** Primer sequences used for qPCR validation.

Target Gene	Forward Sequence (5′-3′)	Reverse Sequence (5′-3′)
β-ACTIN	ACCCACACTGTGCCCATCTA	ATGTCACGCACGATTTCCCT
COX14	TGGCTACAAGACCTTCTCTGC	CAGCTGCAAATAACGGTAGGC
NRF2	AGCAGGACATGGAGCAAGTT	TTCTTTTTCCAGCGAGGAGA
HO-1	CCCACCAAGTTCAAACAGCTC	AGGAAGGCGGTCTTAGCCTC
BAX	GCAGCGGCAGTGATGGAC	GCAAAGTAGAAGAGGGCAACC
BCL2	ACCCTCCTGATTTTTCCTCCACCTA	AATACATAAGGCAACCACACCATCG

## Data Availability

Data used to support the findings of this study are available from the corresponding author upon request. The RNA-seq data generated in this study have been deposited in BioProject PRJNA1236505 (SRP accession SRP570670). Supplementary File S1 includes the DESeq2 design formula, filtering steps, and the full list of DEGs.

## Supplementary Materials

The following supporting information can be downloaded at: https://www.mdpi.com/article/10.3390/ijms27031539/s1.
