# Supplementary material for: Multi-Omics Analysis of a Spontaneous Type 2 Diabetes Model in Myodes rufocanus and Its Underlying Mechanisms
Source: Int J Mol Sci. 2026 Feb 4;27(3):1539. doi: 10.3390/ijms27031539 (PMC12898206; doi:10.3390/ijms27031539)
Supplement: Supplementary file 1 [file ijms-27-01539-s001.zip › Suplementary Data.pdf]

Top 10 upregulated and Downregulated genes

Table S1: The top 10 upregulated and Downregulated genes of Diabetes vs Control group with their respective logFC and p-values.

Genes	logFC	P-value	Expression
IGH	8.2841	0.034004	Upregulated
IL21R	7.3371	0.014399	Upregulated
ITGA4	7.2494	5.77E-06	Upregulated
TOP2	6.9349	2.17E-06	Upregulated
RSKL	6.8082	3.38E-05	Upregulated
WDFY4	6.1304	6.99E-05	Upregulated
CD4	6.0447	0.046485	Upregulated
BCL11B	5.8572	0.0016944	Upregulated
RUBCNL	5.7944	0.0016843	Upregulated
TRAF1	5.7779	0.049169	Upregulated
REG3	-9.1254	0.0053674	Downregulated
REG1	-7.8394	6.56E-12	Downregulated
CFAP57	-7.657	2.95E-07	Downregulated
CLDN	-6.0576	7.00E-05	Downregulated
CA	-6.0314	2.76E-07	Downregulated
TTLL9	-5.7381	0.014942	Downregulated
KRAB	-5.157	0.0016113	Downregulated
GCGR	-5.1456	0.036725	Downregulated
TEX15	-4.923	0.026126	Downregulated
H2B	-4.7829	3.62E-06	Downregulated

Top 20 Downregulated Hub Genes

Table S2: The top 20 Downregulated Hub Genes with their respective ranks and scores.

Rank	Name	Score
1	Ndufa2	20
2	Ndufa6	19
3	Ndufa11	15
4	Coa6	14
5	Cox14	13
5	Ndufa1	13
7	Ndufb4	12
8	Atp5md	11
8	Cox7c	11
10	Timm10	10
10	Cox17	10
12	Ndufb2	9
13	Mrpl55	8
13	Polr2l	8
13	Slirp	8
16	Atox1	7
16	Polr2k	7
18	Cox19	6
18	Timm17b	6
20	Mrps36	5

List of Antibodies

Table S3: List of Antibodies used in western blot to check gene expression at Protein level

Antibody	Manufacturer	Catalog No.	Dilution	Secondary Antibody	Secondary Catalog No.	Secondary Dilution
β-Actin	Servicebio	GB15003	1:3000	HRP-conjugated Goat anti-Rabbit	GB23303	1:3000
COX14	YopeBio	YP-mAb-09782	1:1000	HRP-conjugated Goat anti-Rabbit	GB23303	1:3000
NRF2	Servicebio	GB113808	1:1000	HRP-conjugated Goat anti-Rabbit	GB23303	1:3000
HO-1	Servicebio	GB115713	1:1000	HRP-conjugated Goat anti-Rabbit	GB23303	1:3000

Table S4. Comparison of commonly used rodent models of type 2 diabetes and the spontaneous Myodes rufocanus model.

Feature	Chemically Induced (STZ)	Diet-Induced (HFD)	Transgenic / Monogenic	Spontaneous Myodes rufocanus
Disease induction	Chemical β-cell toxicity	High-fat feeding	Genetic manipulation	Natural, spontaneous onset
Genetic manipulation	No	No	Yes (single or multiple genes)	No
Disease onset	Rapid, artificial	Gradual, diet-dependent	Predetermined by genotype	Progressive, age-dependent
Hyperglycemia	Severe, abrupt	Moderate to severe	Variable	Stable, chronic
Insulin resistance	Limited	Prominent	Variable	Prominent and progressive
β-cell dysfunction	Acute loss	Functional decline	Gene-specific	Progressive dysfunction
Mitochondrial dysfunction	Secondary	Diet-associated	Gene-dependent	Intrinsic and progressive
Oxidative stress	Acute	Chronic	Variable	Chronic, endogenous
Model relevance to human T2DM	Limited	Moderate	Mechanistic	High (polygenic, non-induced)
