# Supplementary material for: Multi-Omics Analysis of a Spontaneous Type 2 Diabetes Model in Myodes rufocanus and Its Underlying Mechanisms
Source: Int J Mol Sci. 2026 Feb 4;27(3):1539. doi: 10.3390/ijms27031539 (PMC12898206; doi:10.3390/ijms27031539)
Supplement: Supplementary file 1 [file MIN6 Cells Quality Report.pdf › Supplementary Review File S1/MIN6 Cells Quality Report.pdf]

细胞质检报告

Product Test Report（Cell）

细胞名称（英文/中文）<br>Product name	MIN6<br>小鼠胰岛β细胞	细胞货号<br>Cat number	STCC20029
生长特性<br>Growth characteristics	贴壁	细胞形态<br>Cell morphology	上皮细胞样
细胞总量<br>Total cell	>1×10^6cells/T25 培养瓶（冻存管）	出库日期<br>The delivery dat	2025.04.08

检验依据：无菌：2020 药典四部《1101 无菌检查法》薄膜过滤法；细菌内毒素：2020 药典四部《1143 细菌内毒素检查法》凝胶法。

Test basis:Sterility: 2020 Pharmacopoeia four "1101 Sterility test Method" membrane filtration method; Bacterial endotoxin: 2020 Pharmacopoeia IV Section 1143 Bacterial endotoxin Test, gel method.

检验项目 Test items

检验指标<br>Test index		检验标准<br>Test standard	检验结果<br>Test result	判定<br>Decision
支原体<br>Mycoplasma		阴性<br>Negative	阴性<br>Negative	合格<br>Qualified
无菌检测<br>Sterility testing		阴性<br>Negative	阴性<br>Negative	合格<br>Qualified
内毒素<br>Endotoxin		≤ 1EU/mL	< 1EU/mL	合格<br>Qualified
细胞活力<br>The cell vitality		> 90 %	> 90 %	合格<br>Qualified
细胞生长实验<br>Cell growth experiment		细胞形态<br>Cell morphology	正常<br>Normal	合格<br>Qualified
		细胞增殖<br>Cell proliferation	正常<br>Normal	合格<br>Qualified

检验结果：

根据《药典》中检验项目进行检验，所有检验项目均符合要求，因此此批产品检验合格，可入库发货。

Test result:

According to the inspection items in the "pharmacopoeia", all inspection items meet the requirements, so this batch of products has passed the inspection and can be put into storage for delivery.

质检专用章 Special stamp for qualify inspection

签发日期 Issuing date: 2025.04.08

检验：马永欣 审核：王迪 批准：贡笑笑 表单编号：SWE-QA-SMP-006-03

Inspected by: Ma Yongxin Checked by: Wang Di Approved by: Gong Xiaoxiao No: SWE-QA-SMP-006-03

CamScanner
