# Supplementary material for: Multi-Omics Analysis of a Spontaneous Type 2 Diabetes Model in Myodes rufocanus and Its Underlying Mechanisms
Source: Int J Mol Sci. 2026 Feb 4;27(3):1539. doi: 10.3390/ijms27031539 (PMC12898206; doi:10.3390/ijms27031539)
Supplement: Supplementary file 1 [file Report_X101SC24097333-Z01-F001-B1-16.pdf › Supplementary Review File S1/Report_X101SC24097333-Z01-F001-B1-16.pdf]

1 项目简介
2 建库测序流程
3 信息分析流程
4 分析结果
5 Appendix
6 参考文献

无参转录组分析报告

合同编号	H101SC24097333
合同名称	大连医科大学6个无参转录组分析技术服务（委托）合同
分期编号	X101SC24097333-Z01-F001-B1-16
参考基因组及版本	Myodes rufocanus
报告时间	2024-10-01
售后服务电话	400-658-1585
售后服务邮箱	service-rna@novogene.com
售后服务平台	https://magic.novogene.com/<br>说明：收到数据释放邮件的下一个工作日，您将收到售后工具平台权限开通的邮件，<br>届时您可以根据邮件提示登录云平台并进行数据分析；云平台操作说明及常见问题请点击magic
温馨提示	Report仅供展示部分结果，全部分析内容详见Result文件夹；<br>在您拿到Result前Report中的Result超链接无效，确认结算后，释放结果文件Result中Report的结果

1 项目简介

转录组是指特定组织或细胞在某个时间或某个状态下转录出来的所有RNA的总和，主要包括mRNA和非编码RNA。由于自然界物种繁多且一些生物的基因组组装困难，目前我们无法获取所有生物的完整基因组。使用有参转录组的分析策略来分析参考基因组组装较差的物种，会导致序列比对率低，许多基因无法被检测到，从而浪费大量的数据。真核无参转录组测序，就是针对无参考基因组或参考基因组组装较差物种的一种较好的分析策略。

真核无参转录组测序采用Illumina测序平台，对真核生物的所有RNA进行测序，根据高质量数据进行拼接，将拼接得到的最长转录本unigene作为参考序列，进一步进行表达定量、功能注释、SNP及SSR等分析。转录组研究是基因功能及结构研究的基础和出发点，真核无参转录组测序为无参考基因组物种的转录水平变化、分子机制及调控网络研究提供了有力的技术手段，目前已广泛应用于基础研究、临床诊断、药物研发和分子育种等领域。

项目整体流程图如下所示：

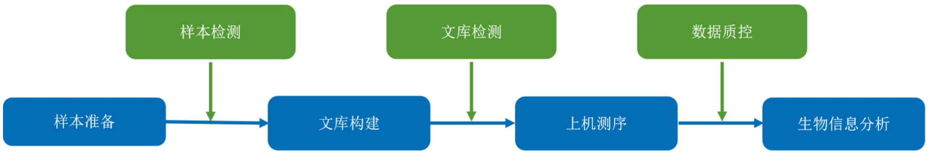

2 建库测序流程

2.1 样品检测

我司采用标准提取方法从组织或细胞中提取RNA，随后对RNA样品进行严格质控，质控方式主要是通过Agilent 2100 bioanalyzer：精确检测RNA完整性。

注：以RNA完整性和总量为主要参考指标，详见样本检测报告

2.2 文库构建以及上机测序

mRNA 的获取主要有两种方式：一是利用真核生物大部分 mRNA 都带有 polyA 尾的结构特征，通过 Oligo(dT) 磁珠富集带有 polyA 尾的 mRNA。二是从 Total RNA 中去除核糖体 RNA，从而得到 mRNA。随后在 Fragmentation Buffer 中用二价阳离子将 mRNA 随机打断，按照普通建库方式或链特异性建库方式进行建库(Parkhomchuk et al., 2009)。

普通建库
使用 Oligo dT 磁珠从总 RNA 中富集 mRNA。在片段化之后，使用随机六聚体引物合成第一链 cDNA，随后进行第二链 cDNA 的合成。经过末端修复、加A尾、接头连接、片段选择、扩增和纯化后，文库准备就绪。

链特异性建库
使用 Oligo dT 磁珠从总 RNA 中富集 mRNA。在片段化之后，使用随机六聚体引物合成第一链 cDNA。然后，使用 dUTP 而不是 dTTP 合成第二链 cDNA。经过末端修复、加 A 尾、接头连接、片段选择、扩增和纯化后，链特异性文库准备就绪。

1 项目简介
2 建库测序流程
3 信息分析流程
4 分析结果
5 Appendix
6 参考文献

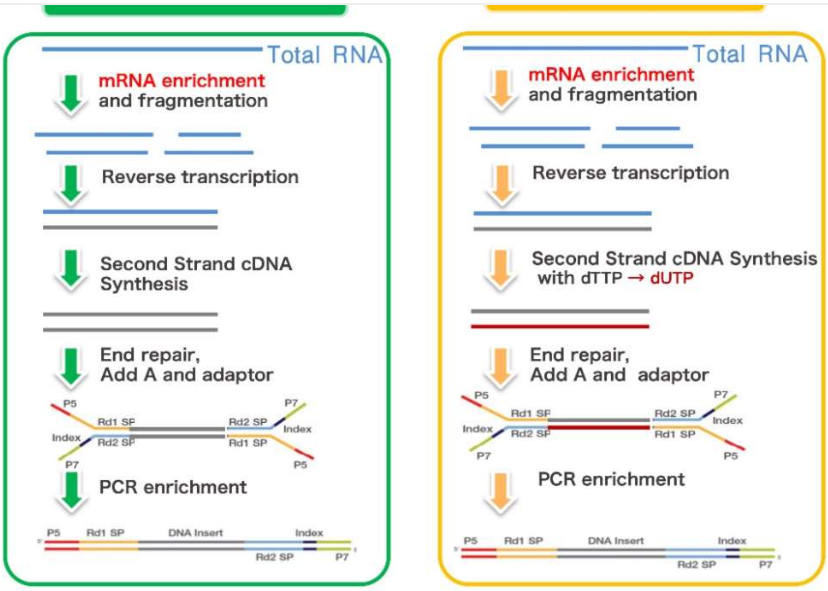

图2.1 文库构建原理示意图

注：测序接头：包括P5/P7，index和Rd1/Rd2 SP三个部分（如上图所示）。其中P5/P7是PCR扩增引物及flow cell上引物结合的部分，index提供区分不同文库的信息，Rd1/Rd2 SP即read1/read2 sequence primer，是测序引物结合区域，测序过程理论上由Rd1/Rd2 SP向后开始进行。

文库构建完成后，先使用Qubit2.0 Fluorometer进行初步定量，稀释文库至1.5ng/ul，随后使用Agilent 2100 bioanalyzer对文库的insert size进行检测，insert size符合预期后，qRT-PCR对文库有效浓度进行准确定量（文库有效浓度高于1.5nM），以保证文库质量。文库构建流程图如下：

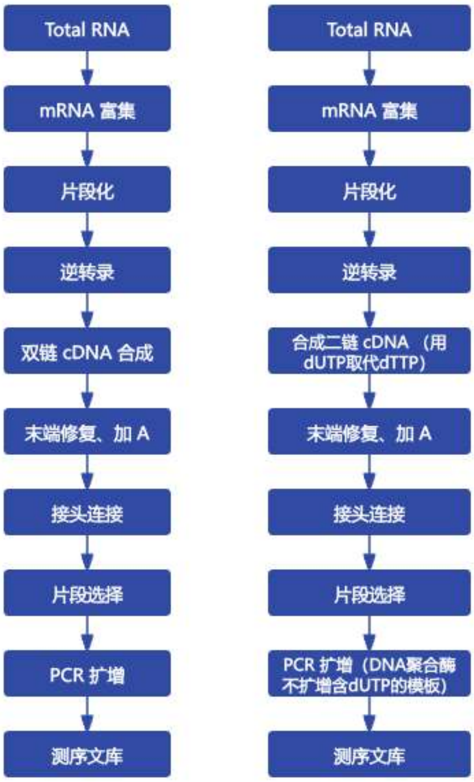

图2.2 普通建库流程图（左）和链特异性建库流程图（右）

库检合格后，把不同文库按照有效浓度及目标下机数据量的需求pooling后进行Illumina测序。测序的基本原理是边合成边测序（Sequencing by Synthesis）。在测序的flow cell中加入四种荧光标记的dNTP、DNA聚合酶以及接头引物进行扩增，在每一个测序簇延伸互补链时，每加入一个被荧光标记的dNTP就能释放出相对应的荧光，测序仪通过捕获荧光信号，并通过计算机软件将光信号转化为测序峰，从而获得待测片段的序列信息。测序过程如下图所示:

1 项目简介
2 建库测序流程
3 信息分析流程
4 分析结果
5 Appendix
6 参考文献

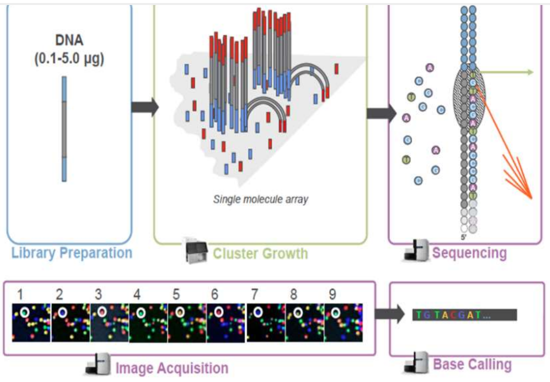

图2.3 Illumina测序原理示意图

显示QA

3 信息分析流程

对于无参考基因组的转录组分析，可先将测序所得的序列拼接成转录本，用Corset程序转录本进行层次聚类，以聚类后序列为参考，进行后续的分析，过程包括质控、七大数据库注释（NR、NT、KO、SwissProt、PFAM、GO及KOG）、定量、差异显著性分析、功能富集等环节，另外变异位点，SSR也是无参转录组的分析内容。同时，我们根据不同的研究需求，推出转录组个性化分析内容，如基因共表达网络构建（WGCNA）、基因表达趋势分析（STEM）、ipath代谢途径可视化分析等。信息分析流程图如下：

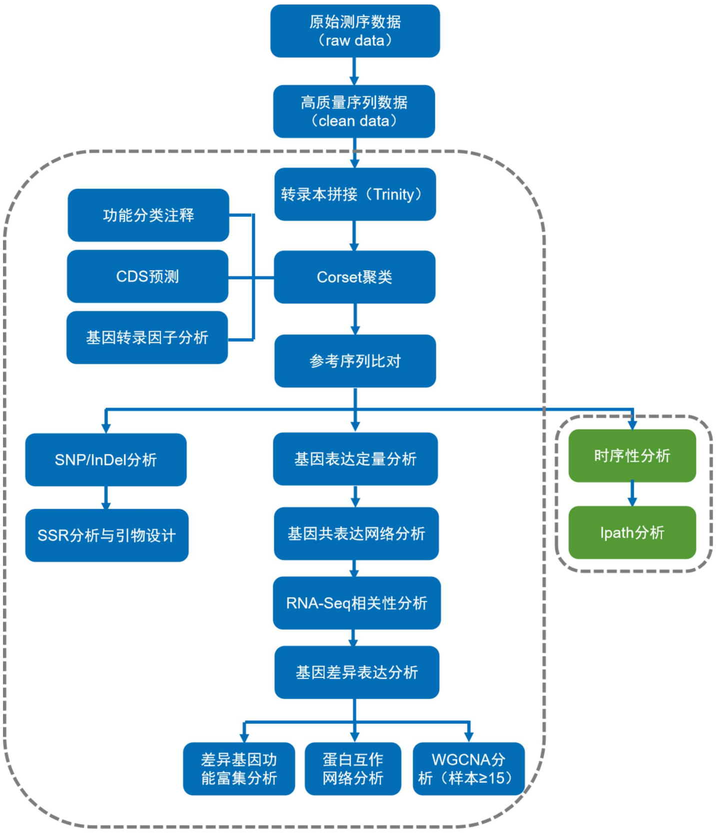

图3 RNA-seq无参信息分析流程图

研究中涉及的方法见中文版methods和英文版methods

4 分析结果

4.1 测序数据质控

4.1.1 原始序列数据

测序片段被高通量测序仪测得的图像数据经CASAVA碱基识别转化为序列数据（reads），文件为fastq(Cock PJ et al., 2010)格式，其中主要包含测序片段的序列信息以及其对应的测序质量信息。

1 项目简介
2 建库测序流程
3 信息分析流程
4 分析结果
5 Appendix
6 参考文献

```
@A00564:177:HTF77DSXX:2:1101:1416:1031 1:N:0:CCTAATCC+GTACGCAA
TNTAGATCTTAGGCGGTTGGTGTAGGTATCGACACAGGTCTTCATTTTGGCTAATAATGTACCAACGGAAGTCTGACATTCTGACTGTTCTTCTTCCTC
+
F#FFFFFFFFFFFFFFFFFFFFFFFFFFFFFFFFFFFFFF:FFFFF:FFFFFFFFFFFFFFFFFFFFFFFFFFFFFFFFFF:FFFFFFFFFFFFFFFFF
```

上述文件中第一行以“@”开头，随后为Illumina测序标识符(Squence Identifiers)和描述文字；第二行是测序片段的碱基序列；第三行以“+”开头，随后为Illumina测序标识符(也可为空)；第四行是测序片段每个碱基相对应的测序质量值，该行每个字符对应的ASCII值减去33，即为该碱基的测序质量值。

4.1.2 测序数据过滤

测序获得的原始数据中包含少量带有测序接头或测序质量较低的reads，如下图所示。为了保证数据分析的质量及可靠性，需要对原始数据进行过滤，过滤内容如下：

（1）去除带接头(adapter)的reads；

（2）去除含N(N表示无法确定碱基信息)的reads；

（3）去除低质量reads(Qphred <= 20的碱基数占整个 read 长度的 50%以上的 reads)。

每个样本的测序数据过滤情况如下：

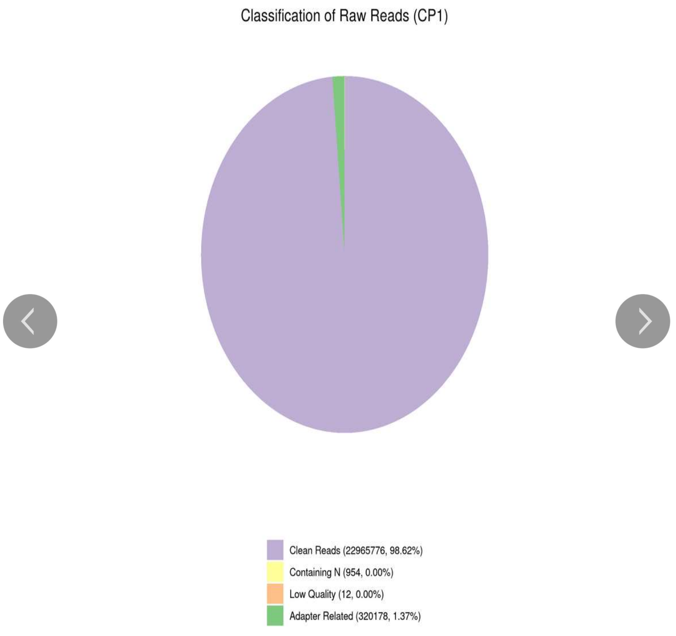

Search..
CP1
CP2
CP3
DP1
DP3
DP2

图4.1 样本测序数据过滤情况

注：图中不同颜色比例分别代表不同组分的比例

（1）**Adapter related**：带接头的reads所占比例。

（2）**Containing N**：带N碱基的reads所占比例。

（3）**Low quality**：测序质量低的reads所占比例。

（4）**Clean reads**：clean reads所占比例。

结果目录：Result/1.QC/3.Filter

4.1.3 测序错误率分布

测序过程本身存在机器错误的可能性，测序错误率分布检查可以反映测序数据的质量，序列信息中每个碱基的测序质量值保存在fastq文件中。如果测序错误率用e表示，Illumina的碱基质量值用Qphred表示，则有：Qphred = $-10\log_{10}(e)$。

1 项目简介
2 建库测序流程
3 信息分析流程
4 分析结果
5 Appendix
6 参考文献

表4.1 Illumina Casava 1.8版本碱基识别与Phred分值之间的简明对应关系

Phred分值	不正确的碱基识别	碱基正确识别率	Q-sorce
10	1/10	90%	Q10
20	1/100	99%	Q20
30	1/1000	99.9%	Q30
40	1/10000	99.99%	Q40

当前RNA-seq测序技术，测序错误率分布存在以下两个特征。

（1）测序错误率随着测序序列(Sequenced Reads)长度的增加而升高。这是由测序过程中化学试剂的消耗导致的，为Illumina高通量测序平台所具有的特征。

（2）前6个碱基具有较高的测序错误率，此长度恰好为RNA-seq建库过程中反转录所需的随机引物长度。前6个碱基测序错误率较高是因为随机引物和RNA模版的不完全结合(Jiang L et al., 2011)。

此特征为illumina高通量测序平台的共有特征。本项目测序数据的错误率分布图如下：

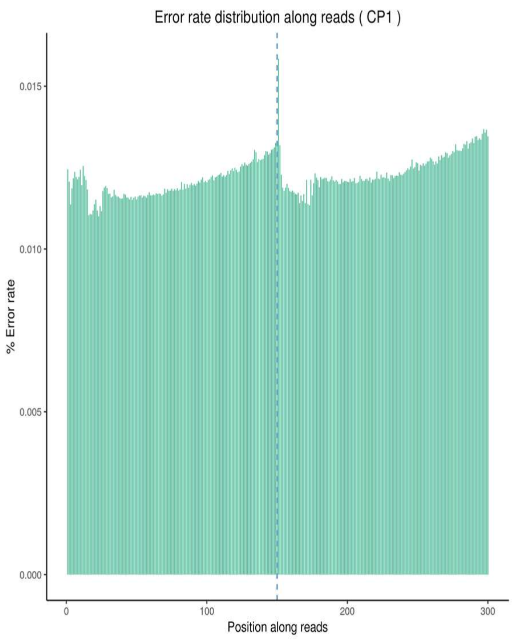

Search..
CP1
CP2
CP3
DP1
DP3
DP2

图4.2 测序数据错误率分布

注：图中横坐标为reads的碱基位置，纵坐标为单碱基错误率

4.1.4 A/T/G/C含量分布检查

核苷酸序列中鸟嘌呤(G)和胞嘧啶(C)所占的比例称为GC含量。GC含量在物种间存在一定特异性，但由于反转录过程中所使用的6bp随机引物，会引起前几位碱基在核苷酸组成上有一定偏好性，产生正常波动，随后则趋于稳定。对于NEB普通建库方法，由于序列的随机性打断和双链互补等原则，理论上测序读段在每个位置的GC及AT含量应分别相等，且在整个测序过程基本稳定不变，呈水平线。而对于链特异性建库而言，由于只保留了单链信息，可能会出现AT分离或GC分离现象。本项目各个样本的GC含量分布如下图所示。

1 项目简介
2 建库测序流程
3 信息分析流程
4 分析结果
5 Appendix
6 参考文献

CP1
CP2
CP3
DP1
DP3
DP2

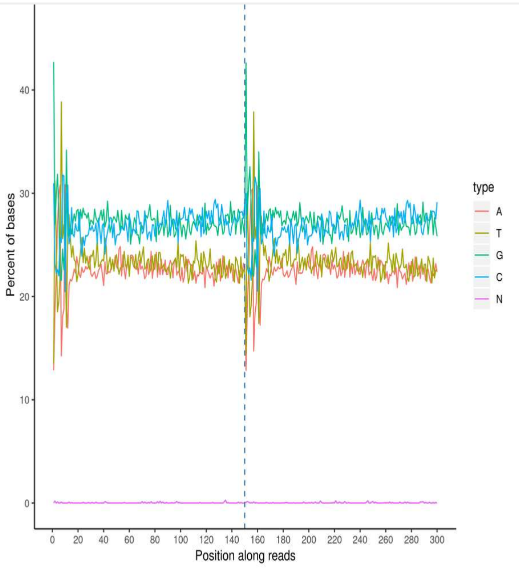

图4.3 样本的GC含量分布

注：图中横坐标为reads的碱基位置，纵坐标为ATGCN五种碱基类型的百分比，不同颜色代表不同的碱基类型

结果目录：Result/1.QC/2.GC

4.1.5 数据质量汇总

经过原始数据过滤、测序错误率检查、GC含量分布检查，获得后续分析使用的clean reads，数据汇总表如下所示。

表4.2 样本测序数据质量汇总

sample	library	raw_reads	raw_bases	clean_reads	clean_bases	error_rate	Q20
CP1	FRAS240248624-1r	23286920	6.99	22965776	6.89	0.01	98.56
CP2	FRAS240248625-1r	20745025	6.22	20187538	6.06	0.01	98.46
CP3	FRAS240248626-1r	19650982	5.90	19052836	5.72	0.01	98.57
DP1	FRAS240248627-1r	22464897	6.74	21822533	6.55	0.01	98.58
DP3	FRAS240248629-1r	23856677	7.16	23128172	6.94	0.01	98.49
DP2	FRAS240259915-1r	26757682	8.03	26111713	7.83	0.01	98.53

注：此表仅对样本测序数据质控情况进行了部分展示，在网页版结题报告中，我们对该表格设置了Copy/Print/Download以及Search功能，点击Copy可以复制表格中数据，用户可以任意新建excel或文本文档，可以将数据粘贴其中，并保留原有的行和列的格式；点击Print则可以打印表格中数据（链接打印机的情况下）；点击Download可以以CSV、Excel、PDF的三种格式下载表格中数据。在Search方框中输入指定信息，则会在下方表格中显示包含该信息的所有内容。以下表格中涉及的Copy/Print/Search功能与此处相同。

显示注释

结果目录：Result/1.QC/4.Stat/data_table.xls

数据产出及质控讲解视频：链接: https://pan.baidu.com/s/1bbouicRwQ10ua95VpqCdkw 提取码: fhsx

显示QA

4.2 转录本拼接

对于无参考基因组的项目，获得clean reads后，需要对clean reads进行拼接以获取后续分析的参考序列。我们采用Trinity (Grabherr MG et al., 2011)对clean reads进行拼接。为了识别个别样品中低表达或者只测到部分片段的基因，对于同物种的所有样品进行合并组装，可以使组装结果更全面，并有利于差异分析等后续分析；而对于不同物种的样品，由于基因组间差异较大，适合分别组装。

4.2.1 拼接过程

1 项目简介
2 建库测序流程
3 信息分析流程
4 分析结果
5 Appendix
6 参考文献

和Butterfly。主要过程如下：

(1) Inchworm：首先，读入所有的 reads 的 fq 文件，然后将 fq 的 reads 文件转换成 fa 格式的 reads 文件，并将 3 端的 reads 和 5 端的 reads 进行合并得到 both.fa。然后，将 reads 按照 k-mers (k bp 长度的短片段)进行分解，统计 kmer 的种类和每一类 kmer 的数目，根据 kmer 的频数按照从高到底进行排序。选取频数最高的 kmer 作为 seed 开始向 3 端延伸，每次延伸一个碱基，统计延伸之后每种 kmer 出现的次数，选择 kmer 频数最高的那一条 kmer 作为延伸路径，最后根据贪婪算法，利用 Overlap 的关系对 k-mers 进行延伸，形成 contig 序列。

(2) Chrysalis：聚类所有相似区域大于k-1-mers 的 contigs，构成 components，依据不同的 components 构建 de Bruijn graph ，并将 reads 与 components 进行比对，进行验证。

(3) Butterfly：列举转录本，拆分 graph 为线性序列，使用 reads 以及 pairs 关系消除错误序列，化简每个 component 的 de Bruijn graph，输出可变剪切亚型的全长转录本，并梳理对应于旁系同源基因的转录本，最终得到拼接结果文件：TRINITY.fasta。

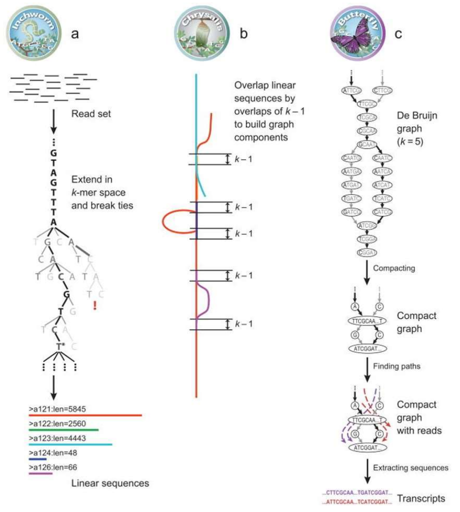

Figure 1 Overview of Trinity. (**a**) Inchworm assembles the read data set (short black line, top) by greedily searching for paths in a k-mer graph (middle), resulting in a collection of linear contigs (color lines, bottom), with each k-mer present only once in the contigs. (**b**) Chrysalis pools contigs if they share at least one k-1-mer and reads span the join, and builds individual de Bruijn graphs from each pool (colored lines). (**c**) Butterfly takes each de Bruijn graph from Chrysalis (top), and trims spurious edges and compacts linear paths (middle). It then reconciles the graph with reads (dashed colored arrows, bottom) and pairs (not shown), and outputs one linear sequence for each splice form and/or paralogous transcript reflected in the graph (bottom, colored sequences).

图4.4 Trinity拼接原理图示

拼接得到的转录本序列信息以FASTA格式储存。

```
>TRINITY_DN162_c0_g1_i1 len=1849 path=[0:0-1848]
GACGCCTACCTGACTTGATCCAGGCCGCGGAGTGCATCTCCATAGACAGCCGGAAGGGACGGGCAAAGACTTGGCTCCTA
CAGCAGCCTCATTGTAGCTTTCGTCAACCCTCTCCATCACCAGCCTCGATCCCCCGCCACAGGAACCCGTCCCAGCTCAT
TAGAAGATGAGTGACCCAGAGTCAGATTTCAACCTTAAAGTCGTCCTGGTCAGCTTCAAGCAGTGTCTTACTGAAGAGGG
GCAAGTGCTGCTGGACCACTACATTACCAGCTGGAAGGGGCTAGTCAGGTTTCTGAACAACCTGGGTGCCGTATTCTCAT
TCATCTCCAAGGACGTGGTCTCAAAGCTGCAGATAATGGAAAGTCTGAGGAACAGCCCGCAGTGTGAACACTACACCAGC
TTACAGTCCATGGTCGCCTACGAGGTGGGCAACAAGCTGATAGACCTAGATCGCCGCTCCCGGCCCCGATACCCTAACTC
AGGCTGCCGGACTGTGCTGCGCCTGCACCGTGCTTTGCGCTGGCTGCAGCTGTTCCTCGAAGGCCTACGCACCAGCCCCG
AGGATGCTCGTACCTCCACGCTCTGCAGTGATGCCTATAATGCCACACTGGCTGCCTATCACTCCTGGATTGTACGTCAA
GCTGTCACTGTGGCCTTCTGCGCCCTGCCCACACGCAAGGTCTTCCTCGAGGCCATGAATGTGGGCTCCTCTGAGCAATC
AGTGGAGATGCTGGGAGAGGCCCTACCCTTCATCGAGAATGTGTATGACATCTCCCAGAAGCTCTTTGCTGAGCACTCCC
TGCTAGACCTGCCCTAAGGGTGCTTCCCCAACTCAGAACTGGGCTTGGGCTACCTGGGACCTCTCCCGCAGCAGTGCTTT
CTGGATGCTGCTGTCCCCCTGAAAGCTGGGCTTGTAGAGGGCCATGGGCTGTGAAGAGAAAGGTGGCAGCAGCTGAACCA
AGTGAGGTGGGTGGTAGCCGTGCTCTTGGTCCAGGACAAGACCATTGAACTCCATTTTCGCCCTCTGCTTCAGTTATGTG
TGTGTTCAGTCAGGCCTTATTGAAATTAGATGGCACATAGAGATGATGGGGGAGGAGGGCCATGTTCTACCTCCCATGTG
TGGAAACTGAAACGTTTTAAGGGCACAGGTCCCTGGAACTGAGGTTCTGTCTCCCCACAGTCAACATGCCCAGTCAGTGC
ATATCCTTCCTGTGCCCATCTTGAACCTAAGACATGGCATCAGTGCCAGATAAACAGCCCTGGGCCTACATGCCTGGGTC
CCCTTCATATTCCAGAAGCTGTCCTCGGGTAAGATGAGGCTCCTTTGGCAGTGACACGAATATCGAAGACCCTTTTCCTT
GCCCTTCACCTGCCTCCTTGTTAGCCTCTCTGCGTATCAAACAGGCCCTTGTCTCCTTGGAAATGGAGACCATTAAAACT
GCTAGCTGAGACTTCCTCAGAAAAGCGTGCTCTGGTCAGTGCAGCCTGCTTCACAGCTTCTGCAATACAGTATCAGCCTT
CAGAAGGTGGCCTGCCCCAAGACTCTAAAATAAAATATCAATACTAATTGGCCAAACCACGCCTCTCTCTAGCCAGCTTG
```

1 项目简介
2 建库测序流程
3 信息分析流程
4 分析结果
5 Appendix
6 参考文献

```
GGGTATGGC
>TRINITY_DN121_c0_g1_i1 len=328 path=[0:0-327]
GAGAGTTCTATATCCAAATGTGCAGGCAGCAGAAAGAGAAAGAAAGATATGAAACCTCAGAGTGCTCCCCTAGTGACATA
CTTCCTTCAGAAAGGAACACGCCTACTCCAACAAAGTCACACCTAATATATGCCTATGGGGGCCTTTCTTATCAAACCAC
CACACCACCCCTTCCCCAACACATTCCTATATAATTTACTAAAGATGTAACTGGGAACAGTGTGTCATTCCTTGAGAAAA
ACAATGTTCATCATTGGCTTTACACTCTTCATTCTTTAGTGCAGTGTTTTGTGAGGCAACTATGAGCTGCCTAACCTTCT
AACTTGAC
```

其中大于号>后紧跟转录本的id号，len=后面为转录本的长度，即该转录本的碱基数，path为从 de Bruijn Graph subComponent中经历的路径。其后为该转录本的碱基序列。每个转录本的id号构成都为c_g_i, 其中c为拼接过程形成的de Bruijn Graph Component, g为subcomponet，可以看作为广泛意义上的gene，i 代表转录本isoform。即：c_g为基因ID，c_g_i为c_g基因不同的转录本。详细解释见Trinity官方网站http://trinityrnaseq.github.io..

结果目录：Result/2.Assembly/1.AssembledTranscriptome/Trinity.fasta

4.2.2 Corset层次聚类

Corset(Davidson NM et al., 2014)，在Trinity拼接基础上，根据转录本间Shared Reads将转录本聚合为许多cluster，再结合不同样本间的转录本表达水平及H-Cluster算法，将样本间有表达差异的转录本从原cluster分离，建立新的cluster，最终每个cluster被定义为“Gene”。该方法可以聚合冗余转录本，并提高差异表达基因的检出率，Corset官方网站https://code.google.com/p/corset-project。

去除冗余之后的序列仍以FASTA格式存储。

```
>Cluster-4432.15961
GCCCGTCCCCCTGACCCCAGGGTCTGAGTCGCCGCTGCCGCTGCTGCCACCGTCCGCGAGGCAGGCGATAAGGGGAAGAG
TGCAGCGCGGCGGCCCCGGGGCCGAGGAGGGACCCAGATATGGAGCTGCGGGAAGAAGCTTGGTCTCCGGGGCCGCTGGA
TTCTGAGGATCAGCAGATGGCCAGTCATGAGAACCCAGTGGACATCCTCATCATGGATGACGACGACGTCCCCAGCTGGC
CCCCGACCAAGCTGTCCCCACCCCAGTCGGCGCCTCCGCCTGGGCCCCCGCCTCGGCCGCGGCCTCCCGCACCCTACATC
TGCAACGAGTGCGGCAAGAGCTTCAGTCACTGGTCGAAGCTGACCCGACACCAGCGCACGCACACAGGCGAGCGGCCCAA
CGCCTGTAGCGACTGCGGCAAGACCTTCTCCCAGAGCTCGCACCTGGTGCAGCATCGGCGCATCCACACAGGCGAGAAGC
CGTATGCCTGCTCTGAGTGCGGCAAGCGCTTCAGTTGGAGCTCCAACCTTATGCAGCACCAGCGCATCCACACGGGCGAG
AAGCCCTACACCTGCCCCGACTGCGGCCGCAGCTTCACACAGAGCAAGAGCCTGGCCAAGCACCGGCGCTCACACAGTGG
CCTCAAGCCCTTCGTGTGCCCGCGATGCGGCCGCGGCTTCAGCCAGCCCAAGAGCCTGGCGCGCCACCTACGGCTGCACC
CAGAGCTGTCAGGGCCGGGTGTGGCGGCCAAGGTGCTGGCCGCCAGCGTGCGGCGCGCCAAAGCGCCCGAGGAGGCTGCG
GCGGTGGACGGCGAGATCGCCATCCCAGTGGGTGACGGCGAGGGCATCATCGTGGTGGGCGCACCGGGAGACGGTGCTGC
AGCAGCCGCAGCCCTGGCCGGAGCAGGGCAGCGGGCACCGGGGCCGCGGTCGCGGAGGGCTCCGGCGCCCAAGCCATACG
TGTGCATGGAGTGCGGGAAGGGCTTTGGGCATGGGGCCGGGCTGTTGGCCCACCAGTGCGCACAGCACGGGGACGGGCTG
GGAGCAGCGGGTGGCGAGGAGCCTGCACACATCTGCGTGGAGTGTGGCGAGGGCTTCGTGCAGGGCGCTGCCCTGCGGAG
ACACAAGAAGATCCACGTGGTTGGGGCGCCTTCGGTCTGCAGCAGCTGCGGACAGAGTTTCTATCGGGCAGGTGGGGAGG
ACGACGGCGAGGATCAGTCGGCCGGTGCGCGGTGTGCCGAGTGTCGTGGTGGGGAGGCCCGGTAGGGACGGGGGTGCCCG
AGGGGTGGGCAGGGCCCCTCGGGCTCAGTGGGGACTATCAGCGAGCAAGACCACGAGGCCCAGGGAGAACCGGACCGGGA
GGATCAGCAGCTTCGCTCCTCCCTTGGCCCACGCTGACCCGGGTGTGAGGGGTCCCGCAGTCATCGGGGCAGGTTCTCAG
GACTGCCCACAGCCTCTCTGCTGACTGGCACCGGCCCTGTGCAGGTTCCCACCTTGGGAACCAGAGACTATGTGAGAGAA
CTGTGTAGATCAATCGGCACAAGACCCTCAATAGAGAGGCCATCCCCCAGTGCAGGTGTGGGGCTGCGGTGTCCTAAGGA
ATGGAGGGATCCTGGCCTTGTGCACCAAGAGGGTCTTTCTTCCCTTCTAGAATTTTCTTTTAATCATACCTTTTTGGCCC
CTCCCGTTCATTAATTTTTCTGTCCCATGGGTCCTTGTCGATTTTCTAGCCCAAGTCGTTCCTCTCCCTCCTGGATTTGC
AGTAGCAGAGGAGTGAAGAAGTAGCCTCCTGTTCCCATCACGGTCTCATTGAGAGGACAGAGTCCTGTGTGCTCCCTGTC
CTGACGGTGAAGAAGGGATGTGGGGGCAATAAATGGCACTATTCAATTGAAAAAAAA
>Cluster-4432.15960
GAGTTCCGGAAGAAAGATGGCGGCGGCCTGAGTCTAGGTCCGCGTAAGACTTTGCGGGCGCACAGTAGCGCTCCCTAGAC
CCGGCGCTCCAGAAAGCCGGGGGTGAGTGAGGGGTCTCTGAAGCTGCTTTGTGAGGGGCGGCCTTGGAAGGCCACTCATC
CACCGTTCGGGTCGCTTTGGGTCTGATCCCGCTTGACCCCGCGGTCGCCCTGTTAAGAGTGCGCCCGCTCTCTTGCCCGG
GTGCTTATTCCGGAACTCACGGAGCCGCGGGGGACTGAGGCTCGGACTGTGTTACGGGCTGGCGTTGTGACCTCAGCTTT
GTTCCAGCATCTCTATCCCGGTTTTGTCTTCGGAGTCCCATGGTCTGACTGGTGGCAATGGCGTCCAGGCAGAGTTCCCT
CTGTGGTCTGGCTGCTCATGGCCTCTGGGTCTTGGGCCTTGTCCTCCTGATGGATGCAGCTGCTAGACCAGCCAACCACT
CATCTACCCGGGAGAGAGCGACCAACAGGGAGGAAAATGAGATCTTACCCCCAGACCATTTGAATGGGGTGAAACTGGAG
ATGGATGGACACCTGAACAAGGACTTCCACCAGGAAGTTTTCCTGGGGAAGGACATGGATGGGTTTGATGAGGACTCAGA
GCCAAGAAGAAGCCGGAGGAAGCTGATGGTCATCTTTTCTAAGGTAGATGTGAACACTGACCGGAGGATCAGCGCTAAGG
AGATGCAGCACTGGATTATGGAGAAAACAGCGGAGCACTTCCAGGAGGCTGTGAAGGAAAACAAACTACACTTTAGGGCT
GTGGACCCCGATGGTGACGGCCGTGTGTCCTGGGATGAATATAAAGTGAAGTTTTTGGCAAGCAAAGGCCACAATGAGAA
GGAAATTGCTGAGGCCATCAGGAACCATGAGGAGCTCAAAGTGGATGAGGAGACACAGGAAGTCCTTGAGAACCTCAAAG
GTCGCTGGTACCAGGCAGACAATCCACCTGCAGACCTGCTGCTGACTGAGGATGAATTCCTTTCATTCCTTCACCCTGAG
CACAGCCGGGGCATGCTTAAGTTCATGGTCAAGGAGATTGTTCGGGACTTGGACCAGGATGGAGACAAGCAGCTGTCTCT
ACCTGAATTCATCTCCTTGCCTGTGGGCACTGTTGAAAATCAGCAAGGCCAAGACATTGATGACAATTGGGTGAAAGACA
GGAAGAAAGAGTTTGAGGAACTGATTGATTCCAACCATGATGGGATTGTGACCATGGAGGAGCTGGAGAATTACATGGAC
CCCATGAATGAATACAATGCCCTCAATGAAGCCAAACAGATGATCGCCATTGCTGATGAGAACCAGAACCACCACCTGGA
ACCTGAGGAGATCCTCAAGTATAGCGAGTTCTTCACTGGCAGCAAGCTGATGGACTATGCCCGCAATGTGCATGAGGAGT
TCTGAGCATGCCCCTCAGCCAGAGCCAGGCCGCCAGTCTCCTGTCTGACCCTTCCTACACCTCGAGGGGCCTGTGGTCTA
GTGAGACCTGTGGTTGACTGGGTCCCAGGGTGGGTCTGAAATGCTACCTACTCTGTATCTGGGGGCTACCATGCCCCCTA
AAAGTGCTGTCCCAGAACCTCCTTTCTTCTGCTGTGTCCATATGCTCTGGCTCAGCTTCTGCCACAGCATGGCCCAGACT
TATTAAAGATATGCTTACTAGGCTATGTGTCCTCTCCAGCATGTGTCTGAGGCAGCGGCCCTTCTGTACCAATCATGGGC
TGAGCTGCATTGGCTTGCTAAATCTCTCTCAATTGCTATATTCTACTTCTGAGAATTGCTTGGAGTGGTTGGTTCAGTTG
AGGGTGCCTCACACGTCAGATCCAAGAGCTCTGGGCTGGGCCCCTCCGTGGGCTCACCACACTGGGGGAATAAATGCAAC
```

1 项目简介
2 建库测序流程
3 信息分析流程
4 分析结果
5 Appendix
6 参考文献

其中大于号>后紧跟序列id号，Clusters-X.Y,X为super_cluster ID:转录本有相同reads比对上的会有相同的super_cluster ID,Y为tsuper_cluster ID中的cluster number。

结果目录：Result/2.Assembly/1.AssembledTranscriptome/unigene.fasta

4.2.3 拼接转录本长度分布

将 Trinity 拼接得到的转录本序列，作为后续分析的参考序列。以Corset层次聚类后得到最长Cluster序列进行后续的分析。对转录本及聚类序列长度分别进行统计，结果如下所示。

表4.3 拼接长度频数分布情况一览表

Transcript Length Interval	200bp-500bp	500bp-1kbp	1k-2kbp	>2kbp	Total
Number of transcripts	14181	11337	10333	14113	49964
Number of Unigenes	10425	7981	5518	6953	30877

显示注释

表4.4 拼接长度分布情况一览表

Type	min length	mean length	median length	max length	N50	N90	total nucleotides
Transcript	301	1638	965	15983	2852	644	81849060
Unigene	301	1405	726	15983	2536	515	43388299

显示注释

拼接转录本与基因序列长度分布情况如下图所示:

Search..
Length_distribution
Transcript_Length_distribution
Unigene_Length_distribution

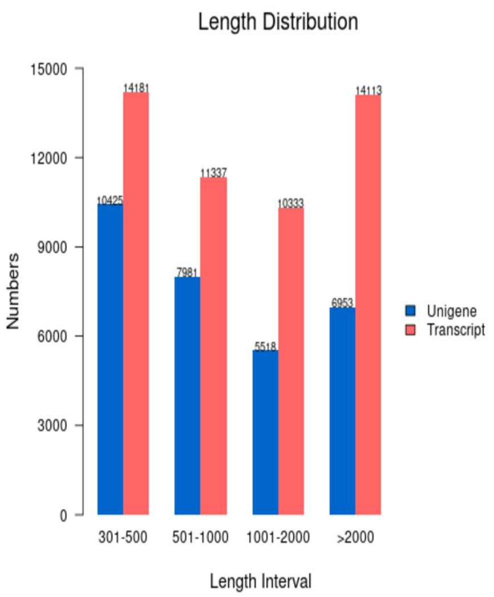

图4.5 基因转录本/基因序列长度分布图

注：图中横坐标为转录本/基因长度/长度间隔，纵坐标为转录本/基因的数量

结果目录：Result/2.Assembly/2.AssemblyINFO

4.2.4 转录本质量评估

BUSCO（Benchmarking Universal Single-Copy Orthologs）评估是使用单拷贝直系同源基因库，结合tblastn、augustus和hmmer等软件对组装得到的转录本进行评估，以此评估转录本组装的完整性。我们采用BUSCO软件对拼接得到的Trinity.fasta，unigene.fa和cluster.fasta进行拼接质量的评估,根据比对上的比例、完整性，来评价拼接结果的准确性和完

1 项目简介
2 建库测序流程
3 信息分析流程
4 分析结果
5 Appendix
6 参考文献

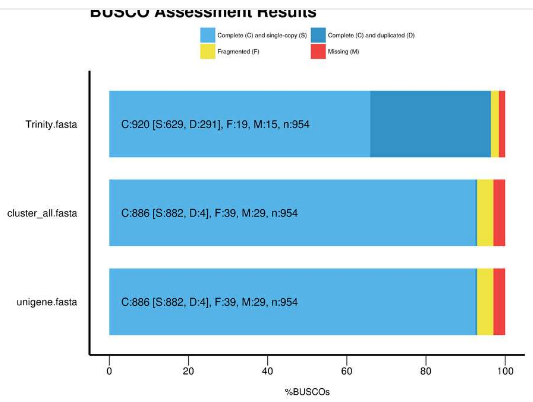

图4.6 拼接转录本BUSCO评估结果（不同的颜色分别代表不同的拼接转录本类型）

显示注释

结果目录：Result/2.Assembly/3.AssemblyAssesment

转录本拼接讲解视频：链接: https://pan.baidu.com/s/1ZYxK6UB0ByTIgcjYykQqCQ 提取码: retj

显示QA

4.3 常用功能数据库注释

4.3.1 功能注释数据库介绍

得到拼接转录本后，需要对转录本进行功能注释，以获得gene的功能信息。为获得全面的基因功能信息，我们进行了七大数据库的基因功能注释，包括：Nr，Nt，Pfam(Finn RD et al., 2008)，KOG/COG，Swiss-prot，KEGG，GO。这七种数据库的特点，网址及我们注释使用的方法和参数见下表：

表4.5 七种数据库介绍及使用的软件参数

数据库	简介	
Nr	NCBI non-redundant protein sequences，NCBI官方的蛋白序列数据库	
Nt	NCBI nucleotide sequences，NCBI官方的核酸序列数据库	
Pfam	Protein family，最全面的蛋白结构域注释的分类系统	
KOG/COG	COG: Clusters of Orthologous Groups of proteins KOG: euKaryotic Ortholog Groups	
Swiss-Prot	A manually annotated and reviewed protein sequence database	
KEGG	Kyoto Encyclopedia of Genes and Genomes	系统分析基因产物
GO	Gene Ontology，一套国际标准化的基因功能描述的分类系统	

为方便了解本项目的注释率情况，现统计本项目数据在七大数据库中的注释成功率情况，见表4.6基因注释成功率统计。NR，GO，KOG，KO注释部分结果展示见下小节。其它数据库比对注释结果详见结果文件。

表4.6 基因注释成功率统计

Database	Number of Unigenes	Percentage (%)
Annotated in NR	18230	59.04
Annotated in NT	29623	95.93
Annotated in KO	13612	44.08
Annotated in SwissProt	16028	51.9
Annotated in PFAM	12792	41.42
Annotated in GO	12792	41.42
Annotated in KOG	7114	23.03
Annotated in all Databases	5359	17.35
Annotated in at least one Database	29740	96.31
Total Unigenes	30877	100

1 项目简介
2 建库测序流程
3 信息分析流程
4 分析结果
5 Appendix
6 参考文献

在7大数据库注释结果中选出5个数据库绘制的venn图，结果如下：

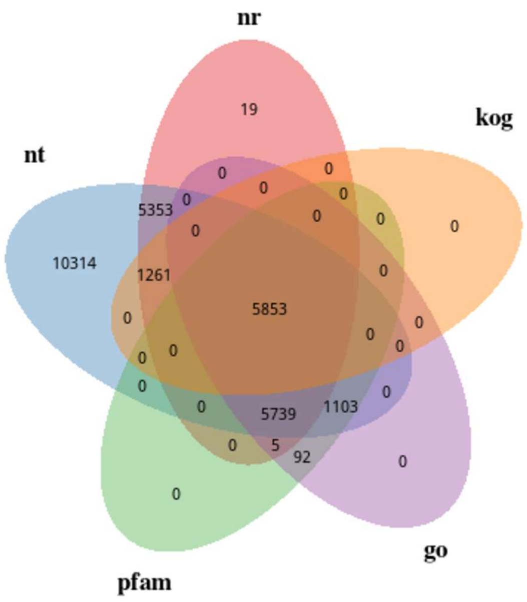

图4.7 注释结果韦恩图

注：不同的颜色表示不同的数据库

结果目录：Result/3.Annotation/1.GeneFunctionalAnnotation

4.3.2 Nr注释结果

通过与Nr库进行比对注释，可以获取本物种基因序列与近缘物种基因序列的相似性以及本物种基因的功能信息。Nr注释部分结果示意见下表。

表4.7 Nr注释结果

Genes	Gene Length	NR GI	NR ID	NR Score	NR Evalue	
Cluster-4432.15961	1897	XP_048282775.1	XP_048282775	990	6.60e-126	
Cluster-4432.15960	2125	XP_048273415.1	XP_048273415	1821	5.06e-251	45 kDa calcium
Cluster-4432.15963	1379	XP_048285238.1	XP_048285238	856	2.61e-111	
Cluster-4432.15962	1527	XP_005371501.1	XP_005371501	2298	0.0	
Cluster-4432.15965	1741	XP_040610654.1	XP_040610654	2593	0.0	

显示注释

通过Nr库比对注释的结果，可以统计并绘制比对上的物种分布图、比对的e-value分布图和序列相似度分布图，见下图Nr统计图。

1 项目简介
2 建库测序流程
3 信息分析流程
4 分析结果
5 Appendix
6 参考文献

Species_Classification
E-value_Distribution
Similarity_Distribution

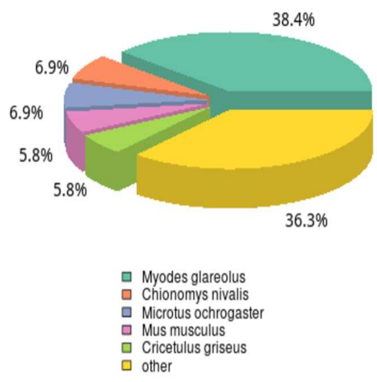

图4.8 NR 库比对统计图

注：Species_classification为Nr库比对上的物种分布图（拉丁名过长时用"…"省略）；Evalue_Distribution为比对上的e-value分布图; Similarity_Distribution比对上的相似度分布图

结果目录：Result/3.Annotation/1.GeneFunctionalAnnotation

4.3.3 GO 注释

对基因进行GO注释之后，将注释成功的基因按照GO三个大类(BP Biological process, CC Cellular component, MF Molecular Function)的下一层级进行分类， 如下图所示。

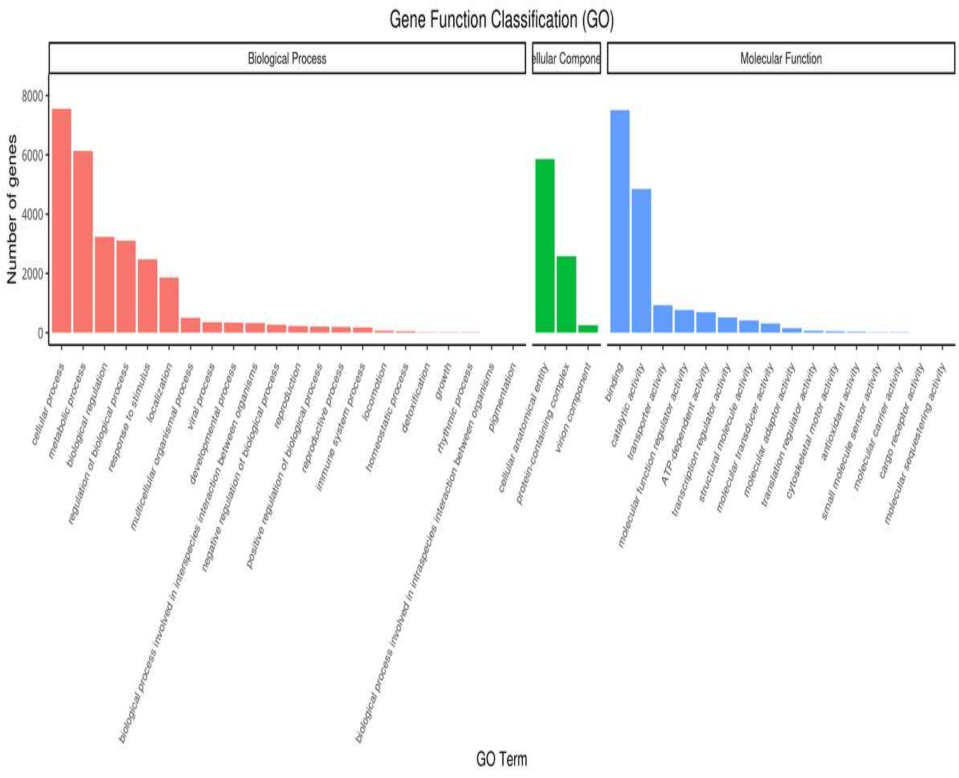

图4.9 GO注释分类统计图

注：图中横坐标为GO Term，纵坐标为注释到该GO Term的基因数量

结果目录：Result/3.Annotation/2.GOclassification

KOG 分为以下group，将 KOG 注释成功的基因按 KOG 的 group 进行分类，如下图所示。

1 项目简介
2 建库测序流程
3 信息分析流程
4 分析结果
5 Appendix
6 参考文献

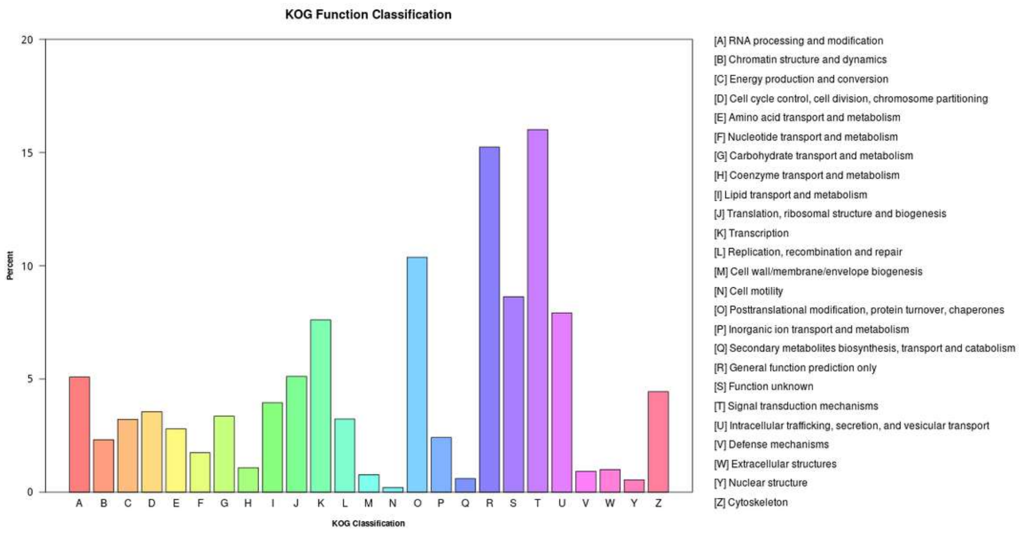

图4.10 KOG 注释分类统计图

注：图中横坐标为KOG的group的名称，纵坐标为注释到该group下的基因个数占被注释上的基因总数的比例

结果目录：Result/3.Annotation/3.KOGclassification

4.3.5 KEGG注释

对基因做KO注释后，可根据它们参与的KEGG代谢通路进行分类，如下图所示。

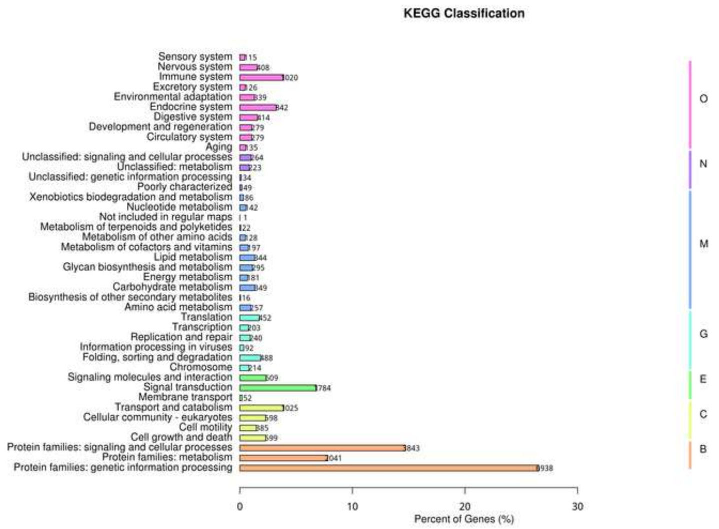

图4.11 KEGG 代谢通路分类统计图

注：图中纵坐标为KEGG代谢通路的名称，横坐标为注释到该通路下的基因个数及其个数占被注释上的基因总数的比例。 将基因根据参与的KEGG代谢通路分为5个分支：细胞过程(C，Cellular Processes)，环境信息处理(E，Environmental Information Processing)，遗传信息处理(G，Genetic Information Processing)，代谢(M，Metabolism)，有机系统(O，Organismal Systems)。以及(B，Brite Hierarchies)和不包括在Pathway或Brite的通路(N，Not Included in Pathway or Brite)

结果目录：Result/3.Annotation/4.KEGGclassification

常用功能数据库注释讲解视频：链接: https://pan.baidu.com/s/1vDdFVIKpgabIKHDYJ1EyEg 提取码: ifs4

显示QA

CDS是编码一段蛋白产物的序列。预测基因CDS后，根据需要将核酸序列翻译成氨基酸序列，从而可以用软件预测它的蛋白质结构，推测它的功能或者蛋白类型，另外还可以用来辅助设计qPCR引物或构建蛋白序列库。

1 项目简介
2 建库测序流程
3 信息分析流程
4 分析结果
5 Appendix
6 参考文献

CDS预测有两种常见的方法：一是基于同源序列预测，选取阅读框；二是基于一定的模型预测。

4.4.1 CDS预测

CDS预测分为两步： 1. 将Gene按照NR蛋白库，Swissprot蛋白库的优先级顺序进行比对，若比对上，则从比对结果中提取出转录本的ORF编码框信息，并按照标准密码子表将编码区序列翻译成氨基酸序列(按照5'->3'的顺序)； 2. 对于没有比对上NR蛋白库、Swissprot蛋白库的序列，或者比对上未预测出结果的序列，则采用TransDecoder(3.0.1)软件预测其ORF，从而得到这部分基因编码的核酸序列和氨基酸序列。

```
>Cluster-4432.15961;orf1 len=1194 frame:3 start:72 end:1265 zinc finger protein 787 isoform
X2 [Myodes glareolus]
GGGGAAGAGTGCAGCGCGGCGGCCCCGGGGCCGAGGAGGGACCCAGATATGGAGCTGCGG
GAAGAAGCTTGGTCTCCGGGGCCGCTGGATTCTGAGGATCAGCAGATGGCCAGTCATGAG
AACCCAGTGGACATCCTCATCATGGATGACGACGACGTCCCCAGCTGGCCCCCGACCAAG
CTGTCCCCACCCCAGTCGGCGCCTCCGCCTGGGCCCCCGCCTCGGCCGCGGCCTCCCGCA
CCCTACATCTGCAACGAGTGCGGCAAGAGCTTCAGTCACTGGTCGAAGCTGACCCGACAC
CAGCGCACGCACACAGGCGAGCGGCCCAACGCCTGTAGCGACTGCGGCAAGACCTTCTCC
CAGAGCTCGCACCTGGTGCAGCATCGGCGCATCCACACAGGCGAGAAGCCGTATGCCTGC
TCTGAGTGCGGCAAGCGCTTCAGTTGGAGCTCCAACCTTATGCAGCACCAGCGCATCCAC
ACGGGCGAGAAGCCCTACACCTGCCCCGACTGCGGCCGCAGCTTCACACAGAGCAAGAGC
CTGGCCAAGCACCGGCGCTCACACAGTGGCCTCAAGCCCTTCGTGTGCCCGCGATGCGGC
CGCGGCTTCAGCCAGCCCAAGAGCCTGGCGCGCCACCTACGGCTGCACCCAGAGCTGTCA
GGGCCGGGTGTGGCGGCCAAGGTGCTGGCCGCCAGCGTGCGGCGCGCCAAAGCGCCCGAG
GAGGCTGCGGCGGTGGACGGCGAGATCGCCATCCCAGTGGGTGACGGCGAGGGCATCATC
GTGGTGGGCGCACCGGGAGACGGTGCTGCAGCAGCCGCAGCCCTGGCCGGAGCAGGGCAG
CGGGCACCGGGGCCGCGGTCGCGGAGGGCTCCGGCGCCCAAGCCATACGTGTGCATGGAG
TGCGGGAAGGGCTTTGGGCATGGGGCCGGGCTGTTGGCCCACCAGTGCGCACAGCACGGG
GACGGGCTGGGAGCAGCGGGTGGCGAGGAGCCTGCACACATCTGCGTGGAGTGTGGCGAG
GGCTTCGTGCAGGGCGCTGCCCTGCGGAGACACAAGAAGATCCACGTGGTTGGGGCGCCT
TCGGTCTGCAGCAGCTGCGGACAGAGTTTCTATCGGGCAGGTGGGGAGGACGACGGCGAG
GATCAGTCGGCCGGTGCGCGGTGTGCCGAGTGTCGTGGTGGGGAGGCCCGGTAG
>Cluster-4432.15960;orf1 len=1077 frame:3 start:369 end:1445 45 kDa calcium-binding protein
[Myodes glareolus] >XP_048273423.1 45 kDa calcium-binding protein [Myodes glareolus]
>XP_048273429.1 45 kDa calcium-binding protein [Myodes glareolus]
CTGGTGGCAATGGCGTCCAGGCAGAGTTCCCTCTGTGGTCTGGCTGCTCATGGCCTCTGG
GTCTTGGGCCTTGTCCTCCTGATGGATGCAGCTGCTAGACCAGCCAACCACTCATCTACC
CGGGAGAGAGCGACCAACAGGGAGGAAAATGAGATCTTACCCCCAGACCATTTGAATGGG
GTGAAACTGGAGATGGATGGACACCTGAACAAGGACTTCCACCAGGAAGTTTTCCTGGGG
AAGGACATGGATGGGTTTGATGAGGACTCAGAGCCAAGAAGAAGCCGGAGGAAGCTGATG
GTCATCTTTTCTAAGGTAGATGTGAACACTGACCGGAGGATCAGCGCTAAGGAGATGCAG
CACTGGATTATGGAGAAAACAGCGGAGCACTTCCAGGAGGCTGTGAAGGAAAACAAACTA
CACTTTAGGGCTGTGGACCCCGATGGTGACGGCCGTGTGTCCTGGGATGAATATAAAGTG
AAGTTTTTGGCAAGCAAAGGCCACAATGAGAAGGAAATTGCTGAGGCCATCAGGAACCAT
GAGGAGCTCAAAGTGGATGAGGAGACACAGGAAGTCCTTGAGAACCTCAAAGGTCGCTGG
TACCAGGCAGACAATCCACCTGCAGACCTGCTGCTGACTGAGGATGAATTCCTTTCATTC
CTTCACCCTGAGCACAGCCGGGGCATGCTTAAGTTCATGGTCAAGGAGATTGTTCGGGAC
TTGGACCAGGATGGAGACAAGCAGCTGTCTCTACCTGAATTCATCTCCTTGCCTGTGGGC
ACTGTTGAAAATCAGCAAGGCCAAGACATTGATGACAATTGGGTGAAAGACAGGAAGAAA
GAGTTTGAGGAACTGATTGATTCCAACCATGATGGGATTGTGACCATGGAGGAGCTGGAG
AATTACATGGACCCCATGAATGAATACAATGCCCTCAATGAAGCCAAACAGATGATCGCC
ATTGCTGATGAGAACCAGAACCACCACCTGGAACCTGAGGAGATCCTCAAGTATAGCGAG
TTCTTCACTGGCAGCAAGCTGATGGACTATGCCCGCAATGTGCATGAGGAGTTCTGA
```

>Cluster-X.Y: 序列的ID，是这条基因的唯一识别符

orf1: 预测ORF的id，有的基因有两个及以上的预测的ORF

len: 该ORF的碱基的长度

frame: 该ORF在原来基因上的读码框，-代表负链

start: 该ORF在原来基因上的起始位置

end: 该ORF在原来基因上的终止位置

gi|***|ref|XP_***|: 比对到蛋白库的基因的描述

通过TransDecoder软件预测的CDS的结果展示：

1 项目简介
2 建库测序流程
3 信息分析流程
4 分析结果
5 Appendix
6 参考文献

```
len:115 (+) Cluster-1004.0:1-345(+)
GCCACTCTGTGGGCACTCCCTTCTCAGACTAGAGCAGAGTGTAGACAGTATATGACCCAG
GAGCACCCCAATCAGAGTGTCTGCCACAGCTGCTGGGCCTCAGCAAGCGTCGTCTCTCTA
AGGAACAGCTGCTGTTGTAAGCTTGGTGGCCTCGCCACTGTGCTTGGTCCTACTGTCCAC
ACCCCAGGCTCTTGTGGTTGTGGGAAGTGTGGCTGCAGTAGGATGATGGTACTCTCTGCT
CCTGGACTCTCTGTGGTAAGAAAGGTGCCAGGTCTGTGGACCACCACAAGCCCACTGAGG
CCTCGGAGGGGCACCTGTCACAGTCAGCTCCTAGCCTGTGCCTAA
>Gene.931::Cluster-105.0::g.931::m.931 Gene.931::Cluster-105.0::g.931 ORF
type:5prime_partial len:113 (-) Cluster-105.0:20-358(-)
CTTCTAACTTACTTTTCTCTGCTAAATTTCTGGGCTTATTCTGGGGGCCAGTTTAGAGTT
GTATTTGCCGTGTTAATCCAGTTCCTCAGAGTTCTTTTACAGATCCCAGTCAGTGTCTTC
GCTCCTGGTCACACTTCACCTTTTACTCTGTGTGCCCCTTCTGACCTCACAATCCTTCAG
CTGCAGCCTCAGGCCACACCCCTCCAGATGCTGACCATCCCTAGCTGCCTAAAGCACTCT
CTTGTCTGCCCGGTCTGGGCCTTTCTTCACCTTATGATAAACACCTGCCCCTTTGGTCAT
CCAACCCTATGGGCACTCTCAGGTGCCCGGGGCCAGTAG
```

>Cluster-X.Y: 序列的ID，是这条基因的唯一识别符

orf1: 预测ORF的id，有的基因有两个及以上的预测的ORF

len: 该ORF的碱基的长度

start: 该ORF在原来基因上的起始位置

end: 该ORF在原来基因上的终止位置

minus strand: 代表该ORF在原来转录本上是负链，如果没有这个信息，就是正链

结果目录：Result/4.CDSprediction

CDS 预测讲解视频：链接: https://pan.baidu.com/s/1JRX79Li3ql59gVZEnBoj0g 提取码: hpik

显示QA

4.5 基因转录因子

植物转录因子预测使用iTAK软件，其基本原理是，利用database中分类定义好的TF(transcription factor) family及规则，通过hmmscan鉴定TF。TF的鉴定和分类方法为PlnTFDB(Perez-Rodriguez P et al., 2010)和PlantTFDB(Jin J et al., 2014)

动物转录因子鉴定使用动物转录因子数据库——animalTFDB 2.0,根据转录因子家族的pfam文件，利用hmmsearch进行鉴定。基因转录因子分析结果如下。

表4.8 转录因子注释

GeneID	Family
Cluster-150.0	zf-C2H2
Cluster-1727.0	zf-C2H2
Cluster-1788.0	ZBTB
Cluster-1860.0	zf-C2H2

显示注释

结果目录：Result/3.Annotation/5.TransFactor

基因转录因子分析讲解视频：链接: https://pan.baidu.com/s/1HquIZ4H1Sr6koGCaHhKBdA 提取码: jne4

4.6 基因表达水平分析

4.6.1 参考序列比对

将 Trinity拼接得到的转录组作为参考序列(Ref)，将每个样品的clean reads往Ref上做mapping,分别过滤掉比对质量值低于10的reads，非成对比对上的reads，比对到基因组多个区域的reads。比对过程中我们采用了RSEM软件(Li B et al., 2011)，RSEM中使用到的bowtie2 参数mismatch 0,(bowtie2 默认参数)。比对统计结果见下表。

1 项目简介
2 建库测序流程
3 信息分析流程
4 分析结果
5 Appendix
6 参考文献

Sample Name	Total Reads	Total Mapped
CP1	22965776	18504334(80.57%)
CP2	20187538	15282026(75.70%)
CP3	19052836	14811841(77.74%)
DP1	21822533	17194412(78.79%)
DP3	23128172	17688057(76.48%)
DP2	26111713	20065441(76.84%)

此表仅对样本比对情况进行了部分展示，在网页版结题报告中，我们对该表格设置了Copy/Print/Download以及Search功能。

显示注释

结果目录：Result/5.Quant/1.Count

4.6.2 基因表达水平统计表

RSEM(Li B et al., 2011)对bowtie的比对结果进行统计， 比对到某基因的reads数目称为read count，我们对每个样本分别进行基因表达水平的定量分析，再合并得到所有样本的表达矩阵，第一列为基因的ID，其余列为各样本的原始read count值，如下表3.16所示。在RNA-seq技术中，FPKM(expected number of Fragments Per Kilobase of transcript sequence per Millions base pairs sequenced)是每百万fragments中来自某一基因每千碱基长度的fragments数目，其同时考虑了测序深度和基因长度对fragments计数的影响，是目前最为常用的基因表达水平估算方法(Trapnell C et al., 2010)。所以我们将readcount数进行了FPKM转换。结果(部分)见下表4.10。

表4.10 各样品read count统计结果

gene_id	CP1	CP2	CP3	DP1	DP3	DP2	Gene Length	NR GI
Cluster-4432.15961	44.00	101.00	76.00	94.00	112.00	85.00	1897	XP_048282775
Cluster-4432.15960	777.00	967.00	1029.00	1054.00	1227.00	1004.00	2125	XP_048273415
Cluster-4432.15963	2.00	48.00	32.00	24.00	62.00	39.00	1379	XP_048285238
Cluster-4432.15962	9.00	68.00	41.00	42.00	100.00	76.00	1527	XP_005371501
Cluster-4432.15965	2038.00	2916.00	2341.00	3504.00	4311.00	4031.00	1741	XP_040610654

注：此表仅对样本基因表达定量情况进行了部分展示，在网页版结题报告中，我们对该表格设置了Copy/Print/Download以及Search功能

显示注释

表4.11 各样品FPKM统计结果

gene_id	CP1	CP2	CP3	DP1	DP3	DP2	Gene Length	NR GI	
Cluster-4432.15961	1.51	4.17	3.24	3.45	3.99	2.67	1897	XP_048282775.1	XP_[illegible]
Cluster-4432.15960	23.28	34.92	38.39	33.80	38.26	27.56	2125	XP_048273415.1	XP_[illegible]
Cluster-4432.15963	0.10	2.94	2.03	1.31	3.28	1.82	1379	XP_048285238.1	XP_[illegible]
Cluster-4432.15962	0.40	3.66	2.28	2.01	4.65	3.11	1527	XP_005371501.1	XP_[illegible]
Cluster-4432.15965	77.55	133.57	110.82	142.51	170.50	140.30	1741	XP_040610654.1	XP_[illegible]

注：此表仅对样本基因表达定量情况进行了部分展示，在网页版结题报告中，我们对该表格设置了Copy/Print/Download以及Search功能

显示注释

结果目录：Result/5.Quant/1.Count

4.6.3 不同实验条件下，基因表达水平对比图

对不同实验条件下基因的FPKM做密度分布图及盒形图，进而能在整体水平上检查不同实验条件下FPKM分布的情况，结果见下图。

1 项目简介
2 建库测序流程
3 信息分析流程
4 分析结果
5 Appendix
6 参考文献

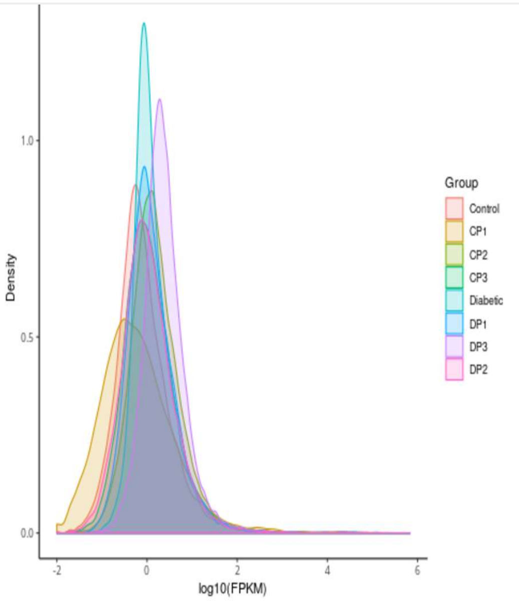

FPKM_density
FPKM_boxplot
FPKM_violin

图4.12 不同实验条件下样本基因的FPKM分布密度图/盒形图/小提琴图

图1：density:横坐标为基因的log10(FPKM)值，该数值越高，表示基因的表达量越高；纵坐标为对应log10(FPKM)的密度,不同颜色代表不同样品，该图从表达量的总体分布角度来衡量各样品之间的差异。

图2：boxplot:FPKM盒形图，横坐标为样品名称，纵坐标为log10(FPKM+1)每个区域的盒形图对五个统计量(至上而下分别为最大值，上四分位数，中值，下四分位数和最小值)，该图从表达量总体的离散角度来衡量各样品之间的差异。

图3：violin:FPKM小提琴图，横坐标为样品名称，纵坐标为log10(FPKM+1)。

结果目录：Result/5.Quant/2.Distribution

4.6.4 样品间相关性检查

样品间基因表达水平相关性是检验实验可靠性和样本选择是合理的重要指标。生物学重复是生物学实验所必须的，设置生物学重复主要有两个用途：一个是证明所涉及的生物学实验操作不是偶然，而是可重复的；另一个是为了确保后续的差异基因分析得到更可靠的结果。样品间基因表达水平相关性是检验实验可靠性和样本选择是否合理的重要指标。相关系数越接近1，表明样品之间表达模式的相似度越高。若样品中有生物学重复，通常生物重复间相关系数要求较高。Encode计划建议皮尔逊相关系数的平方（R2）大于0.92（理想的取样和实验条件下）（ENCODE Project Consortium, 2004）。具体的项目操作中，我们要求生物学重复样品间R2至少要大于0.8。根据样本所有基因的FPKM值计算组内及组间的相关系数，绘制成热图，可直观显示组间样本差异及组内样本重复情况。样本相关性热图如下图所示。

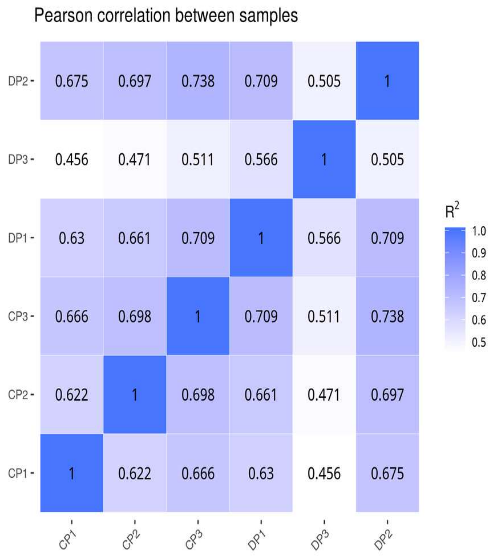

Search..
Pearson_correlation

注：横坐标为样品1的log10(FPKM+1), 纵坐标为样品2的log10(FPKM+1), R2:pearson相关系数的平方

1 项目简介
2 建库测序流程
3 信息分析流程
4 分析结果
5 Appendix
6 参考文献

结果目录：Result/5.Quant/3.Correlation

基因表达水平分析讲解视频：链接: https://pan.baidu.com/s/1hIlhxy3QZmILVxQa9frQpA 提取码: ucmp

4.6.5 共表达韦恩图

共表达维恩图展示了每个组/样本中唯一表达的基因数量，重叠区域展示了两个或更多组样本中共表达的基因数量。

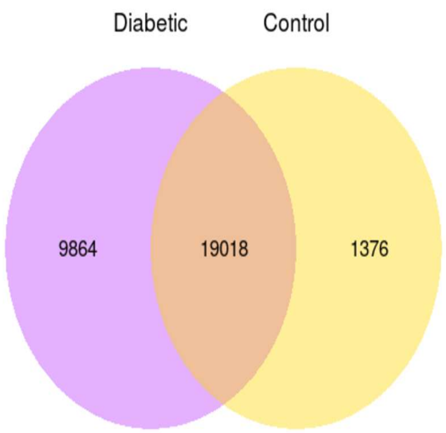

Search..
ControlvsDiabetic
CP3vsDP3
CP1vsDP1
CP1vsDP2
CP1vsDP3
CP2vsDP1
CP2vsDP2
CP2vsDP3
CP3vsDP1
CP3vsDP2

结果目录：Result/5.Quant/4.coExpression_venn

显示QA

4.7 差异分析

在转录组中，确定某个基因在不同的样品中的表达量是否有差异是分析的核心内容之一。获得基因表达量后，即可对表达数据进行统计学分析，进而筛选不同样本之间显著差异的基因。差异分析主要分为三个步骤:

首先对原始的read count进行标准化（normalization），主要是对测序深度的校正。然后通过统计学模型进行假设检验概率（P-value）的计算，最后进行多重假设检验校正（BH），得到FDR值（错误发现率，padj是其常见形式，以下皆使用padj代表FDR)。

针对不同的实验情况，我们选用合适的软件进行基因表达差异显著性分析，具体如下表所示。

表4.14 表达差异分析所用软件及差异基因筛选标准

类型	软件	标准化方法	pvalue计算模型	FDR计算方法	差异基因筛选标准
有生物学重复	DESeq2(Love MI et al., 2014)	DESeq2	负二项分布	BH	|log2(FoldChange)| >= 1& padj <= 0.05
无生物学重复	edgeR(Robinson et al., 2010)	TMM	泊松分布	BH	|log2(FoldChange)| >= 1& padj <= 0.005

若按照以上标准筛选得到的差异基因过少（低于100），很有可能导致后面的功能富集分析没有显著性结果，所以，我们会根据项目的具体情况，适当地降低筛选差异基因的阈值标准。若项目实验只关注某几个基因的表达情况（如基因敲除），不在意富集结果，从下面的差异分析表格中筛选关注的那几个基因即可。

1 项目简介
2 建库测序流程
3 信息分析流程
4 分析结果
5 Appendix
6 参考文献

据进行假设检验。而转录组分析是针对成千上万个基因进行的，这样会导致假阳性的累积，基因数目越多，假设检验的假阳性累积程度会越高，所以引入padj对假设检验的P-value进行校正，从而控制假阳性的比例。

4.7.1 差异基因列表

我们对每个比较组合进行差异显著性分析，最得到差异表达基因列表，如下表所示。对于有生物学重复的分组样本，我们利用read count平均值计算FoldChange值。

表4.15 差异基因列表结果（部分）展示

gene_id	DP1_readcount	CP1_readcount	log2FoldChange	pvalue
Cluster-4432.16102	0.0782181222491323	4938.59091655178	-15.8410470808231	4.72141215883104e-85
Cluster-4432.15564	1.01621969851235	2203.80988795734	-11.0742008987633	1.0646651614843e-65
Cluster-4432.15851	1.13908989842339	261.65974394851	-7.83622550188006	6.97199185013348e-42
Cluster-4432.16380	24.6972181274819	3791.34589268116	-7.26187442107881	3.52249851844955e-41
Cluster-4432.12638	5.88268027293376	955.877045743078	-7.3427663602179	4.69116953896855e-41

注：此表仅对样本差异基因情况进行了部分展示，在网页版结题报告中，我们对该表格设置了Copy/Print/Download以及Search功能。

显示注释

4.7.2 差异基因统计表

每个比较组合的差异基因（包括上调和下调）数目统计以及筛选差异的标准如下表所示：

表4.16 差异基因统计结果展示

compare	all	up	down	threshold
DP1vsCP1	6974	4285	2689	edgeR pvalue<=0.05 |log2FoldChange|>=1.0
DP2vsCP2	2876	1012	1864	edgeR pvalue<=0.05 |log2FoldChange|>=1.0
DP3vsCP3	5992	4292	1700	edgeR pvalue<=0.05 |log2FoldChange|>=1.0
DiabeticvsControl	1591	848	743	DESeq2 pvalue<=0.05 |log2FoldChange|>=1.0

显示注释

对每个比较组合的差异基因数（包含上调和下调）用柱状图进行展示：

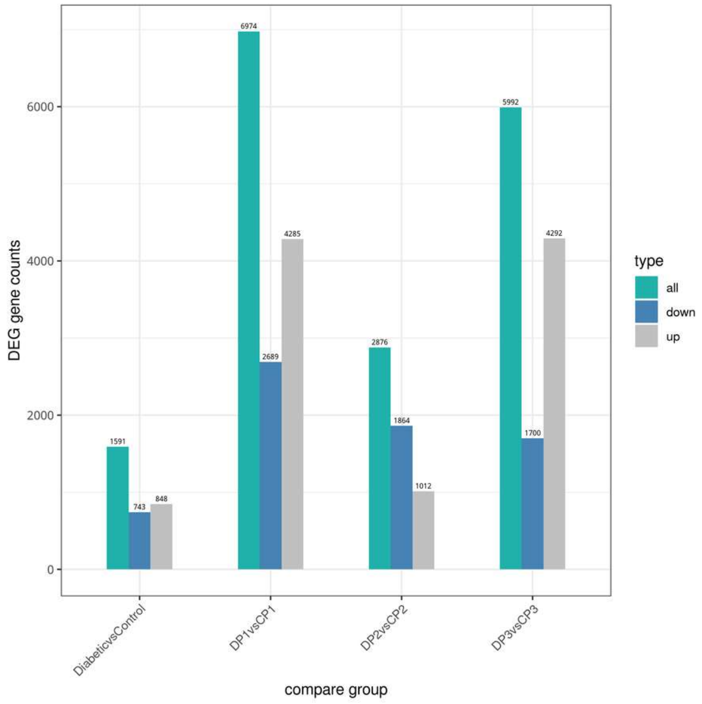

注：灰色和蓝色分别表示上调和下调的差异基因，柱子上的数字表示差异基因数目

1 项目简介
2 建库测序流程
3 信息分析流程
4 分析结果
5 Appendix
6 参考文献

4.7.3 差异基因火山图

火山图可直观显示表达差异显著性基因的整体分布情况，横坐标表示基因在不同样本中的表达倍数变化(log2FoldChange)，纵坐标表示表达差异的显著性水平(-log10padj或-log10pvalue)。若比较组合无表达差异显著性基因，默认调整筛选表达差异显著性的阈值进行火山图的绘制。上调基因用红色点表示，下调基因用绿色点表示，如下图所示。

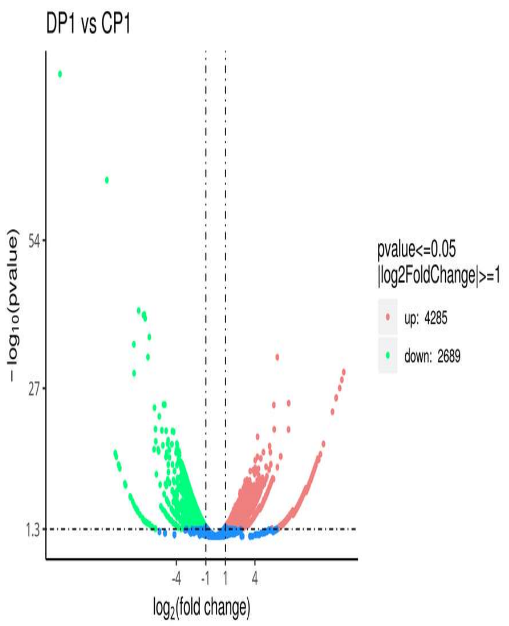

Search..
DP1vsCP1
DP2vsCP2
DP3vsCP3
DiabeticvsControl

图4.15 差异基因火山图

注：图中横坐标为log2FoldChange值，纵坐标为-log10padj或-log10pvalue，蓝色的虚线表示差异基因筛选标准的阈值线

结果目录：Result/6.Differential/1.deglist

4.7.5 差异基因聚类

将所有比较组的差异基因取并集之后作为差异基因集。两组以上的实验，可对差异基因集进行聚类分析，将表达模式相近的基因聚在一起。我们采用主流的层次聚类对基因的FPKM值进行聚类分析，对行（row）进行均一化处理（Z-score）。热图中表达模式相近的基因或样本会被聚集在一起，每个方格中的颜色反映的不是基因表达值，而是表达数据的行进行均一化处理后得到的数值（一般在-2到2之间），所以热图中的颜色只能横向比较（同一基因在不同样本中的表达情况），而不能纵向比较（同一样本不同基因的表达情况）。结果文件中既有组间的聚类，也有样品间的聚类。由于差异基因较多会导致聚类热图画失败，因此分析流程会按照基因在各个样本中的表达量加和从大到小排序后筛选至25000个基因绘制聚类热图。结题报告展示了样品间的聚类，具体如下图所示。

heatmap

1 项目简介
2 建库测序流程
3 信息分析流程
4 分析结果
5 Appendix
6 参考文献

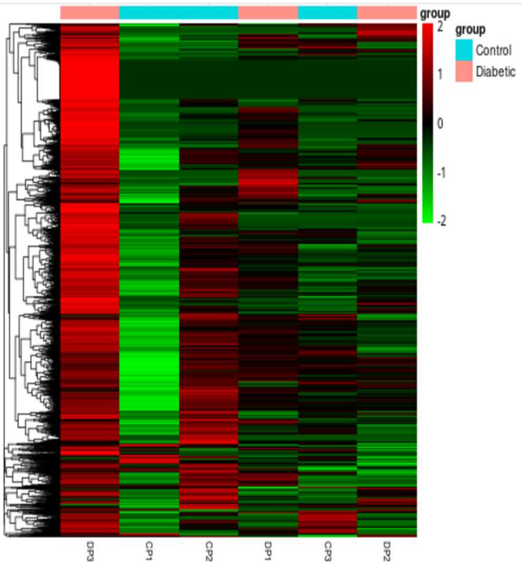

图4.17 差异表达基因聚类热图

注：图中横坐标为样本及样本的聚类结果，纵坐标为差异基因及基因的聚类结果，颜色代表了基因在样本中的表达量水平，颜色越红，表达量越高，越绿，表达量越低。

结果目录：Result/6.Differential/2.Cluster

差异分析讲解视频：链接: https://pan.baidu.com/s/1pFEC5afKbezNDVYY90nuJg 提取码: u3md

显示QA

4.8 富集分析

对于转录组分析而言，我们往往会得到成百上千的差异基因，这无疑增加了后续分析的复杂度。因此，可以将这些差异基因按照不同的功能类别进行分类，即对差异基因进行富集分析。通过富集分析，可以找到不同条件下的差异基因与哪些生物学功能或通路显著性相关，从而揭示和理解生物学过程的基本分子机制。差异基因集为差异显著分析所得差异基因并注释到GO或KEGG数据库的基因集，背景基因集为所有进行差异显著分析的基因并注释到GO或KEGG数据库的基因集。

4.8.1 GO功能富集

GO(Gene Ontology)是描述基因功能的综合性数据库(http://www.geneontology.org/)，可分为生物过程（Biological Process）和细胞组成（Cellular Component）分子功能（Molecular Function）三个部分。GO 功能显著性富集分析给出与基因组背景相比，在差异表达基因中显著富集的 GO 功能 条目，从而给出差异表达基因与哪些生物学功能显著相关。该分析首先把所有差异表达基因向 Gene Ontology 数据库的各个 term 映射， 计算每个 term 的基因数目，然后找出与整个基因组背景相比， 在差异表达基因中显著富集。GO富集分析方法为 GOseq(Young MD et al., 2010),此方法基于Wallenius non-central hyper- geometric distribution。相对于普通的Hyper- geometricdistribution，此分布的特点是从某个类别中抽取个体的概率与从某个类别之外抽取一个个体的概率是不同的，这种概率的不同是通过对基因长度的偏好性进行估计得到的，从而能更为准确地计算出 GOterm 被差异基因富集的概率。统计被显著富集的各个GOterm中的基因数，以柱状图的形式展示。GO富集以padj <= 0.05为显著富集。

4.8.1.1 GO富集列表

表4.17 差异基因GO富集结果（部分）展示

Category	GOID	Description	GeneRatio	BgRatio	pvalue	padj	Cour
CC	GO:0005840	ribosome	198|4082	386|10524	1.7324e-09	2.1482e-07	198
BP	GO:0042254	ribosome biogenesis	196|4082	400|10524	2.3296e-07	1.4444e-05	196
CC	GO:0005739	mitochondrion	183|4082	368|10524	5.169e-06	0.00021365	183
CC	GO:0043226	organelle	1235|4082	2858|10524	1.1092e-05	0.00034385	1235
MF	GO:0005198	structural molecule activity	270|4082	586|10524	9.3052e-05	0.0023077	270

显示注释

1 项目简介
2 建库测序流程
3 信息分析流程
4 分析结果
5 Appendix
6 参考文献

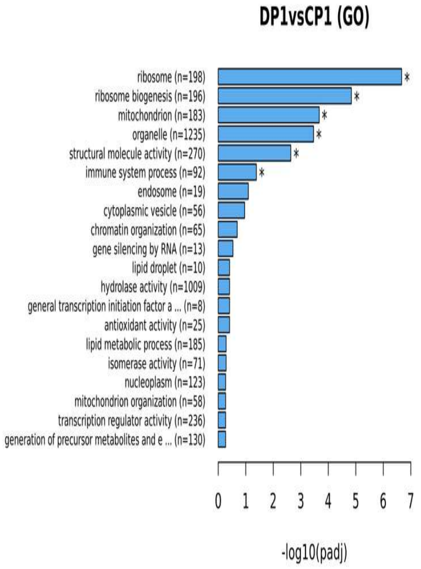

Search..
DP1vsCP1_GO_bar
DP2vsCP2_GO_bar
DP3vsCP3_GO_bar
DiabeticvsControl_GO_bar

图4.18 差异基因GO富集柱状图

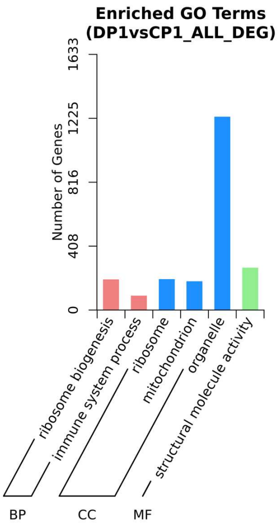

Search..
DP1vsCP1_GO_classification
DP1vsCP1_GO_updown_bar
DP2vsCP2_GO_updown_bar
DP3vsCP3_GO_classification
DP3vsCP3_GO_updown_bar
DiabeticvsControl_GO_classificati
DiabeticvsControl_GO_updown_b

图4.19 差异基因GO富集分类柱状图

注：GO分类柱状图横坐标为GO三个大类的下一层级的GO term，纵坐标为注释到该term下(包括该term的子term)的差异基因个数。3种不同分类表示Go term的三种基本分类(从左往右依次为生物学过程，细胞成分，分子功能)

1 项目简介
2 建库测序流程
3 信息分析流程
4 分析结果
5 Appendix
6 参考文献

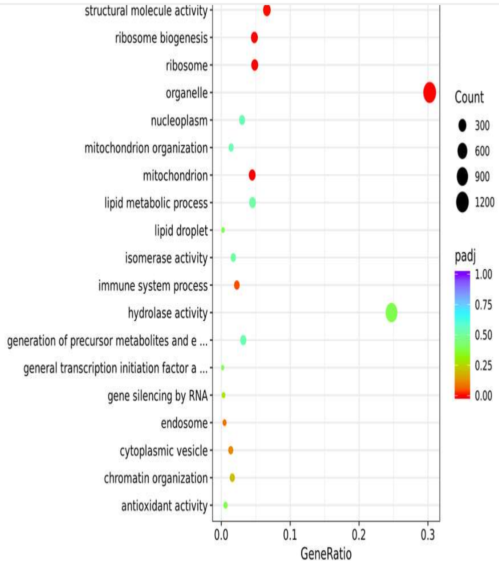

DP1vsCP1_GO_dot
DP2vsCP2_GO_dot
DP3vsCP3_GO_dot
DiabeticvsControl_GO_dot

图4.20 GO通路富集散点图

注：GO富集散点图:纵轴表示pathway名称，横轴表示GO term对应的Gene Ratio，padj的大小用点的颜色来表示，padj越小则颜色越接近红色，每个pathway下包含的差异基因的多少用点的大小来表示。

结果目录：Result/7.Enrichment/1.GO

topGO有向无环图能直观展示差异基因富集的GO term及其层级关系。有向无环图为差异基因GO富集分析的结果图形化展示方式，分支代表包含关系，从上至下所定义的功能范围越来越具体。对GO三大分类(CC细胞成分，MF 分子功能，BP生物学过程)的每一类都取富集程度最高的前10位作为有向无环图的主节点，用方框表示，并通过包含关系将相关联的GO Term一起展示，颜色的深浅代表富集程度，颜色越深代表富集程度越高。

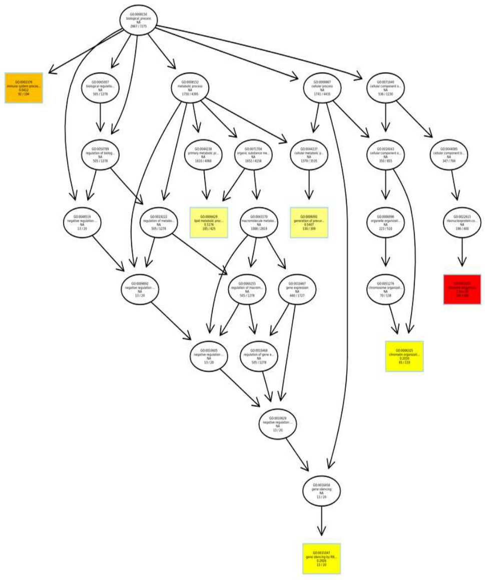

Search..
DP1vsCP1_BP
DP1vsCP1_CC
DP1vsCP1_MF
DP2vsCP2_BP
DP2vsCP2_CC
DP2vsCP2_MF
DP3vsCP3_BP
DP3vsCP3_CC
DP3vsCP3_MF
DiabeticvsControl_BP

图4.21 差异基因topGOtopGO

注：每个方框或圆圈代表一个GO term，放大方框中内容从上到下代表的含义依次为:GO term的 id、GO 的描述、 GO富集的Corrected P-Value、该GO下差异基因的数目/该GO下背景基因的数目。

结果目录：Result/7.Enrichment/1.GO/

4.8.2 KEGG通路富集

4.8.2.1 KEGG通路富集列表

1 项目简介
2 建库测序流程
3 信息分析流程
4 分析结果
5 Appendix
6 参考文献

(Kanehisa M et al., 2008)，其中整合了基因组化学和系统功能信息等众多内容。信息分析时，我们将KEGG数据库按照动物，植物，真菌等进行分类，依据研究物种的种属选择相应的类别进行分析。Pathway显著性富集分析以KEGG Pathway为单位，应用超几何检验，找出差异基因相对于所有有注释的基因显著富集的pathway，KEGG通路富集同样以padj <= 0.05作为显著性富集的阈值。

我们采用KOBAS(Mao X et al., 2005)软件对差异基因集进行KEGG功能富集分析,富集分析基于超几何分布原理。

$$P = 1 - \sum_{i=0}^{m-1} \frac{\binom{M}{i}\binom{N-M}{n-i}}{\binom{N}{n}}$$

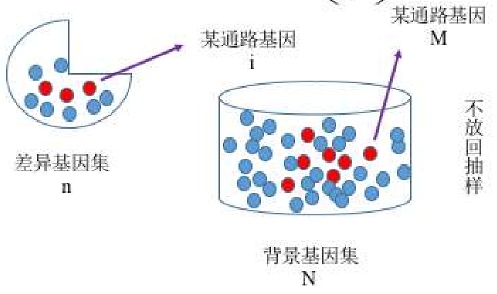

表4.18 差异基因KEGG富集结果（部分）展示

Category	KEGGID	Description	GeneRatio	BgRatio	pvalue	
KEGG	ko03010	Ribosome	116|2622	132|6680	1.92798438219e-09	6.362
KEGG	ko05016	Huntington disease	121|2622	213|6680	0.00225754581828	0.3
KEGG	ko04723	Retrograde endocannabinoid signaling	65|2622	105|6680	0.00553941964504	0.6
KEGG	ko04972	Pancreatic secretion	82|2622	148|6680	0.0158291045703	0.8
KEGG	ko00190	Oxidative phosphorylation	87|2622	160|6680	0.018418287626	0.8

注：此表仅对样本差异基因情况进行了部分展示，在网页版结题报告中，我们对该表格设置了Copy/Print/Download以及Search功能。

显示注释

差异基因KEGG富集散点图是KEGG富集分析结果的图形化展示方式。在此图中，KEGG富集程度通过Gene Ratio、padj和富集到此通路上的基因个数来衡量。其中Gene Ratio指差异表达的基因中位于该pathway条目的基因数目与所有有注释基因中位于该pathway条目的基因总数的比值。padj是做过多重假设检验校正之后的Pvalue，padj的取值范围为[0，1]，越接近于零，表示富集越显著。我们挑选了富集最显著的20条pathway条目在该图中进行展示，若富集的pathway条目不足20条，则全部展示。

1 项目简介
2 建库测序流程
3 信息分析流程
4 分析结果
5 Appendix
6 参考文献

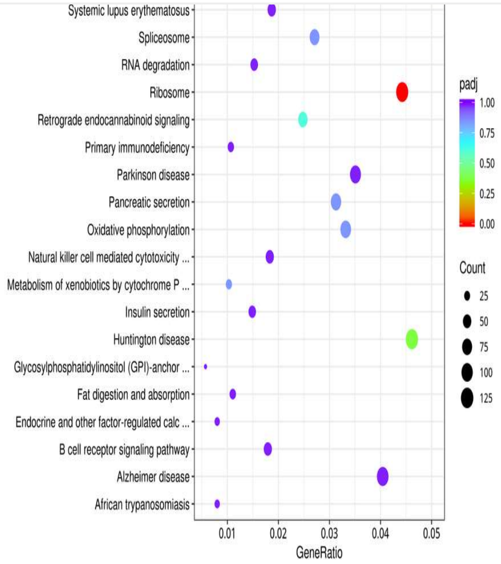

DP1vsCP1_KEGG_dot
DP2vsCP2_KEGG_dot
DP3vsCP3_KEGG_dot
DiabeticvsControl_KEGG_dot

图4.22 KEGG 通路富集散点图

注：KEGG pathway富集散点图:纵轴表示pathway名称，横轴表示pathway对应的Gene Ratio，padj的大小用点的颜色来表示，padj越小则颜色越接近红色，每个pathway下包含的差异基因的多少用点的大小来表示。

将差异基因富集出的通路图展示出来，示意如下。为了便于查看差异基因在通路图中的分布情况，我们将差异基因标注到通路图中，查看方法如下：拿到全部分析结果后，打开results中DEG_KEGGenrichment目录下后缀名为_kegg_web的子目录下的html文件，点击不同的通路名即可看到通路图，包含上调基因的KO节点边框标红色，包含下调基因的KO节点边框标绿色，包含上下调的边框标黄色。鼠标悬停于标记的KO节点，弹出差异基因细节框，标色同上，括号中数字为log2(Fold change)。以上步骤可脱机实现，如连接互联网，点击各个节点，可以连接到KEGG官方数据库中各个KO的具体信息页：

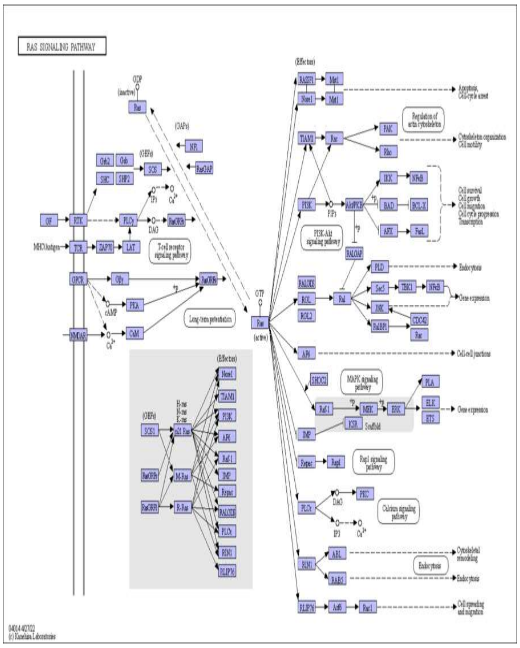

Search..
ko04014
ko04260

图4.23 KEGG 通路富集通路图

结果目录：Result/7.Enrichment/2.KEGG

4.8.3 差异基因蛋白互作网络分析

我们主要应用STRING蛋白质互作数据库(http://string-db.org/) 中的互作关系，针对数据库中包含的物种，直接从数据库中提取出目标基因集(比如差异基因list)的互作关系构建网络；针对数据库中不包含的物种，我们首先将目标基因集序列应用blastx(evalue设为1e-10)比对到string数据库中包含的参考物种蛋白质序列上，并利用参考物种的蛋白质互作关系构建互作网络。

1 项目简介
2 建库测序流程
3 信息分析流程
4 分析结果
5 Appendix
6 参考文献

性进行统计和标示作图，比如：互作网络图中节点(node)的大小与此节点的度(degree)成正比，即与此节点相连的边越多，它的度越大，节点也就越大，这些节点在网络中可能处于较为核心的位置。节点的颜色与此节点的聚集系数(clustering coefficient)相关，颜色梯度由绿到红对应聚集系数的值由低到高；聚集系数表示此节点的邻接点之间的连通性好坏，聚集系数值越高表示此节点的邻接点之间的连通性越好等等。根据不同的研究目的和需求，客户还可以在网络图中进行调整节点位置和颜色、标注表达量水平等操作。需要注意的是，通过blast比对得到的结果不能保证较好的准确性，这部分的工作只是给客户提供参考，辅助客户发现一些可能的重要的基因。按我们提供的使用说明将文件导入Cytoscape软件后的效果图如下。

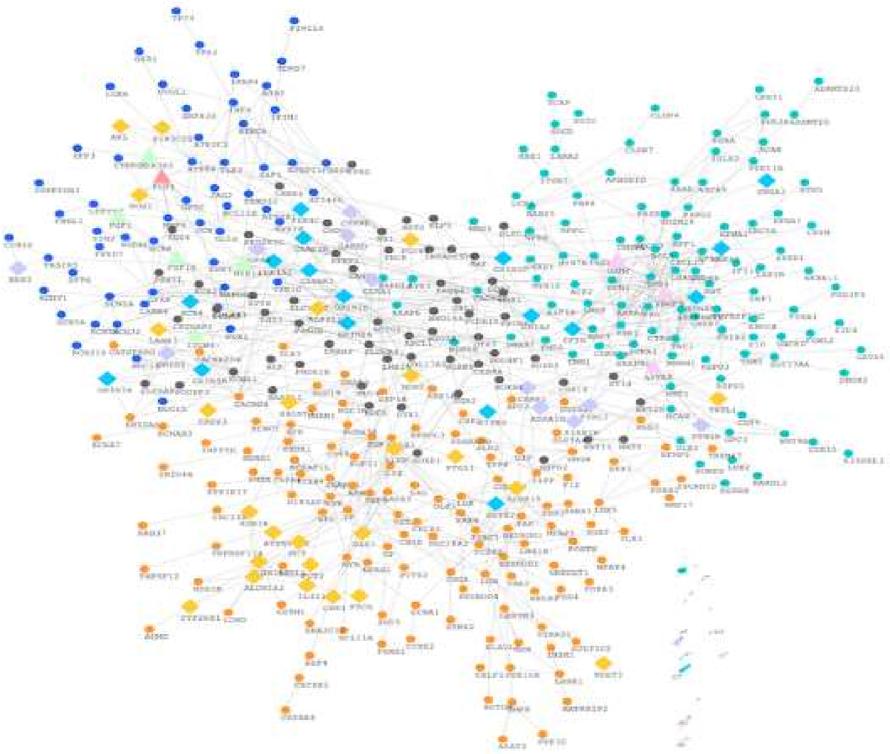

图4.24 差异基因蛋白互作网络图

注：图中每个节点表示一个蛋白，每一条连线表示相连蛋白间的相互作用

结果目录：Result/7.Enrichment/3.PPI

富集分析讲解视频：链接: https://pan.baidu.com/s/1da0_nLBRzHC4UVEY-6oeGA 提取码: fix5

蛋白互作网络分析讲解视频：链接: https://pan.baidu.com/s/1YDdd3NX_n1w3YpuiYpZanA 提取码: kxs5

显示QA

5 Appendix

5.1 软件列表

分析内容	软件	版本	参数	备注
拼接	Trinity	2.6.6	minKmerCov=3	-
			min_glue=4	
	Corset	1.09	-f ture, Default, -m 10	remove redundancy
	BUSCO	3.0.2	-m tran	-

1 项目简介
2 建库测序流程
3 信息分析流程
4 分析结果
5 Appendix
6 参考文献

基因功能注释	Diamond, KAAS	2.1.6	e-value = 1e-5	KEGG Annotation
	NCBI blast	2.9.0	e-value = 1e-5	NT Annotation
	hmmscan	HMMER 3.1	e-value = 0.01	Pfam Annotation
	blast2go	b2g4pipe_v2.5	e-value = 1e-6	GO Annotation
比对定量	RSEM	1.2.28	--estimate-rspd -mismatch-rate 0.3	mapping to Corset filtered transcriptome
SNP 检测	SAMtools/BCFtools	1.9	bcftools view varFilter -Q 20 -d 1 -D 100	-
SSR 分析	MISA, primer3	primer3-2.3.4	SSR: 1-10 2-6 3-5 4-5 5-5 6-5	Misa detect SSR, primer3 Primer Design
差异表达分析	DESeq2	1.26.0	padj <= 0.05 & $\lvert\log_2(\text{foldchange})\rvert$ >= 1	For sample with bio-replicate using DESeq2, samples without bio-replicate using EdgeR.
	edgeR	3.28.0	padj <= 0.005 & $\lvert\log_2(\text{foldchange})\rvert$ >= 1	
GO 富集	GOSeq, topGO	1.32.0, 2.32.0	Corrected P-Value <= 0.05	-
KEGG 富集	KOBAS	v3.0	Corrected P-Value <= 0.05	-
蛋白互作分析	NCBI blast 29.0	v2.2.28+	e-value = 1e-10	Using blast, String database.

5.2 Methods

研究中涉及的方法见中文版methods和英文版methods

5.3 结果文件解压方法及结果文件格式说明

压缩格式	用户类型	解压方法
*.tar	Unix/Linux/Mac 用户	使用 tar -xvf *.tar 命令打开
	Windows 用户	使用解压缩软件如 WinRAR, 7-Zip 等打开
*.gz	Unix/Linux/Mac 用户	使用 gzip –d *.gz 命令打开
	Windows 用户	使用解压缩软件如 WinRAR, 7-Zip 等打开
*.zip	Unix/Linux/Macr 用户	使用 unzip *.zip 命令打开
	Windows 用户	使用解压缩软件如 WinRAR, 7-Zip 等打开

文件类型	文件描述	打开方式
file.readme.pdf	打开结果文件前必读的结果文件解读说明文件，可以帮助您更好的理解结果文件中提供的各种类型的文件。	Windows/Mac 用户可以使用 Adobe Reader/福昕阅读器/网页浏览器等打开。
		Unix/Linux 用户使用 evince 命令打开。

1 项目简介
2 建库测序流程
3 信息分析流程
4 分析结果
5 Appendix
6 参考文献

file.fa/.fasta	列。【因文件一般较大，打开较为困难，为您提供了 sampled*.fasta 文件，方便您查看文件格式。】	Windows 用户使用高级文本编辑器 Editplus/Notepad++ 等查看。
file.fq/.fastq	高通量测序reads通常以FASTQ格式存放。【因文件一般较大，打开较为困难，为您提供了 sampled*.fastq 文件，方便您查看文件格式。】	Unix/Linux/Mac 用户使用 less 或 more 命令查看。
		Windows 用户使用高级文本编辑器 Editplus/Notepad++ 等查看。
file.vcf	包含变异检测结果的压缩文件。VCF 格式文件是一种灵活的、可扩展的用于释放 SNVs、INDELs、CNV 和 SV 结果的文本格式文件。	Unix/Linux/Mac 用户使用 less 或 more 命令查看。
		Windows 用户使用高级文本编辑器 Editplus/Notepad++ 等查看。
file.pdf/.svg	结果图像文件，矢量图，可以放大和缩小而不失真，方便用户查看和编辑处理，可使用Adobe Illustrator进行图片编辑，用于文章发表等。	Windows/Mac 用户可以使用 Adobe Reader/福昕阅读器/网页浏览器等打开。
		Unix/Linux 用户使用 evince 命令打开。
file.txt/xls	结果数据表格文件，文件以制表符（Tab）分隔。	Unix/Linux/Mac 用户使用 less 或 more 命令查看。
		Windows 用户使用高级文本编辑器 Editplus/Notepad++ 等，也可以用 Microsoft Excel 打开。
file.png	结果图像文件，位图，无损压缩。	Unix/Linux/Mac 用户使用 display 命令打开。
		Windows 用户可以使用图片浏览器打开，如 Photoshop 等。

无参转录组讲解视频汇总：链接: https://pan.baidu.com/s/1RaD2wifoZGOd0RtY-RsXgw 提取码: 6xxy

6 参考文献

Altschul SF, Madden TL, Schäffer AA, et al. Gapped BLAST and PSI-BLAST: a new generation of protein database search programs. Nucleic Acids Res. 1997;25(17):3389-3402. doi:10.1093/nar/25.17.3389

Buchfink B, Xie C, Huson DH. Fast and sensitive protein alignment using DIAMOND. Nat Methods. 2015;12(1):59-60. doi:10.1038/nmeth.3176

Chepelev I, Wei G, Tang Q, Zhao K. Detection of single nucleotide variations in expressed exons of the human genome using RNA-Seq. Nucleic Acids Res. 2009;37(16):e106. doi:10.1093/nar/gkp507

Cock PJ, Fields CJ, Goto N, Heuer ML, Rice PM. The Sanger FASTQ file format for sequences with quality scores, and the Solexa/Illumina FASTQ variants. Nucleic Acids Res. 2010;38(6):1767-1771. doi:10.1093/nar/gkp1137

Davidson NM, Oshlack A. Corset: enabling differential gene expression analysis for de novo assembled transcriptomes. Genome Biol. 2014;15(7):410. Published 2014 Jul 26. doi:10.1186/s13059-014-0410-6

Dillies MA, Rau A, Aubert J, et al. A comprehensive evaluation of normalization methods for Illumina high-throughput RNA sequencing data analysis. Brief Bioinform. 2013;14(6):671-683. doi:10.1093/bib/bbs046

ENCODE Project Consortium The ENCODE (ENCyclopedia of DNA elements) project. Science. 2004;306(5696):636-640. Finn RD, Tate J, Mistry J, et al. The Pfam protein families database. Nucleic Acids Res. 2008;36(Database issue):D281-D288. doi:10.1093/nar/gkm960

Götz S, García-Gómez JM, Terol J, et al. High-throughput functional annotation and data mining with the Blast2GO suite. Nucleic Acids Res. 2008;36(10):3420-3435. doi:10.1093/nar/gkn176

Grabherr MG, Haas BJ, Yassour M, et al. Full-length transcriptome assembly from RNA-Seq data without a reference genome. Nat Biotechnol. 2011;29(7):644-652. Published 2011 May 15. doi:10.1038/nbt.1883

Jiang L, Schlesinger F, Davis CA, et al. Synthetic spike-in standards for RNA-seq experiments. Genome Res. 2011;21(9):1543-1551. doi:10.1101/gr.121095.111

1 项目简介
2 建库测序流程
3 信息分析流程
4 分析结果
5 Appendix
6 参考文献

Kanehisa M, Araki M, Goto S, et al. KEGG for linking genomes to life and the environment. Nucleic Acids Res. 2008;36(Database issue):D480-D484. doi:10.1093/nar/gkm882

Li B, Dewey CN. RSEM: accurate transcript quantification from RNA-Seq data with or without a reference genome. BMC Bioinformatics. 2011;12:323. Published 2011 Aug 4. doi:10.1186/1471-2105-12-323

Love MI, Huber W, Anders S. Moderated estimation of fold change and dispersion for RNA-seq data with DESeq2. Genome Biol. 2014;15(12):550. doi:10.1186/s13059-014-0550-8

Mao X, Cai T, Olyarchuk JG, Wei L. Automated genome annotation and pathway identification using the KEGG Orthology (KO) as a controlled vocabulary. Bioinformatics. 2005;21(19):3787-3793. doi:10.1093/bioinformatics/bti430

Moriya Y, Itoh M, Okuda S, Yoshizawa AC, Kanehisa M. KAAS: an automatic genome annotation and pathway reconstruction server. Nucleic Acids Res. 2007;35(Web Server issue):W182-W185. doi:10.1093/nar/gkm321

Parkhomchuk D, Borodina T, Amstislavskiy V, et al. Transcriptome analysis by strand-specific sequencing of complementary DNA[J]. Nucleic acids research, 2009, 37(18): e123-e123.

Pérez-Rodríguez P, Riaño-Pachón DM, Corrêa LG, Rensing SA, Kersten B, Mueller-Roeber B. PlnTFDB: updated content and new features of the plant transcription factor database. Nucleic Acids Res. 2010;38(Database issue):D822-D827. doi:10.1093/nar/gkp805

Robinson MD, McCarthy DJ, Smyth GK. edgeR: a Bioconductor package for differential expression analysis of digital gene expression data. Bioinformatics. 2010;26(1):139-140. doi:10.1093/bioinformatics/btp616

Shannon P, Markiel A, Ozier O, et al. Cytoscape: a software environment for integrated models of biomolecular interaction networks. Genome Res. 2003;13(11):2498-2504. doi:10.1101/gr.1239303

Trapnell C, Williams BA, Pertea G, et al. Transcript assembly and quantification by RNA-Seq reveals unannotated transcripts and isoform switching during cell differentiation. Nat Biotechnol. 2010;28(5):511-515. doi:10.1038/nbt.1621

Young MD, Wakefield MJ, Smyth GK, Oshlack A. Gene ontology analysis for RNA-seq: accounting for selection bias. Genome Biol. 2010;11(2):R14. doi:10.1186/gb-2010-11-2-r14
